# Characterization of B cell receptor H-CDR3 repertoire of spleen in PRV-infected mice

**DOI:** 10.1186/s12917-022-03340-2

**Published:** 2022-06-17

**Authors:** Lishuang Deng, Fan Yang, Zhiwen Xu, Fengqin Li, Jun Zhao, Huidan Deng, Zhijie Jian, Siyuan Lai, Xiangang Sun, Ling Zhu

**Affiliations:** 1 College of Veterinary Medicine, Sichuan Agricultural University, Chengdu, China; 2 Key Laboratory of Animal Diseases and Human Health of Sichuan Province, Chengdu, China; 3 College of Animal Science, Xichang University, Xichang, China

**Keywords:** PRV, IGH, CDR3, High-throughput sequencing, Mice, Vaccine

## Abstract

Pseudorabies virus (PRV), also known as *suid Alphaherpesvirus* 1 (SuHV-1), which is one of the most devastating infectious pathogen of swine industry worldwide. Vaccination is the safest and most effective PRV prevention and control strategy. B cell receptor (BCR) is membrane-bound immunoglobulin located on the surface of B cells capable of specifically binding foreign antigens, which is one of the most important molecules regulating the proliferation and function of B cells. Here, to assess the molecular diversity of BCR H-CDR3 repertoire after different PRV strains infection, we detected the IGHV, IGHD, IGHJ genes usage and CDR3 sequence changes of mice spleen with PRV vaccine strain (Bartha-K61), variant strain (XJ) and mock infection by high-throughput sequencing. We found that PRV-infected groups shared partial BCR sequences, which are most likely to be PRV-specific BCR candidates. However, there were still differences in the IGHV genes usage as well as the combined usage of IGHV and IGHJ genes between the Bartha-K61 strain and XJ strain infection groups. In addition, the CDR3 sequences exhibited large differences in the types and lengths in PRV infection groups. Our study contributes to a better understanding of the host adaptive immune response to PRV infection and provides a theoretical basis for further research on novel and efficient PRV vaccines in the future.

**Supplementary Information:**

The online version contains supplementary material available at 10.1186/s12917-022-03340-2.

## Introduction

Pseudorabies virus (PRV), also known as *suid Alphaherpesvirus* 1 (SuHV-1), is a member of the *Alphaherpesvirinae* subfamily of the *Herpesviridae* family [1]. It is one of the most destructive swine infectious pathogens worldwide, causing Aujeszky’s diseases [2, 3]. PRV has a broad host range and is capable of infecting virtually all mammals. However, pigs or wild boar are the unique natural host [2, 4, 5]. PRV mainly damages the reproductive system, respiratory system and nervous system of pigs, generally causing diarrhea and vomiting [6]. However, pigs of different ages have different clinical symptoms: high morbidity and mortality in piglets, breathing difficulties in growing pigs, reproductive failure in breeding pigs [4, 7]. The threat posed by PRV to pig herds is mainly due to the occurrence of PRV variants in situations with issues in farm management and various breeding conditions [4]. Vaccination is currently the safest and most effective PRV prevention and control strategy, which can stimulate the host’s immune system to produce adaptive immunity and form immune memory, thereby exerts long-term immune protection. Currently, attenuated live or inactivated vaccines are developed and applied to prevent PRV infection in pigs [8]. The attenuated modified live Bartha-K61 virus is the most commonly used vaccine against PRV worldwide [8, 9].

B lymphocytes are an essential component of the adaptive immune system in humans and other vertebrates, which mainly mediate humoral immunity. B cell receptor (BCR) is membrane-bound immunoglobulin located on the surface of B cells capable of specifically binding foreign antigens, which is one of the most important molecules regulating the proliferation and function of B cells [10]. The BCR is composed of two heavy chains and two light chains, encoded by the genes IGH and IGL respectively. The antigen binding domain of the BCR consists of variable (V), diversity (D) and joining (J) gene segments in the heavy chain and V and J gene segments in the light chain [11]. The IGHV, IGHD, and IGHJ genes are often used as measures of BCR diversity and clonal evolution to describe B cell responses under different immune status [12]. In addition, the majority of variations in BCR sequences are concentrated in the complementarity determining regions (CDRs, including CDR1, CDR2, CDR3) [13]. The CDR3 is the most variable of the three CDRs and is considered a key region for determining B cells to recognize antigen peptides [10, 14]. The BCR repertoire is closely related to B cell immune response and clonal proliferation [15]. BCR repertoire sequencing can monitor the B cell response to pathogens by describing the diversity of BCR repertoire. Viral infection or vaccination has been shown to influence the diversity of the BCR repertoire [16–19], yet molecular researches of BCR repertoire after PRV infection have not been performed.

To assess the molecular diversity of BCR H-CDR3 repertoire after different PRV strains infection, we have detected usage of IGHV, IGHD, and IGHJ genes and also, CDR3 sequences of BCR of mice spleen following inoculation with PRV vaccine strain (Bartha-K61), and variant strain (XJ). Our results contribute to a better understanding of the host adaptive immune response to PRV infection and lay the foundation for further developing on new and efficient PRV vaccines.

## Materials and methods

### Viruses, mice and infections

XJ strain of PRV (GenBank accession number: MW893682) was isolated and preserved in our laboratory. Bartha-K61 strain (GenBank accession number: JF797217) was purchased from Zhongmu biological pharmaceutical Co., Ltd (Chengdu, China). PRV strains were propagated and titrated in baby hamster kidney (BHK-21) cells in DMEM supplemented with 2% FBS. With reference to the Reed-Muench method, the titers of viruses were determined by 50% tissue culture infective dose (TCID_50_) [20]. BHK-21 cells were obtained from the Animal Biotechnology Center, College of Veterinary Medicine, Sichuan Agricultural University. Female BALB/c mice (2–3 weeks old) were purchased from the Experimental Animal Corporation of Dossy (Chengdu, China). Thirty mice were randomly divided into three groups (10 mice per group), which were separately raised in different isolation rooms. Mice of group X were subcutaneously administered with 0.1 mL XJ strain (1 × 10^1.27^/0.1 mL TCID_50_), group B with 0.1 mL Bartha-K61 strain (1 × 10^3.11^/0.1 mL TCID_50_), and group C with 0.1 mL BHK-21 culture supernatant as the negative control.

### Sample collection and RNA extraction

Seventeen days post-infection, mice were sacrificed by CO_2_ asphyxiation and spleens were harvested. Total RNA of each sample was extracted using RNeasy Mini Kit (Qiagen, Germany) according to the manufacturer’s instructionsand frozen at -80 °C. RNA samples with OD (260/280) ratios in the range of 1.8–2.0 and OD (260/230) ratios from 1.8 to 2.2 detected by NanoPhotometer spectrophotometer met the sequencing requirements.

### cDNA library construction

Total RNA was reverse transcribed into cDNA using the RevertAid H Minus Kit (Thermo Scientific, USA). Based on the gene composition of BCR H-CDR3 regions, 19 upstream primers and 5 downstream primers of the mice were designed and synthesis [21]. Then cDNA was used as a template to amplify the BCR H-CDR3 regions by multiplex PCR with the specifically designed primers using the Qiagen Multiplex-PCR Kit (Qiagen, Germany). PCR cycling conditions were 95 °C for 15 min, 25 cycles of 94 °C for the 15 s and 60 °C for 3 min, and 70 °C for 10 min, followed by the final step at 4 °C as a holding temperature. Subsequently, the multiplex PCR products were purified using Beckman Agencourt AMPure XP Kit (Beckman, USA). The purified product was then subjected to a second round of multiplex PCR reaction. PCR cycling conditions were 98 °C for 1 min, 25 cycles of 98 °C for the 20 s and 65 °C for 30 s, and 72 °C for 5 min, followed by the final step at 4 °C as a holding temperature. After end-repair, dA-tailing, adapter ligation and PCR amplification, the specific DNA fragments were purified again and subjected to library construction.

### BCR repertoire sequencing

BCR repertoire sequencing was performed using the Illumina Miseq platform (Illumina, America) with a read length of 2 × 300 bp. And the raw data were obtained for further analysis. To ensure the accuracy of follow-up analysis results, high-quality clean reads were screened from raw reads by removing reads containing adapter and ploy-N, as well as low-quality reads.

### Data analysis

Raw fastq files were aligned, spliced, and filtered to obtain clean reads using PANDAseq (https://github.com/neufeld/pandaseq) [22]. BCR regions were analyzed by the international ImMunoGeneTics information system IMGT/V-Quest (http://www.IMGT.org).

## Results

### High-throughput sequencing and quality control

We analyzed the spleen samples of each group to get raw reads by Illumina Miseq high-throughput sequencing (HTS). After aligning and filtering by PANDAseq software, clean reads are obtained and saved in FASTA format. Q20 percentages of clean data for all samples were higher than 99%, Q30 percentages of clean data for all samples were higher than 97%. Then, data were submitted to IMGT V-QUEST and High V-QUEST databases for gene analysis. As shown in Table 1, the numbers of clean reads in groups X, B, and C that were successfully aligned and recognized as immune sequences in the database were 1,149,268, 1,464,064, and 1,110,979, respectively. The total number of CDR3 sequences of each group were 1,103,181, 1,402,968, and 1,063,551 respectively.

**Table 1:** BCR repertoires data using high-throughput sequencing in this study

Item	Group X	Group B	Group C
Total reads	1,345,576	1,682,460	1,288,004
Merge reads	1,186,265	1,514,862	1,167,608
Filter reads	1,155,105	1,478,122	1,137,603
Clean reads	1,149,268	1,464,064	1,110,979
Unknown sequences numebr	5837	14,058	26,624
Productive sequences number	1,110,322	1,412,649	1,070,685
Non-productive sequences number	38,946	51,415	40,294
In-frame sequences number	1,138,293	1,447,833	1,098,274
Out-of frame sequences number	10,507	15,295	11,381
Total CDR3 sequences number	1,103,181	1,402,968	1,063,551
Unique CDR3 nt sequences number	278,712	261,800	240,260
Unique CDR3 aa sequences number	241,355	226,182	210,200

C: control group; B: Bartha-K61 strain infection group; X: XJ strain infection group

### Usage of IGHV, IGHD, and IGHJ genes

**Fig. 1:**
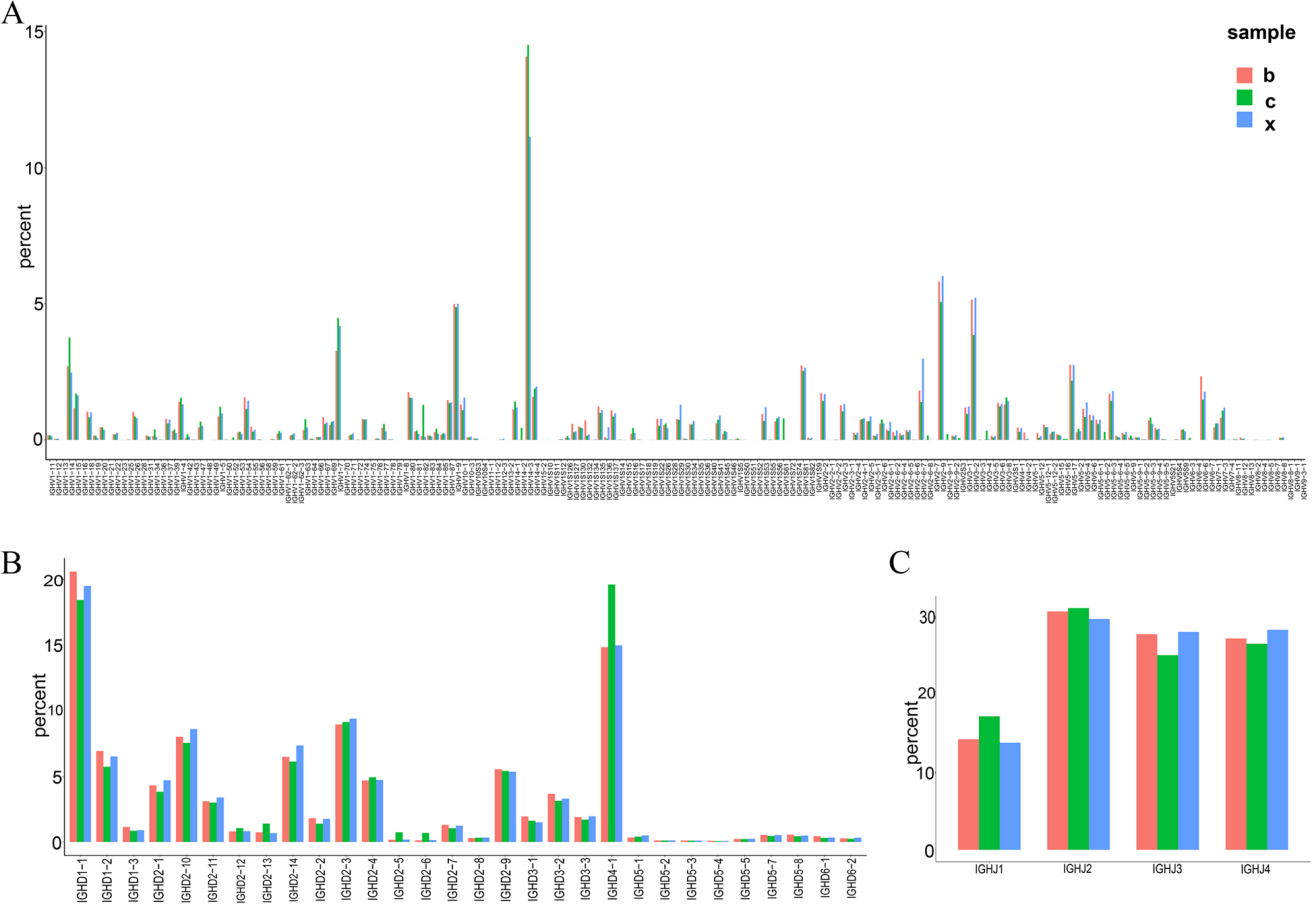
Gene usage frequencies observed for the IGHV genes (A), and IGHD genes (B) and IGHJ genes (C) of BCR in three groups. c: control group; b: Bartha-K61 strain infection group; x: XJ strain infection group

**Fig. 2:**
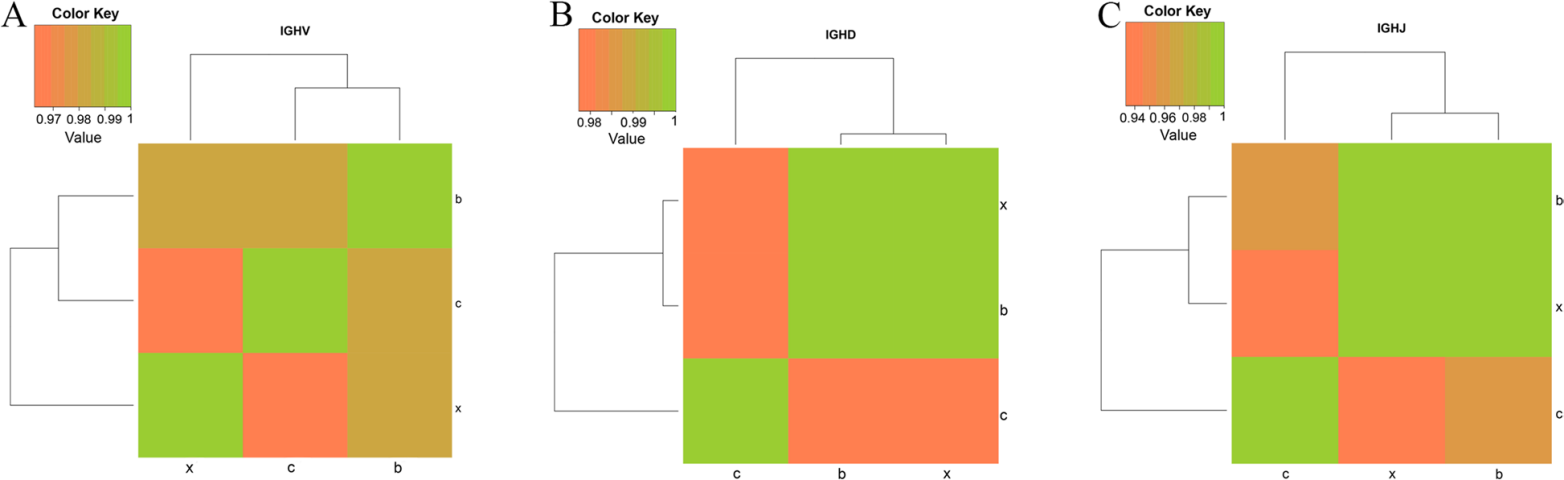
The expression correlation of IGHV genes (A), IGHD genes (B) and IGHJ genes (C) between different groups. c: control group; b: Bartha-K61 strain infection group; x: XJ strain infection group

To determine whether PRV infection alters targeting of individual IGHV, IGHD, and IGHJ genes, we generated histograms comparing usage of these genes in each group. The results showed that the frequency of individual IGHV, IGHD, and IGHJ gene usage was distinct in healthy controls and PRV-infected groups (Fig. 1, Supplementary Table 1–3). The frequency of IGHV14-3 genes in each group was the highest (11.1% in group X, 14.1% in group B and 14.5% in group C), followed by genes such as IGHV2-9 (6.0% in group X, 5.8% in group B and 5.1% in group C) and IGHV3-2 (5.2% in group X, 5.2% in group B and 3.9% in group C) (Fig. 1A, Supplementary Table 1). However, IGHV11-1, IGHV2-7 and IGHV5-16 genes were not detected in PRV-infected groups in comparison with the control group. After XJ strain infection, IGHV1-16 (0.0001%) gene was specifically used, while IGHV5-1, IGHV1-48, IGHV1-70, IGHV15-2, IGHV1S74 and IGHV9-3–1 genes were not detected. In addition, after Bartha-K61 strain infection, IGHV6-4 (0.0001%), IGHV8-7 (0.0004%), and IGHV9-1 (0.0001%) genes were explicitly utilized, while IGHV1S15, IGHV1S72 and IGHV2S3 genes were not detected. As shown in Fig. 1B and Supplementary Table 2, the use of IGHD genes in each group was similar but there is a difference in the frequency of the same gene. The IGHD1-1 gene was used most frequently (19.5% in group X, 20.6% in group B and 18.4% in group C), followed by IGHD4-1 (15.0% in group X, 14.9% in group B and 19.6% in group C), IGHD2-3 (9.4% in group X, 9.0% in group B and 9.1% in group C), and IGHD2-10 (8.6% in group X, 8.0% in group B and 7.5% in group C) genes among the three groups. Similarly, all groups used 4 types of IGHJ genes, of which the IGHJ2 gene was used the most frequently (29.8% in group X, 30.7% in group B and 31.2% in group C), and the lowest was the IGHJ1 gene (13.8% in group X, 14.3% in group B and 17.2% in group C) (Fig. 1C, Supplementary Table 3). According to the usage of IGHV, IGHD, and IGHJ genes in each group, we have drawn the above gene expression correlation heat maps. The results showed that the differences in gene use between Bartha-K61 strain infection and XJ strain infection are small, while that between XJ strain infection and control group are quite different (Fig. 2).

### Combined usage of IGHV and IGHJ genes

**Fig. 3:**
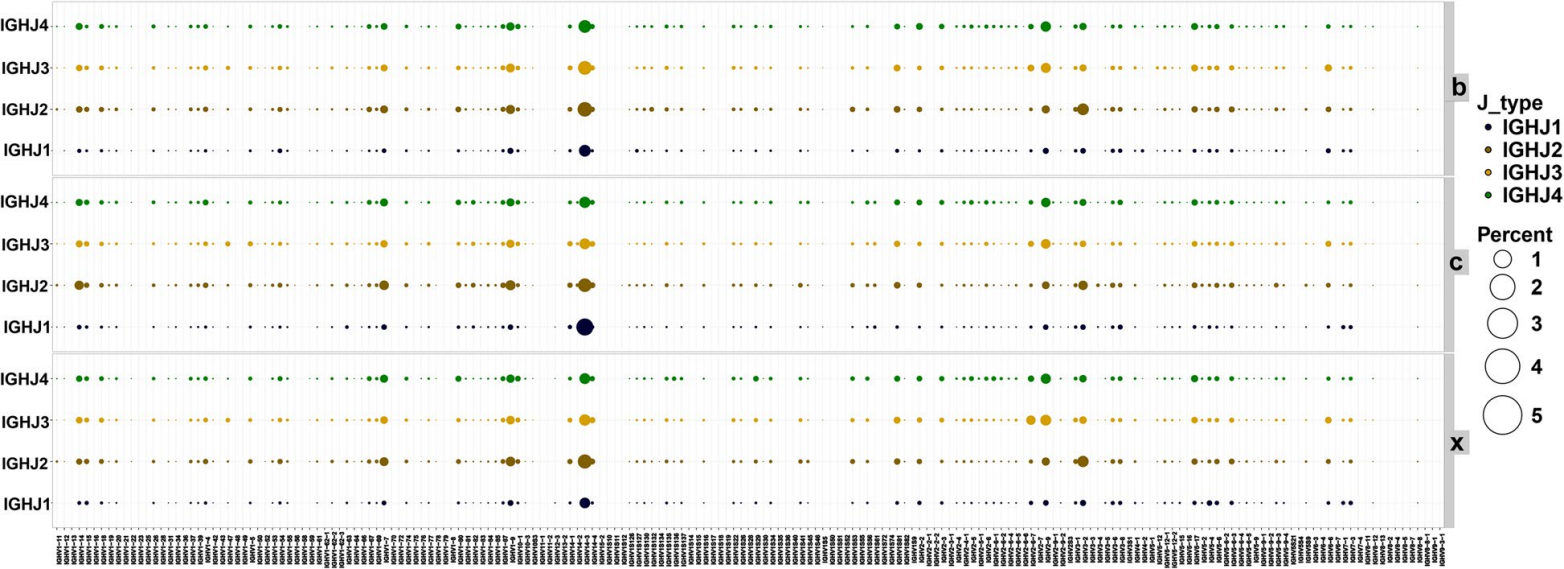
The combined usage frequencies of IGHV and IGHJ genes in three groups. c: control group; b: Bartha-K61 strain infection group; x: XJ strain infection group

To evaluate the differential combined expression of IGHV-IGHJ genes in the samples of each group, we drew a map of the combined usage frequencies of IGHV and IGHJ genes by comparing samples in the IMGT/HighV-QUEST database (Fig. 3, Supplementary Table 4). The results showed that there are many similarities in the usage of IGHV-IGHJ genes in each group, and there are also certain differences in the amount of expression. In all groups, IGHV14-3-IGHJ1 (2.2% in group X, 2.7% in group B and 5.9% in group C), IGHV14-3-IGHJ2 (4.0% in group X, 4.2% in group B and 3.7% in group C), IGHV14-3-IGHJ3 (2.6% in group X, 3.8% in group B and 2.2% in group C), IGHV14-3-IGHJ4 (2.4% in group X, 3.2% in group B and 2.5% in group C), IGHV1-9-IGHJ2 (1.7% in group X, 1.6% in group B and 1.9% in group C), IGHV1-9-IGHJ3 (1.4% in group X, 1.5% in group B and 1.2% in group C), IGHV1-9-IGHJ4 (1.3% in group X, 1.3% in group B and 1.2% in group C), IGHV1-7-IGHJ2 (1.5% in group X, 1.1% in group B and 1.7% in group C), IGHV1-7-IGHJ3 (1.1% in group X, 0.9% in group B and 1.0% in group C), IGHV1-7-IGHJ4 (1.2% in group X, 0.8% in group B and 1.2% in group C), IGHV2-9-IGHJ3 (2.3% in group X, 1.9% in group B and 1.8% in group C), IGHV2-9-IGHJ4 (2.0% in group X, 2.0% in group B and 1.7% in group C), IGHV3-2-IGHJ2 (2.5% in group X. 2.7% in group B and 1.7% in group C) and IGHV3-2-IGHJ4 (1.0% in group X, 1.0% in group B and 0.9% in group C) genes have higher expression levels (Supplementary Table 4). Compared with control group, the expression of IGHV1-14-IGHJ2 and IGHV3-2-IGHJ2 genes increased after PRV infection, while IGHV1-47-IGHJ3, IGHV1-5-IGHJ3, IGHV1-63-IGHJ1 and IGHV14-3-IGHJ1 genes expression decreased. IGHV1-11-IGHJ1, IGHV1-50-IGHJ1, IGHV1-50-IGHJ4, IGHV1-50-IGHJ1, IGHV1S61-IGHJ1, IGHV1S61-IGHJ2, IGHV1S61-IGHJ3 and IGHV1S61-IGHJ4 genes were not expressed after PRV infection. The XJ strain infection group expressed IGHV8-12-IGHJ1, IGHV12-3-IGHJ4 and IGHV1S134-IGHJ4 genes, but did not express IGHV1-12-IGHJ1, IGHV1-22-IGHJ1, IGHV1-50-IGHJ2 and IGHV1-30-IGHJ3 genes in comparison with Bartha-K61 strain infection.

### IGH CDR3 nucleotide and amino acid composition

**Fig. 4:**
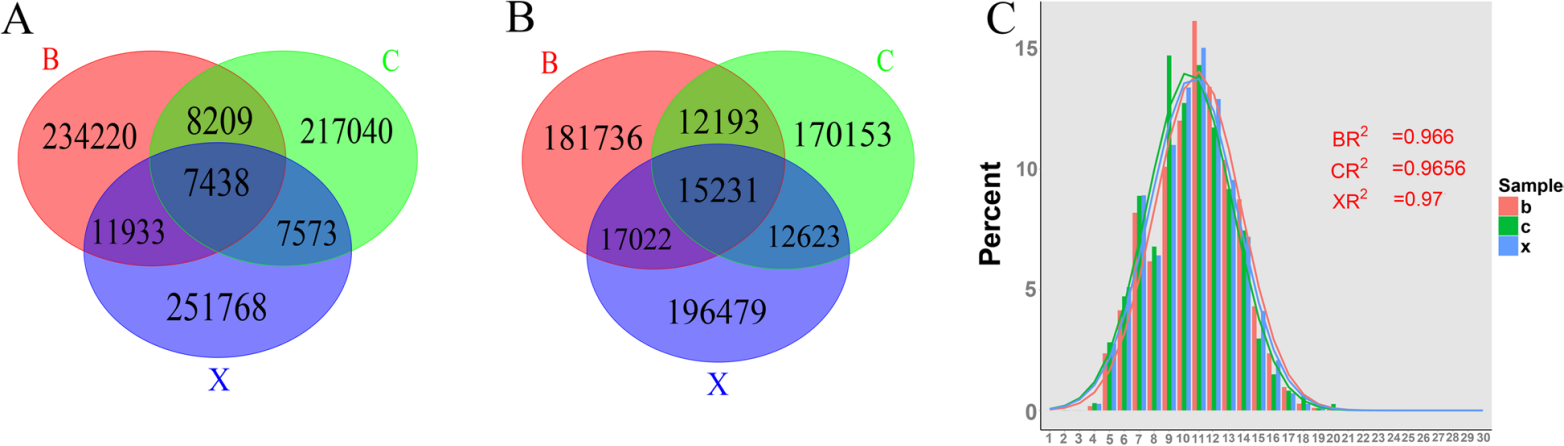
The unique CDR3 nt (A) and aa sequence (B), and CDR3 aa lengths distribution (C) of three groups. c: control group; b: Bartha-K61 strain infection group; x: XJ strain infection group

**Fig. 5:**
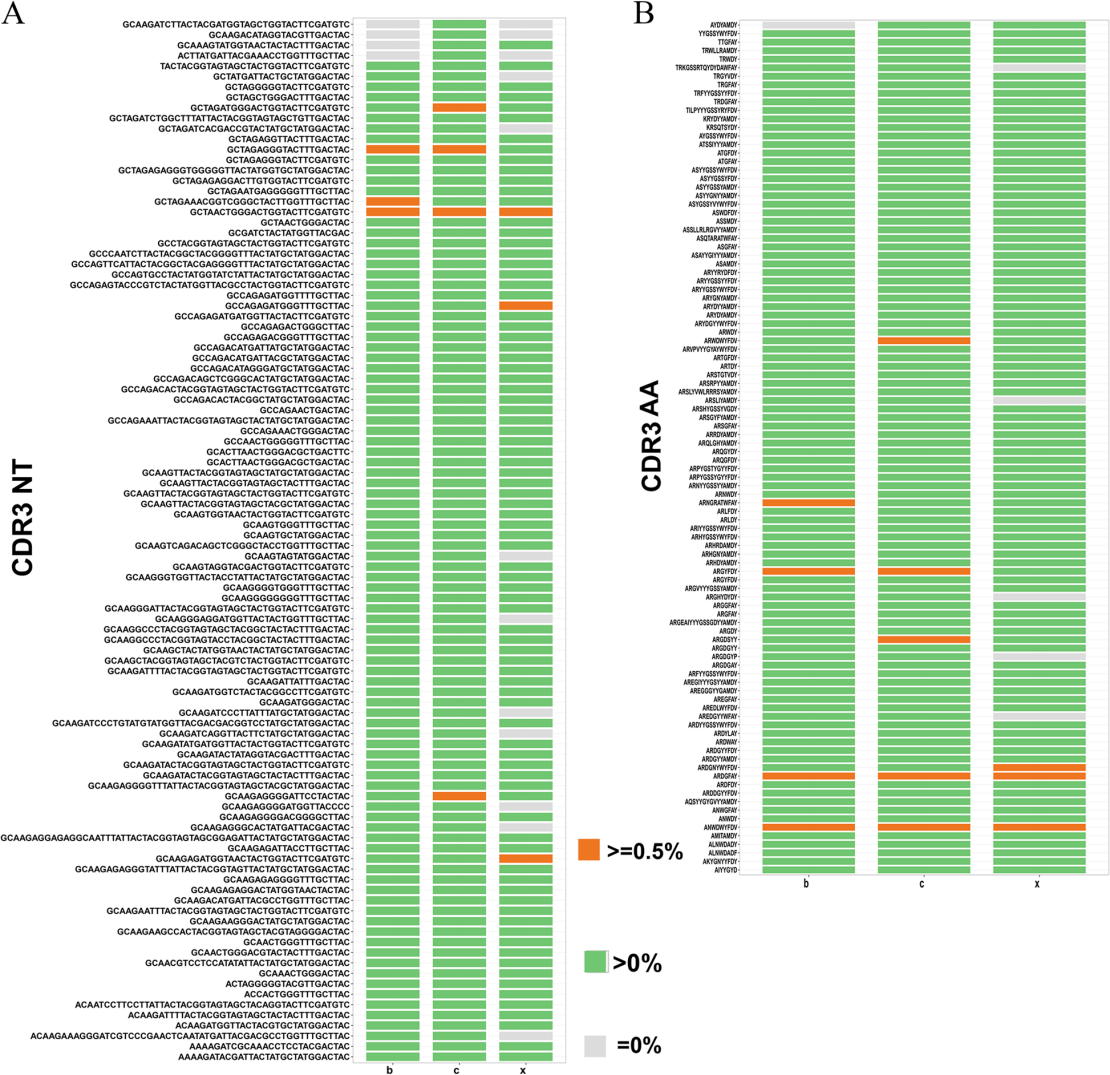
The top 100 CDR3 nt (A) and aa sequences (B) in three groups. c: control group; b: Bartha-K61 strain infection group; x: XJ strain infection group

BCR can specifically recognize antigens, which is mainly regulated by the structure of IGH CDR3. CDR3 region spatial structure depends on its nucleotide (nt) sequence and amino acid (aa) sequence. Thus, CDR3 differences among the three groups are mainly manifested in the differences in nt and aa sequence, and their expression levels. According to the comparison results of the IMGT database, we drew a venn diagram to analyze the unique CDR3 nt sequences of each group of IGH chain (Fig. 4A, Supplementary Table 5–7). The results showed that the number of shared nt sequence among three groups reached 7438, accounting for 2.67%, 2.84%, and 3.10% of the total sequences of XJ strain infection group, Bartha-K61 strain infection group, and control group, respectively. The unique CDR3 sequences of XJ strain infection group, Bartha-K61 strain infection group, and control group were 251,768, 23,420, and 217,040, respectively. Obviously, the number of shared sequences in Bartha-K61 strain infection group and control group, as well as XJ strain infection group and control group is significantly less than the number of shared sequences in Bartha-K61 strain infection group and XJ strain infection group. Next, we analyzed the top 100 CDR3 nt sequences in each group (Fig. 5A, Supplementary Table 8–10). Compared with control group, XJ strain infection group lacked 12 nt sequences and Bartha-K61 strain infection lacked 4 nt sequences (3 sequences are the same as the former group). Among the top 100 nt sequences of control group, a total of 4 sequences were high clones. The other two groups have three high cloning sequences among the top 100 nt sequences, respectively. All groups shared one high cloning sequence and PRV infection groups shared 2 of that.

Similarly, we analyzed the unique CDR3 aa sequence of each group (Fig. 4B, Supplementary Table 11–13). The number of aa sequences shared by three groups reached 15,231, accounting for 6.31%, 6.73%, and 7.25% of the total sequences of XJ strain infection group, PRV Bartha-K61 strain infection group, and control group, respectively. The numbers of unique CDR3 aa sequences in XJ strain infection group, Bartha-K61 strain infection group, and control group were 196,479, 181,736, and 170,153, respectively. Then we analyzed the CDR3 aa sequence length distribution in each group, which ranged from 2 to 20 aa lengths (Fig. 4C). In XJ strain infection group, CDR3 sequences with a length of 11 aa are the most, followed by 10 and 12 aa. In Bartha-K61 strain infection group, CDR3 sequences with a length of 11 aa are the most, followed by 12 and 10 aa. In control group, CDR3 sequences with a length of 9 aa are the most, and the proportion is close to 15%, followed by 11 and 10 aa. Compared with control group, the proportion of CDR3 sequences with a length of 9 aa differed the most in PRV infection groups, followed by 11 aa, 12 aa, and 15 aa. Then, we analyzed the top 100 CDR3 aa sequences in each group (Fig. 5B, Supplementary Table 14–16). Compared with control group, XJ strain infection group lacked 5 aa sequences and Bartha-K61 strain infection group lacked only one CDR3 aa sequence. Among the top 100 aa sequences in the control group, a total of 5 sequences showed high clones. XJ strain infection group has 3 high cloning sequences and Bartha-K61 strain infection group has 4 high cloning sequences among the top 100 aa sequences, respectively. PRV infection groups shared 2 high clones.

## Discussion

The BCR repertoire is one of the most important parts of the immune system and a key element in protecting the host from pathogens [12]. The diversity of BCR repertoire typically varies among different immune status. Therefore, understanding BCR repertoire diversity can provide insights into how the immune system rapidly responds to pathogens, thereby facilitating effective vaccine design [23]. BCR repertoire sequencing can be applied to explore BCR repertoire and to bypass in vitro screening or immunization steps [24]. In this study, we monitored the diversity of BCR H-CDR3 repertoire of PRV Bartha-K61 strain, XJ strain infected and mock infected mice spleen (group B, group X, and group C, respectively).

The frequency of the usage of IGHV, IGHD, and IGHJ genes was related to naïve rearrangement, B cell self-tolerance selection, B cell clonal proliferation, immune response [15]. The BCR repertoire responding to different infections or vaccinations has generally preference for specific IGHV, IGHD or IGHJ genes [17]. Studies have shown that the IGHV4-59, IGHV4-39, IGHV3-23, IGHV3-53, IGH3-66, IGHV2-5, and IGHV2-70 genes enriched after SARS-CoV-2 infection [17]. After Ebola virus infection, the frequency of usage of IGHV3-30 and IGHV3-15 increased [19]. The specific usage of the IGHV3-7 gene has been observed after Hepatitis B vaccination [18]. The usage of the IGHV3-23, IGHV1-2, IGHV1-18, IGHV3-33, IGHV4-39, IGHV4-31 and IGHV5-51 genes increased at various times post-Boostrix vaccination [17]. Our data indicated that PRV-infected mice shared partial BCR repertoire sequences, which are most likely to be PRV-specific BCR candidates. However, there were still differences in the IGHV genes usage and the combined usage of IGHV and IGHJ genes between the Bartha-K61 strain and XJ strain infection groups. In XJ strain infection group, IGHV1-16 and IGHV5-1 genes were specifically used. After Bartha-K61 strain infection, IGHV1-62–1, IGHV6-4, IGHV8-7, and IGHV9-1 genes were explicitly utilized. The use of IGHD, and IGHJ genes is roughly similar in different groups, thus the shared CDR3s might result from the similar selection of these genes [15]. In addition, there are a large number of IGHV-IGHJ sequences coexisted in the three groups. However, the usage of IGHV1-14-IGHJ2 and IGHV3-2-IGHJ2 increased in the PRV-infected groups compared to the control group. Furthermore, the XJ strain infection group specifically expressed IGHV8-12-IGHJ1, IGHV12-3-IGHJ4 and IGHV1S134-IGHJ4 genes, while the Bartha-K61 strain infection group specifically expressed IGHV1-12-IGHJ1, IGHV1-22-IGHJ1, IGHV1-50-IGHJ2 and IGHV1-30-IGHJ3. Overall, we speculate that the differences in IGHV, IGHD, and IGHJ gene usage between Bartha-K61 strain infection group and XJ strain infection group may be associated with strain variation and specific antigenic stimulation. Diversity in the usage of these genes might provide a direction for the discovery of biomarkers for the diagnosis and prognosis of PRV infection.

As the most variable region of the BCR repertoire, the CDR3 region determines the specificity of antigen binding to the BCR [14, 25]. The CDR3 sequences exhibited large differences in the types and lengths in PRV infection groups in this study, which may be related to the immune response to specific antigens. Compared with control group, XJ strain infection group lacked 12 nt sequences and Bartha-K61 strain infection group lacked 4 nt sequences in the top 100 CDR3 nt sequences. Length distribution of CDR3 aa sequence showed that CDR3 sequences with a length of 11 aa are the most, followed by 10 and 12 aa in XJ strain infection group. In Bartha-K61 strain infection group, CDR3 sequences with a length of 11 aa are the most, followed by 12 and 10 aa. The aa sequences of CDR3s in the PRV infection groups were more convergent compared with that of control group, especially the CDR3s with 11 aa. The length of CDR3 was reported to be associated with antibody polyreactivity and autoimmunity, and naive B cells have longer CDR3 regions than mature cells [25, 26].

Vaccine is an effective measure to prevent and control PRV infection. For most vaccines, protection is achieved via activation of B cells with vaccine antigen-specific receptors, which subsequently differentiate into plasma cells. The recognition of antigens mediated by B cells mainly depends on BCRs. XJ strain is a typical PRV variant isolated by our laboratory, which causes widespread abortions, stillbirths, and mummification in pregnant sows fetuses, persistent diarrhoea and mass mortality in nursery pigs, and severe respiratory symptoms in adult pigs [27]. Taken together, we found that the BCR H-CDR3 repertoire in XJ strain -infected and Bartha-K61 strain-infected mice is generally similar, but there are still some differences, mainly in IGHV genes uses, combined usage of IGHV and IGHJ genes, and CDR3 sequences. Therefore, we hypothesize that the classic PRV vaccine does not provide effective protection against all clinical variants. There is an urgent need for new methods and strategies to prevent PRV infection.

In conclusion, we effectively analyzed the changes of BCR H-CDR3 repertoire of PRV Bartha-K61 strain, XJ strain and mock infected mice spleen using HTS. The diversity in IGHV genes uses, combined usage of IGHV and IGHJ genes, and CDR3 sequences are found after strain and XJ strain infection. Our study contributes to a better understanding of the host adaptive immune response to PRV infection and provides a theoretical basis for further research on novel and efficient PRV vaccines. Since the current study is limited to a single dose and time point, dose- and time-dependent studies in the future may help to see clearer results of gene usage following PRV infection.

## Supplementary Information

**Additional file 1:** **Supplementary Table 1.** The frequency of IGHV genes usage. **Supplementary Table 2.** The frequency of IGHD genes usage. **Supplementary Table 3.** The frequency of IGHJ genes usage. **Supplementary Table 4.** The combined usage frequencies of IGHV and IGHJ genes. **Supplementary Table 5**. The CDR3 nt sequences of XJ strain infection group. **Supplementary Table 6**. The CDR3 nt sequences of Bartha-K61 strain infection group. **Supplementary Table 7**. The CDR3 nt sequences of control group. **Supplementary Table 8.** The top 100 CDR3 nt sequences in XJ strain infection group. **Supplementary Table 9.** The top 100 CDR3 nt sequences in Bartha-K61 strain infection group. **Supplementary Table 10.** The top 100 CDR3 nt sequences in control group. **Supplementary Table 11**. The CDR3 aa sequences of XJ strain infection group. **Supplementary Table 12**. The CDR3 aa sequences of Bartha-K61 strain infection group. **Supplementary Table 13**. The CDR3 aa sequences of control group. **Supplementary Table 14 **The top 100 CDR3 aa sequences in XJ strain infection group. **Supplementary Table 15** The top 100 CDR3 aa sequences in Bartha-K61 strain infection group. **Supplementary Table 16** The top 100 CDR3 aa sequences in control group.

**Additional file 2.**

## Data Availability

The datasets analysed during the current study are available from the corresponding author on reasonable request.

## Abbreviations

PRV: Pseudorabies virus
BCR: B cell receptor
CDR: Complementarity determining region
BHK-21: Baby hamster kidney
HTS: High-throughput sequencing
