# Supplementary material for: Characterization of B cell receptor H-CDR3 repertoire of spleen in PRV-infected mice
Source: BMC Vet Res. 2022 Jun 17;18:228. doi: 10.1186/s12917-022-03340-2 (PMC9204683; doi:10.1186/s12917-022-03340-2)
Supplement: Supplementary file 1 — Additional file 1: Supplementary Table 1. The frequency of IGHV genes usage. Supplementary Table 2. The frequency of IGHD genes usage. Supplementary Table 3. The frequency of IGHJ genes usage. Supplementary Table 4. The combined usage frequencies of IGHV and IGHJ genes. Supplementary Table 5. The CDR3 nt sequences of XJ strain infection group. Supplementary Table 6. The CDR3 nt sequences of Bartha-K61 strain infection group. Supplementary Table 7. The CDR3 nt sequences of control group. Supplementary Table 8. The top 100 CDR3 nt sequences in XJ strain infection group. Supplementary Table 9. The top 100 CDR3 nt sequences in Bartha-K61 strain infection group. Supplementary Table 10. The top 100 CDR3 nt sequences in control group. Supplementary Table 11. The CDR3 aa sequences of XJ strain infection group. Supplementary Table 12. The CDR3 aa sequences of Bartha-K61 strain infection group. Supplementary Table 13. The CDR3 aa sequences of control group. Supplementary Table 14 The top 100 CDR3 aa sequences in XJ strain infection group. Supplementary Table 15 The top 100 CDR3 aa sequences in Bartha-K61 strain infection group. Supplementary Table 16 The top 100 CDR3 aa sequences in control group. [file 12917_2022_3340_MOESM1_ESM.zip › Supplementary Tables/Supplementary tables(1-4 8-10 14-16).docx]

**Supplementary Table 1** The frequency of IGHV genes usage

| Group | Gene type | Percent |
| --- | --- | --- |
| B | IGHV1-11 | 0.1717 |
| B | IGHV1-12 | 0.0346 |
| B | IGHV1-13 | 9.00E-04 |
| B | IGHV1-14 | 2.7055 |
| B | IGHV1-15 | 1.1647 |
| B | IGHV1-18 | 1.0353 |
| B | IGHV1-19 | 0.1641 |
| B | IGHV1-20 | 0.4575 |
| B | IGHV1-21 | 2.00E-04 |
| B | IGHV1-22 | 0.2113 |
| B | IGHV1-23 | 0.002 |
| B | IGHV1-25 | 0.0149 |
| B | IGHV1-26 | 1.0194 |
| B | IGHV1-28 | 0.0024 |
| B | IGHV1-31 | 0.1662 |
| B | IGHV1-34 | 0.1407 |
| B | IGHV1-36 | 0.0156 |
| B | IGHV1-37 | 0.7733 |
| B | IGHV1-39 | 0.3254 |
| B | IGHV1-4 | 1.3981 |
| B | IGHV1-42 | 0.067 |
| B | IGHV1-43 | 0.0179 |
| B | IGHV1-47 | 0.4567 |
| B | IGHV1-48 | 0 |
| B | IGHV1-49 | 0.004 |
| B | IGHV1-5 | 0.8671 |
| B | IGHV1-50 | 0.0143 |
| B | IGHV1-52 | 0.0168 |
| B | IGHV1-53 | 0.2774 |
| B | IGHV1-54 | 1.5586 |
| B | IGHV1-55 | 0.4776 |
| B | IGHV1-56 | 0.019 |
| B | IGHV1-58 | 5.00E-04 |
| B | IGHV1-59 | 0.0309 |
| B | IGHV1-61 | 0.2262 |
| B | IGHV1-62-2 | 0.1608 |
| B | IGHV1-62-3 | 0.0045 |
| B | IGHV1-63 | 0.3643 |
| B | IGHV1-64 | 0.0435 |
| B | IGHV1-66 | 0.1017 |
| B | IGHV1-67 | 0.8358 |
| B | IGHV1-69 | 0.5453 |
| B | IGHV1-7 | 3.2747 |
| B | IGHV1-70 | 0 |
| B | IGHV1-71 | 0.1608 |
| B | IGHV1-72 | 0.005 |
| B | IGHV1-74 | 0.7623 |
| B | IGHV1-75 | 0.0022 |
| B | IGHV1-76 | 0.047 |
| B | IGHV1-77 | 0.4461 |
| B | IGHV1-78 | 0.021 |
| B | IGHV1-79 | 2.00E-04 |
| B | IGHV1-8 | 4.00E-04 |
| B | IGHV1-80 | 1.7512 |
| B | IGHV1-81 | 0.3056 |
| B | IGHV1-82 | 0.1334 |
| B | IGHV1-83 | 0.1606 |
| B | IGHV1-84 | 0.28 |
| B | IGHV1-85 | 0.1861 |
| B | IGHV1-87 | 1.4589 |
| B | IGHV1-9 | 4.987 |
| B | IGHV10-1 | 1.3001 |
| B | IGHV10-3 | 0.0914 |
| B | IGHV10S3 | 0.049 |
| B | IGHV10S4 | 3.00E-04 |
| B | IGHV11-2 | 5.00E-04 |
| B | IGHV12-3 | 0.0207 |
| B | IGHV13-2 | 0.0011 |
| B | IGHV14-1 | 1.1202 |
| B | IGHV14-2 | 0.0103 |
| B | IGHV14-3 | 14.0858 |
| B | IGHV14-4 | 1.5781 |
| B | IGHV15-2 | 1.00E-04 |
| B | IGHV1S10 | 6.00E-04 |
| B | IGHV1S11 | 3.00E-04 |
| B | IGHV1S12 | 0.0176 |
| B | IGHV1S126 | 0.0743 |
| B | IGHV1S127 | 0.5891 |
| B | IGHV1S130 | 0.49 |
| B | IGHV1S132 | 0.7186 |
| B | IGHV1S134 | 0.0071 |
| B | IGHV1S135 | 1.2337 |
| B | IGHV1S136 | 0.0944 |
| B | IGHV1S137 | 1.0873 |
| B | IGHV1S14 | 0.0107 |
| B | IGHV1S16 | 0.2281 |
| B | IGHV1S17 | 0.0133 |
| B | IGHV1S18 | 2.00E-04 |
| B | IGHV1S19 | 0.0024 |
| B | IGHV1S22 | 0.7727 |
| B | IGHV1S26 | 0.5403 |
| B | IGHV1S28 | 0.0015 |
| B | IGHV1S29 | 0.7656 |
| B | IGHV1S30 | 0.0519 |
| B | IGHV1S34 | 0.5737 |
| B | IGHV1S35 | 0.0034 |
| B | IGHV1S36 | 0.0082 |
| B | IGHV1S40 | 0.0084 |
| B | IGHV1S41 | 0.6054 |
| B | IGHV1S45 | 0.2105 |
| B | IGHV1S46 | 0.0183 |
| B | IGHV1S5 | 0.0408 |
| B | IGHV1S50 | 0.0053 |
| B | IGHV1S51 | 3.00E-04 |
| B | IGHV1S52 | 0.0012 |
| B | IGHV1S53 | 0.9481 |
| B | IGHV1S55 | 0.0146 |
| B | IGHV1S56 | 0.6805 |
| B | IGHV1S61 | 0.0038 |
| B | IGHV1S74 | 1.00E-04 |
| B | IGHV1S81 | 2.7318 |
| B | IGHV1S82 | 0.0808 |
| B | IGHV1S9 | 0.0035 |
| B | IGHV2-2 | 1.7065 |
| B | IGHV2-2-1 | 0.0028 |
| B | IGHV2-2-2 | 0.0103 |
| B | IGHV2-3 | 1.2728 |
| B | IGHV2-3-1 | 6.00E-04 |
| B | IGHV2-4 | 0.2597 |
| B | IGHV2-4-1 | 0.7412 |
| B | IGHV2-5 | 0.6776 |
| B | IGHV2-5-1 | 0.1675 |
| B | IGHV2-6 | 0.5961 |
| B | IGHV2-6-1 | 0.3731 |
| B | IGHV2-6-2 | 0.287 |
| B | IGHV2-6-4 | 0.2473 |
| B | IGHV2-6-5 | 0.3571 |
| B | IGHV2-6-6 | 0.0178 |
| B | IGHV2-6-7 | 1.8105 |
| B | IGHV2-6-8 | 0.0113 |
| B | IGHV2-9 | 5.8139 |
| B | IGHV2-9-1 | 0.0073 |
| B | IGHV2-9-2 | 0.1607 |
| B | IGHV3-1 | 1.1896 |
| B | IGHV3-2 | 5.1535 |
| B | IGHV3-3 | 1.00E-04 |
| B | IGHV3-4 | 0.0163 |
| B | IGHV3-5 | 0.1413 |
| B | IGHV3-6 | 1.3671 |
| B | IGHV3-8 | 1.2979 |
| B | IGHV3S1 | 4.00E-04 |
| B | IGHV4-1 | 0.4479 |
| B | IGHV4-2 | 0.2716 |
| B | IGHV5-12 | 0.2503 |
| B | IGHV5-12-1 | 0.5595 |
| B | IGHV5-12-2 | 0.2173 |
| B | IGHV5-15 | 0.2103 |
| B | IGHV5-17 | 2.7665 |
| B | IGHV5-2 | 0.2801 |
| B | IGHV5-4 | 1.1421 |
| B | IGHV5-6 | 0.9219 |
| B | IGHV5-6-1 | 0.7363 |
| B | IGHV5-6-2 | 0.0163 |
| B | IGHV5-6-3 | 1.694 |
| B | IGHV5-6-4 | 0.1534 |
| B | IGHV5-6-5 | 0.2575 |
| B | IGHV5-9 | 0.0799 |
| B | IGHV5-9-1 | 0.1055 |
| B | IGHV5-9-2 | 0.023 |
| B | IGHV5-9-3 | 0.7158 |
| B | IGHV5-9-4 | 0.4406 |
| B | IGHV5-9-5 | 0.0288 |
| B | IGHV5S21 | 0.0017 |
| B | IGHV5S4 | 0.0214 |
| B | IGHV5S9 | 0.371 |
| B | IGHV6-3 | 0.0118 |
| B | IGHV6-4 | 1.00E-04 |
| B | IGHV6-6 | 2.334 |
| B | IGHV6-7 | 6.00E-04 |
| B | IGHV7-1 | 0.4633 |
| B | IGHV7-3 | 0.8055 |
| B | IGHV7-4 | 2.00E-04 |
| B | IGHV8-11 | 0.0283 |
| B | IGHV8-12 | 0.0876 |
| B | IGHV8-13 | 0.0032 |
| B | IGHV8-2 | 0.0042 |
| B | IGHV8-4 | 0.0011 |
| B | IGHV8-5 | 0.0059 |
| B | IGHV8-7 | 4.00E-04 |
| B | IGHV8-8 | 0.0806 |
| B | IGHV8-8-1 | 0.0013 |
| B | IGHV9-1 | 1.00E-04 |
| B | IGHV9-3-1 | 1.00E-04 |
| C | IGHV1-11 | 0.1679 |
| C | IGHV1-12 | 0.0339 |
| C | IGHV1-13 | 9.00E-04 |
| C | IGHV1-14 | 3.7634 |
| C | IGHV1-15 | 1.7006 |
| C | IGHV1-18 | 0.8281 |
| C | IGHV1-19 | 0.1459 |
| C | IGHV1-20 | 0.4619 |
| C | IGHV1-21 | 2.00E-04 |
| C | IGHV1-22 | 0.2 |
| C | IGHV1-23 | 0.0047 |
| C | IGHV1-25 | 0.0132 |
| C | IGHV1-26 | 0.8563 |
| C | IGHV1-28 | 0.0013 |
| C | IGHV1-31 | 0.1285 |
| C | IGHV1-34 | 0.3832 |
| C | IGHV1-36 | 0.0126 |
| C | IGHV1-37 | 0.6011 |
| C | IGHV1-39 | 0.3814 |
| C | IGHV1-4 | 1.548 |
| C | IGHV1-42 | 0.2066 |
| C | IGHV1-43 | 0.0138 |
| C | IGHV1-47 | 0.6711 |
| C | IGHV1-48 | 0 |
| C | IGHV1-49 | 0.0053 |
| C | IGHV1-5 | 1.2153 |
| C | IGHV1-50 | 0.0126 |
| C | IGHV1-52 | 0.0907 |
| C | IGHV1-53 | 0.2957 |
| C | IGHV1-54 | 1.1369 |
| C | IGHV1-55 | 0.303 |
| C | IGHV1-56 | 0.0181 |
| C | IGHV1-58 | 8.00E-04 |
| C | IGHV1-59 | 0.0266 |
| C | IGHV1-61 | 0.3215 |
| C | IGHV1-62-1 | 1.00E-04 |
| C | IGHV1-62-2 | 0.1891 |
| C | IGHV1-62-3 | 0.0041 |
| C | IGHV1-63 | 0.7562 |
| C | IGHV1-64 | 0.0462 |
| C | IGHV1-66 | 0.101 |
| C | IGHV1-67 | 0.5862 |
| C | IGHV1-69 | 0.6505 |
| C | IGHV1-7 | 4.4767 |
| C | IGHV1-70 | 0.0056 |
| C | IGHV1-71 | 0.189 |
| C | IGHV1-72 | 0.0143 |
| C | IGHV1-74 | 0.7466 |
| C | IGHV1-75 | 0.002 |
| C | IGHV1-76 | 0.0498 |
| C | IGHV1-77 | 0.5816 |
| C | IGHV1-78 | 0.0169 |
| C | IGHV1-79 | 2.00E-04 |
| C | IGHV1-8 | 4.00E-04 |
| C | IGHV1-80 | 1.5514 |
| C | IGHV1-81 | 0.3413 |
| C | IGHV1-82 | 1.2829 |
| C | IGHV1-83 | 0.1551 |
| C | IGHV1-84 | 0.4098 |
| C | IGHV1-85 | 0.2398 |
| C | IGHV1-87 | 1.3558 |
| C | IGHV1-9 | 4.8748 |
| C | IGHV10-1 | 1.0942 |
| C | IGHV10-3 | 0.0925 |
| C | IGHV10S3 | 0.0495 |
| C | IGHV10S4 | 0.0017 |
| C | IGHV11-1 | 2.00E-04 |
| C | IGHV11-2 | 5.00E-04 |
| C | IGHV12-3 | 0.0151 |
| C | IGHV13-2 | 9.00E-04 |
| C | IGHV14-1 | 1.4053 |
| C | IGHV14-2 | 0.4402 |
| C | IGHV14-3 | 14.5149 |
| C | IGHV14-4 | 1.8869 |
| C | IGHV15-2 | 5.00E-04 |
| C | IGHV1S10 | 0.0036 |
| C | IGHV1S11 | 3.00E-04 |
| C | IGHV1S12 | 0.0206 |
| C | IGHV1S126 | 0.1366 |
| C | IGHV1S127 | 0.2814 |
| C | IGHV1S130 | 0.452 |
| C | IGHV1S132 | 0.1508 |
| C | IGHV1S134 | 0.0044 |
| C | IGHV1S135 | 0.9947 |
| C | IGHV1S136 | 0.0466 |
| C | IGHV1S137 | 0.8542 |
| C | IGHV1S14 | 0.0083 |
| C | IGHV1S15 | 0.0039 |
| C | IGHV1S16 | 0.4369 |
| C | IGHV1S17 | 0.012 |
| C | IGHV1S18 | 4.00E-04 |
| C | IGHV1S19 | 0.0031 |
| C | IGHV1S22 | 0.5149 |
| C | IGHV1S26 | 0.6034 |
| C | IGHV1S28 | 0.0016 |
| C | IGHV1S29 | 0.7383 |
| C | IGHV1S30 | 0.0394 |
| C | IGHV1S34 | 0.5691 |
| C | IGHV1S35 | 0.0025 |
| C | IGHV1S36 | 0.0102 |
| C | IGHV1S40 | 0.0191 |
| C | IGHV1S41 | 0.7394 |
| C | IGHV1S45 | 0.3226 |
| C | IGHV1S46 | 0.0141 |
| C | IGHV1S5 | 0.0529 |
| C | IGHV1S50 | 0.0038 |
| C | IGHV1S51 | 3.00E-04 |
| C | IGHV1S52 | 5.00E-04 |
| C | IGHV1S53 | 0.693 |
| C | IGHV1S55 | 0.016 |
| C | IGHV1S56 | 0.7871 |
| C | IGHV1S61 | 0.7941 |
| C | IGHV1S72 | 2.00E-04 |
| C | IGHV1S74 | 1.00E-04 |
| C | IGHV1S81 | 2.5329 |
| C | IGHV1S82 | 0.0356 |
| C | IGHV1S9 | 0.0029 |
| C | IGHV2-2 | 1.4315 |
| C | IGHV2-2-1 | 0.002 |
| C | IGHV2-2-2 | 0.0108 |
| C | IGHV2-3 | 1.0464 |
| C | IGHV2-3-1 | 0.001 |
| C | IGHV2-4 | 0.1906 |
| C | IGHV2-4-1 | 0.7638 |
| C | IGHV2-5 | 0.6877 |
| C | IGHV2-5-1 | 0.1404 |
| C | IGHV2-6 | 0.7482 |
| C | IGHV2-6-1 | 0.3315 |
| C | IGHV2-6-2 | 0.1479 |
| C | IGHV2-6-4 | 0.1647 |
| C | IGHV2-6-5 | 0.3002 |
| C | IGHV2-6-6 | 0.0072 |
| C | IGHV2-6-7 | 1.3951 |
| C | IGHV2-6-8 | 0.1611 |
| C | IGHV2-7 | 4.00E-04 |
| C | IGHV2-9 | 5.069 |
| C | IGHV2-9-1 | 0.2133 |
| C | IGHV2-9-2 | 0.1196 |
| C | IGHV2S3 | 0.0624 |
| C | IGHV3-1 | 0.9542 |
| C | IGHV3-2 | 3.8518 |
| C | IGHV3-3 | 0.0146 |
| C | IGHV3-4 | 0.3343 |
| C | IGHV3-5 | 0.0904 |
| C | IGHV3-6 | 1.2325 |
| C | IGHV3-8 | 1.5588 |
| C | IGHV3S1 | 0.0013 |
| C | IGHV4-1 | 0.2946 |
| C | IGHV4-2 | 0.0216 |
| C | IGHV5-12 | 0.0795 |
| C | IGHV5-12-1 | 0.4634 |
| C | IGHV5-12-2 | 0.2934 |
| C | IGHV5-15 | 0.1761 |
| C | IGHV5-16 | 0.0319 |
| C | IGHV5-17 | 2.1711 |
| C | IGHV5-2 | 0.404 |
| C | IGHV5-4 | 0.8409 |
| C | IGHV5-6 | 0.7212 |
| C | IGHV5-6-1 | 0.583 |
| C | IGHV5-6-2 | 0.2914 |
| C | IGHV5-6-3 | 1.4348 |
| C | IGHV5-6-4 | 0.1088 |
| C | IGHV5-6-5 | 0.1971 |
| C | IGHV5-9 | 0.158 |
| C | IGHV5-9-1 | 0.089 |
| C | IGHV5-9-2 | 0.0202 |
| C | IGHV5-9-3 | 0.819 |
| C | IGHV5-9-4 | 0.3689 |
| C | IGHV5-9-5 | 0.0262 |
| C | IGHV5S21 | 0.001 |
| C | IGHV5S4 | 0.0188 |
| C | IGHV5S9 | 0.3875 |
| C | IGHV6-3 | 0.072 |
| C | IGHV6-6 | 1.4765 |
| C | IGHV6-7 | 2.00E-04 |
| C | IGHV7-1 | 0.599 |
| C | IGHV7-3 | 1.0761 |
| C | IGHV7-4 | 0.0084 |
| C | IGHV8-11 | 0.0179 |
| C | IGHV8-12 | 0.0398 |
| C | IGHV8-13 | 9.00E-04 |
| C | IGHV8-2 | 0.0075 |
| C | IGHV8-4 | 0.0019 |
| C | IGHV8-5 | 0.0087 |
| C | IGHV8-8 | 0.072 |
| C | IGHV8-8-1 | 3.00E-04 |
| C | IGHV9-3-1 | 4.00E-04 |
| X | IGHV1-11 | 0.1357 |
| X | IGHV1-12 | 0.0431 |
| X | IGHV1-13 | 6.00E-04 |
| X | IGHV1-14 | 2.4755 |
| X | IGHV1-15 | 1.6353 |
| X | IGHV1-16 | 1.00E-04 |
| X | IGHV1-18 | 1.0086 |
| X | IGHV1-19 | 0.0685 |
| X | IGHV1-20 | 0.3683 |
| X | IGHV1-21 | 1.00E-04 |
| X | IGHV1-22 | 0.258 |
| X | IGHV1-23 | 0.0039 |
| X | IGHV1-25 | 0.0096 |
| X | IGHV1-26 | 0.7963 |
| X | IGHV1-28 | 0.0019 |
| X | IGHV1-31 | 0.1158 |
| X | IGHV1-34 | 0.1042 |
| X | IGHV1-36 | 0.0052 |
| X | IGHV1-37 | 0.7428 |
| X | IGHV1-39 | 0.2551 |
| X | IGHV1-4 | 1.3062 |
| X | IGHV1-42 | 0.1134 |
| X | IGHV1-43 | 0.0123 |
| X | IGHV1-47 | 0.516 |
| X | IGHV1-49 | 0.002 |
| X | IGHV1-5 | 0.971 |
| X | IGHV1-50 | 0.0048 |
| X | IGHV1-52 | 0.0092 |
| X | IGHV1-53 | 0.2122 |
| X | IGHV1-54 | 1.4417 |
| X | IGHV1-55 | 0.3702 |
| X | IGHV1-56 | 0.0132 |
| X | IGHV1-58 | 0 |
| X | IGHV1-59 | 0.0217 |
| X | IGHV1-61 | 0.2571 |
| X | IGHV1-62-2 | 0.2391 |
| X | IGHV1-62-3 | 0.0052 |
| X | IGHV1-63 | 0.4523 |
| X | IGHV1-64 | 0.0209 |
| X | IGHV1-66 | 0.1117 |
| X | IGHV1-67 | 0.6431 |
| X | IGHV1-69 | 0.6923 |
| X | IGHV1-7 | 4.1834 |
| X | IGHV1-71 | 0.2391 |
| X | IGHV1-72 | 0.0056 |
| X | IGHV1-74 | 0.755 |
| X | IGHV1-75 | 0.0019 |
| X | IGHV1-76 | 0.0345 |
| X | IGHV1-77 | 0.3163 |
| X | IGHV1-78 | 0.0239 |
| X | IGHV1-79 | 0 |
| X | IGHV1-8 | 3.00E-04 |
| X | IGHV1-80 | 1.5339 |
| X | IGHV1-81 | 0.2234 |
| X | IGHV1-82 | 0.1137 |
| X | IGHV1-83 | 0.1204 |
| X | IGHV1-84 | 0.2263 |
| X | IGHV1-85 | 0.2329 |
| X | IGHV1-87 | 1.3787 |
| X | IGHV1-9 | 4.9968 |
| X | IGHV10-1 | 1.5536 |
| X | IGHV10-3 | 0.1153 |
| X | IGHV10S3 | 0.0434 |
| X | IGHV10S4 | 2.00E-04 |
| X | IGHV11-2 | 5.00E-04 |
| X | IGHV12-3 | 0.0422 |
| X | IGHV13-2 | 0.0015 |
| X | IGHV14-1 | 1.1935 |
| X | IGHV14-2 | 0.0048 |
| X | IGHV14-3 | 11.1461 |
| X | IGHV14-4 | 1.9622 |
| X | IGHV1S10 | 5.00E-04 |
| X | IGHV1S11 | 4.00E-04 |
| X | IGHV1S12 | 0.0188 |
| X | IGHV1S126 | 0.0666 |
| X | IGHV1S127 | 0.3136 |
| X | IGHV1S130 | 0.4322 |
| X | IGHV1S132 | 0.1956 |
| X | IGHV1S134 | 0.0246 |
| X | IGHV1S135 | 1.0962 |
| X | IGHV1S136 | 0.4679 |
| X | IGHV1S137 | 0.9727 |
| X | IGHV1S14 | 0.0104 |
| X | IGHV1S15 | 0 |
| X | IGHV1S16 | 0.2511 |
| X | IGHV1S17 | 0.0072 |
| X | IGHV1S18 | 2.00E-04 |
| X | IGHV1S19 | 0.0036 |
| X | IGHV1S22 | 0.7785 |
| X | IGHV1S26 | 0.4339 |
| X | IGHV1S28 | 5.00E-04 |
| X | IGHV1S29 | 1.2972 |
| X | IGHV1S30 | 0.0282 |
| X | IGHV1S34 | 0.69 |
| X | IGHV1S35 | 0.0019 |
| X | IGHV1S36 | 0.0091 |
| X | IGHV1S40 | 0.0095 |
| X | IGHV1S41 | 0.8946 |
| X | IGHV1S45 | 0.2929 |
| X | IGHV1S46 | 0.0108 |
| X | IGHV1S5 | 0.022 |
| X | IGHV1S50 | 0.0033 |
| X | IGHV1S51 | 4.00E-04 |
| X | IGHV1S52 | 0.001 |
| X | IGHV1S53 | 1.2014 |
| X | IGHV1S55 | 0.0065 |
| X | IGHV1S56 | 0.8425 |
| X | IGHV1S61 | 0.0021 |
| X | IGHV1S72 | 1.00E-04 |
| X | IGHV1S81 | 2.6567 |
| X | IGHV1S82 | 0.0789 |
| X | IGHV1S9 | 0.0019 |
| X | IGHV2-2 | 1.6793 |
| X | IGHV2-2-1 | 0.0016 |
| X | IGHV2-2-2 | 0.0128 |
| X | IGHV2-3 | 1.3174 |
| X | IGHV2-3-1 | 5.00E-04 |
| X | IGHV2-4 | 0.2654 |
| X | IGHV2-4-1 | 0.7987 |
| X | IGHV2-5 | 0.8619 |
| X | IGHV2-5-1 | 0.214 |
| X | IGHV2-6 | 0.6032 |
| X | IGHV2-6-1 | 0.6589 |
| X | IGHV2-6-2 | 0.3471 |
| X | IGHV2-6-4 | 0.1912 |
| X | IGHV2-6-5 | 0.3625 |
| X | IGHV2-6-6 | 0.0119 |
| X | IGHV2-6-7 | 2.9835 |
| X | IGHV2-6-8 | 0.0059 |
| X | IGHV2-9 | 6.0216 |
| X | IGHV2-9-1 | 0.0052 |
| X | IGHV2-9-2 | 0.1726 |
| X | IGHV2S3 | 1.00E-04 |
| X | IGHV3-1 | 1.2292 |
| X | IGHV3-2 | 5.2222 |
| X | IGHV3-3 | 1.00E-04 |
| X | IGHV3-4 | 0.028 |
| X | IGHV3-5 | 0.1375 |
| X | IGHV3-6 | 1.3159 |
| X | IGHV3-8 | 1.425 |
| X | IGHV3S1 | 8.00E-04 |
| X | IGHV4-1 | 0.4349 |
| X | IGHV4-2 | 0.0348 |
| X | IGHV5-1 | 0 |
| X | IGHV5-12 | 0.1412 |
| X | IGHV5-12-1 | 0.4699 |
| X | IGHV5-12-2 | 0.3061 |
| X | IGHV5-15 | 0.1518 |
| X | IGHV5-17 | 2.7546 |
| X | IGHV5-2 | 0.3197 |
| X | IGHV5-4 | 1.3789 |
| X | IGHV5-6 | 0.8937 |
| X | IGHV5-6-1 | 0.741 |
| X | IGHV5-6-2 | 0.0106 |
| X | IGHV5-6-3 | 1.7946 |
| X | IGHV5-6-4 | 0.0997 |
| X | IGHV5-6-5 | 0.2887 |
| X | IGHV5-9 | 0.0614 |
| X | IGHV5-9-1 | 0.0884 |
| X | IGHV5-9-2 | 0.0175 |
| X | IGHV5-9-3 | 0.596 |
| X | IGHV5-9-4 | 0.4106 |
| X | IGHV5-9-5 | 0.0231 |
| X | IGHV5S21 | 0.0013 |
| X | IGHV5S4 | 0.0161 |
| X | IGHV5S9 | 0.3177 |
| X | IGHV6-3 | 0.003 |
| X | IGHV6-6 | 1.7694 |
| X | IGHV6-7 | 4.00E-04 |
| X | IGHV7-1 | 0.6078 |
| X | IGHV7-3 | 1.1841 |
| X | IGHV7-4 | 5.00E-04 |
| X | IGHV8-11 | 0.0169 |
| X | IGHV8-12 | 0.0583 |
| X | IGHV8-13 | 0.0019 |
| X | IGHV8-2 | 0.0084 |
| X | IGHV8-4 | 0.0012 |
| X | IGHV8-5 | 0.0066 |
| X | IGHV8-8 | 0.0956 |
| X | IGHV8-8-1 | 5.00E-04 |

C: control group; B: Bartha-K61 strain infection group; X: XJ strain infection group.

**Supplementary Table 2** The frequency of IGHD genes usage

| Group | Gene type | Percent |
| --- | --- | --- |
| B | IGHD1-1 | 20.6075 |
| B | IGHD1-2 | 6.929 |
| B | IGHD1-3 | 1.134 |
| B | IGHD2-1 | 4.313 |
| B | IGHD2-10 | 8.0156 |
| B | IGHD2-11 | 3.0998 |
| B | IGHD2-12 | 0.8053 |
| B | IGHD2-13 | 0.7305 |
| B | IGHD2-14 | 6.4794 |
| B | IGHD2-2 | 1.8064 |
| B | IGHD2-3 | 8.9515 |
| B | IGHD2-4 | 4.6844 |
| B | IGHD2-5 | 0.167 |
| B | IGHD2-6 | 0.1193 |
| B | IGHD2-7 | 1.3 |
| B | IGHD2-8 | 0.2962 |
| B | IGHD2-9 | 5.5393 |
| B | IGHD3-1 | 1.9439 |
| B | IGHD3-2 | 3.6664 |
| B | IGHD3-3 | 1.8915 |
| B | IGHD4-1 | 14.8406 |
| B | IGHD5-1 | 0.3359 |
| B | IGHD5-2 | 0.1131 |
| B | IGHD5-3 | 0.1086 |
| B | IGHD5-4 | 0.0848 |
| B | IGHD5-5 | 0.2262 |
| B | IGHD5-7 | 0.5409 |
| B | IGHD5-8 | 0.5577 |
| B | IGHD6-1 | 0.4385 |
| B | IGHD6-2 | 0.2738 |
| C | IGHD1-1 | 18.4363 |
| C | IGHD1-2 | 5.7318 |
| C | IGHD1-3 | 0.8467 |
| C | IGHD2-1 | 3.8254 |
| C | IGHD2-10 | 7.5492 |
| C | IGHD2-11 | 2.9967 |
| C | IGHD2-12 | 1.0519 |
| C | IGHD2-13 | 1.3991 |
| C | IGHD2-14 | 6.1232 |
| C | IGHD2-2 | 1.3946 |
| C | IGHD2-3 | 9.1382 |
| C | IGHD2-4 | 4.9291 |
| C | IGHD2-5 | 0.7326 |
| C | IGHD2-6 | 0.6841 |
| C | IGHD2-7 | 1.0491 |
| C | IGHD2-8 | 0.3214 |
| C | IGHD2-9 | 5.4089 |
| C | IGHD3-1 | 1.6195 |
| C | IGHD3-2 | 3.1342 |
| C | IGHD3-3 | 1.6989 |
| C | IGHD4-1 | 19.6159 |
| C | IGHD5-1 | 0.402 |
| C | IGHD5-2 | 0.1057 |
| C | IGHD5-3 | 0.0955 |
| C | IGHD5-4 | 0.0582 |
| C | IGHD5-5 | 0.2157 |
| C | IGHD5-7 | 0.4542 |
| C | IGHD5-8 | 0.4341 |
| C | IGHD6-1 | 0.3079 |
| C | IGHD6-2 | 0.2402 |
| X | IGHD1-1 | 19.507 |
| X | IGHD1-2 | 6.5138 |
| X | IGHD1-3 | 0.8972 |
| X | IGHD2-1 | 4.7 |
| X | IGHD2-10 | 8.6029 |
| X | IGHD2-11 | 3.3859 |
| X | IGHD2-12 | 0.8255 |
| X | IGHD2-13 | 0.6729 |
| X | IGHD2-14 | 7.3514 |
| X | IGHD2-2 | 1.7556 |
| X | IGHD2-3 | 9.3941 |
| X | IGHD2-4 | 4.7214 |
| X | IGHD2-5 | 0.1802 |
| X | IGHD2-6 | 0.1418 |
| X | IGHD2-7 | 1.2415 |
| X | IGHD2-8 | 0.3357 |
| X | IGHD2-9 | 5.3476 |
| X | IGHD3-1 | 1.4938 |
| X | IGHD3-2 | 3.2965 |
| X | IGHD3-3 | 1.9533 |
| X | IGHD4-1 | 14.9823 |
| X | IGHD5-1 | 0.4986 |
| X | IGHD5-2 | 0.1159 |
| X | IGHD5-3 | 0.1046 |
| X | IGHD5-4 | 0.0671 |
| X | IGHD5-5 | 0.2404 |
| X | IGHD5-7 | 0.5254 |
| X | IGHD5-8 | 0.4864 |
| X | IGHD6-1 | 0.3383 |
| X | IGHD6-2 | 0.323 |

C: control group; B: Bartha-K61 strain infection group; X: XJ strain infection group.

**Supplementary Table 3** The frequency of IGHJ genes usage

| Group | Gene type | Percent |
| --- | --- | --- |
| B | IGHJ1 | 14.2505 |
| B | IGHJ2 | 30.7275 |
| B | IGHJ3 | 27.7888 |
| B | IGHJ4 | 27.2333 |
| C | IGHJ1 | 17.2007 |
| C | IGHJ2 | 31.157 |
| C | IGHJ3 | 25.0845 |
| C | IGHJ4 | 26.5578 |
| X | IGHJ1 | 13.7954 |
| X | IGHJ2 | 29.7547 |
| X | IGHJ3 | 28.0885 |
| X | IGHJ4 | 28.3613 |

C: control group; B: Bartha-K61 strain infection group; X: XJ strain infection group.

**Supplementary Table 4** The combined usage frequencies of IGHV and IGHJ genes

| Group | V_type gene | J_type gene | Gene count | Percent |
| --- | --- | --- | --- | --- |
| b | TRBV1-11 | TRBJ1 | 67 | 0.004776 |
| b | TRBV1-11 | TRBJ2 | 1392 | 0.099218 |
| b | TRBV1-11 | TRBJ3 | 407 | 0.02901 |
| b | TRBV1-11 | TRBJ4 | 544 | 0.038775 |
| b | TRBV1-12 | TRBJ1 | 124 | 0.008838 |
| b | TRBV1-12 | TRBJ2 | 142 | 0.010121 |
| b | TRBV1-12 | TRBJ3 | 122 | 0.008696 |
| b | TRBV1-12 | TRBJ4 | 101 | 0.007199 |
| b | TRBV1-13 | TRBJ2 | 2 | 0.000143 |
| b | TRBV1-13 | TRBJ3 | 6 | 0.000428 |
| b | TRBV1-13 | TRBJ4 | 4 | 0.000285 |
| b | TRBV1-14 | TRBJ1 | 4064 | 0.289672 |
| b | TRBV1-14 | TRBJ2 | 10918 | 0.778207 |
| b | TRBV1-14 | TRBJ3 | 10507 | 0.748912 |
| b | TRBV1-14 | TRBJ4 | 12696 | 0.904939 |
| b | TRBV1-15 | TRBJ1 | 1869 | 0.133218 |
| b | TRBV1-15 | TRBJ2 | 6659 | 0.474637 |
| b | TRBV1-15 | TRBJ3 | 4757 | 0.339067 |
| b | TRBV1-15 | TRBJ4 | 3093 | 0.220461 |
| b | TRBV1-18 | TRBJ1 | 2509 | 0.178835 |
| b | TRBV1-18 | TRBJ2 | 6387 | 0.455249 |
| b | TRBV1-18 | TRBJ3 | 5770 | 0.411271 |
| b | TRBV1-18 | TRBJ4 | 5957 | 0.4246 |
| b | TRBV1-19 | TRBJ1 | 257 | 0.018318 |
| b | TRBV1-19 | TRBJ2 | 760 | 0.054171 |
| b | TRBV1-19 | TRBJ3 | 478 | 0.034071 |
| b | TRBV1-19 | TRBJ4 | 1058 | 0.075412 |
| b | TRBV1-20 | TRBJ1 | 828 | 0.059018 |
| b | TRBV1-20 | TRBJ2 | 2914 | 0.207703 |
| b | TRBV1-20 | TRBJ3 | 1638 | 0.116752 |
| b | TRBV1-20 | TRBJ4 | 1890 | 0.134714 |
| b | TRBV1-21 | TRBJ2 | 2 | 0.000143 |
| b | TRBV1-21 | TRBJ3 | 1 | 7.13E-05 |
| b | TRBV1-21 | TRBJ4 | 2 | 0.000143 |
| b | TRBV1-22 | TRBJ1 | 83 | 0.005916 |
| b | TRBV1-22 | TRBJ2 | 305 | 0.02174 |
| b | TRBV1-22 | TRBJ3 | 207 | 0.014754 |
| b | TRBV1-22 | TRBJ4 | 219 | 0.01561 |
| b | TRBV1-23 | TRBJ3 | 1 | 7.13E-05 |
| b | TRBV1-25 | TRBJ1 | 15 | 0.001069 |
| b | TRBV1-25 | TRBJ2 | 70 | 0.004989 |
| b | TRBV1-25 | TRBJ3 | 48 | 0.003421 |
| b | TRBV1-25 | TRBJ4 | 49 | 0.003493 |
| b | TRBV1-26 | TRBJ1 | 1217 | 0.086745 |
| b | TRBV1-26 | TRBJ2 | 4130 | 0.294376 |
| b | TRBV1-26 | TRBJ3 | 2835 | 0.202072 |
| b | TRBV1-26 | TRBJ4 | 3949 | 0.281475 |
| b | TRBV1-28 | TRBJ1 | 3 | 0.000214 |
| b | TRBV1-28 | TRBJ2 | 8 | 0.00057 |
| b | TRBV1-28 | TRBJ3 | 38 | 0.002709 |
| b | TRBV1-28 | TRBJ4 | 12 | 0.000855 |
| b | TRBV1-31 | TRBJ1 | 309 | 0.022025 |
| b | TRBV1-31 | TRBJ2 | 673 | 0.04797 |
| b | TRBV1-31 | TRBJ3 | 550 | 0.039203 |
| b | TRBV1-31 | TRBJ4 | 740 | 0.052745 |
| b | TRBV1-34 | TRBJ1 | 129 | 0.009195 |
| b | TRBV1-34 | TRBJ2 | 364 | 0.025945 |
| b | TRBV1-34 | TRBJ3 | 266 | 0.01896 |
| b | TRBV1-34 | TRBJ4 | 330 | 0.023522 |
| b | TRBV1-36 | TRBJ1 | 25 | 0.001782 |
| b | TRBV1-36 | TRBJ2 | 51 | 0.003635 |
| b | TRBV1-36 | TRBJ3 | 29 | 0.002067 |
| b | TRBV1-36 | TRBJ4 | 49 | 0.003493 |
| b | TRBV1-37 | TRBJ1 | 1217 | 0.086745 |
| b | TRBV1-37 | TRBJ2 | 3470 | 0.247333 |
| b | TRBV1-37 | TRBJ3 | 2150 | 0.153247 |
| b | TRBV1-37 | TRBJ4 | 3120 | 0.222386 |
| b | TRBV1-39 | TRBJ1 | 938 | 0.066858 |
| b | TRBV1-39 | TRBJ2 | 2251 | 0.160446 |
| b | TRBV1-39 | TRBJ3 | 1967 | 0.140203 |
| b | TRBV1-39 | TRBJ4 | 2915 | 0.207774 |
| b | TRBV1-4 | TRBJ1 | 2648 | 0.188743 |
| b | TRBV1-4 | TRBJ2 | 6918 | 0.493097 |
| b | TRBV1-4 | TRBJ3 | 6820 | 0.486112 |
| b | TRBV1-4 | TRBJ4 | 5379 | 0.383401 |
| b | TRBV1-42 | TRBJ1 | 136 | 0.009694 |
| b | TRBV1-42 | TRBJ2 | 320 | 0.022809 |
| b | TRBV1-42 | TRBJ3 | 270 | 0.019245 |
| b | TRBV1-42 | TRBJ4 | 231 | 0.016465 |
| b | TRBV1-43 | TRBJ1 | 13 | 0.000927 |
| b | TRBV1-43 | TRBJ2 | 58 | 0.004134 |
| b | TRBV1-43 | TRBJ3 | 43 | 0.003065 |
| b | TRBV1-43 | TRBJ4 | 59 | 0.004205 |
| b | TRBV1-47 | TRBJ1 | 290 | 0.02067 |
| b | TRBV1-47 | TRBJ2 | 1116 | 0.079546 |
| b | TRBV1-47 | TRBJ3 | 4113 | 0.293164 |
| b | TRBV1-47 | TRBJ4 | 881 | 0.062795 |
| b | TRBV1-48 | TRBJ2 | 1 | 7.13E-05 |
| b | TRBV1-49 | TRBJ4 | 1 | 7.13E-05 |
| b | TRBV1-5 | TRBJ1 | 1823 | 0.129939 |
| b | TRBV1-5 | TRBJ2 | 3241 | 0.23101 |
| b | TRBV1-5 | TRBJ3 | 3539 | 0.252251 |
| b | TRBV1-5 | TRBJ4 | 3582 | 0.255316 |
| b | TRBV1-50 | TRBJ1 | 57 | 0.004063 |
| b | TRBV1-50 | TRBJ2 | 72 | 0.005132 |
| b | TRBV1-50 | TRBJ3 | 94 | 0.0067 |
| b | TRBV1-50 | TRBJ4 | 72 | 0.005132 |
| b | TRBV1-52 | TRBJ1 | 59 | 0.004205 |
| b | TRBV1-52 | TRBJ2 | 109 | 0.007769 |
| b | TRBV1-52 | TRBJ3 | 86 | 0.00613 |
| b | TRBV1-52 | TRBJ4 | 92 | 0.006558 |
| b | TRBV1-53 | TRBJ1 | 843 | 0.060087 |
| b | TRBV1-53 | TRBJ2 | 1967 | 0.140203 |
| b | TRBV1-53 | TRBJ3 | 1459 | 0.103994 |
| b | TRBV1-53 | TRBJ4 | 1859 | 0.132505 |
| b | TRBV1-54 | TRBJ1 | 5244 | 0.373779 |
| b | TRBV1-54 | TRBJ2 | 6405 | 0.456532 |
| b | TRBV1-54 | TRBJ3 | 4990 | 0.355675 |
| b | TRBV1-54 | TRBJ4 | 5351 | 0.381406 |
| b | TRBV1-55 | TRBJ1 | 1161 | 0.082753 |
| b | TRBV1-55 | TRBJ2 | 2027 | 0.144479 |
| b | TRBV1-55 | TRBJ3 | 2030 | 0.144693 |
| b | TRBV1-55 | TRBJ4 | 1826 | 0.130153 |
| b | TRBV1-56 | TRBJ1 | 43 | 0.003065 |
| b | TRBV1-56 | TRBJ2 | 104 | 0.007413 |
| b | TRBV1-56 | TRBJ3 | 75 | 0.005346 |
| b | TRBV1-56 | TRBJ4 | 76 | 0.005417 |
| b | TRBV1-58 | TRBJ2 | 3 | 0.000214 |
| b | TRBV1-58 | TRBJ3 | 3 | 0.000214 |
| b | TRBV1-58 | TRBJ4 | 1 | 7.13E-05 |
| b | TRBV1-59 | TRBJ1 | 127 | 0.009052 |
| b | TRBV1-59 | TRBJ2 | 180 | 0.01283 |
| b | TRBV1-59 | TRBJ3 | 145 | 0.010335 |
| b | TRBV1-59 | TRBJ4 | 134 | 0.009551 |
| b | TRBV1-61 | TRBJ1 | 280 | 0.019958 |
| b | TRBV1-61 | TRBJ2 | 1103 | 0.078619 |
| b | TRBV1-61 | TRBJ3 | 997 | 0.071064 |
| b | TRBV1-61 | TRBJ4 | 748 | 0.053316 |
| b | TRBV1-62-2 | TRBJ1 | 364 | 0.025945 |
| b | TRBV1-62-2 | TRBJ2 | 1142 | 0.081399 |
| b | TRBV1-62-2 | TRBJ3 | 1896 | 0.135142 |
| b | TRBV1-62-2 | TRBJ4 | 1111 | 0.079189 |
| b | TRBV1-62-3 | TRBJ1 | 23 | 0.001639 |
| b | TRBV1-62-3 | TRBJ2 | 44 | 0.003136 |
| b | TRBV1-62-3 | TRBJ3 | 26 | 0.001853 |
| b | TRBV1-62-3 | TRBJ4 | 34 | 0.002423 |
| b | TRBV1-63 | TRBJ1 | 828 | 0.059018 |
| b | TRBV1-63 | TRBJ2 | 1680 | 0.119746 |
| b | TRBV1-63 | TRBJ3 | 1354 | 0.09651 |
| b | TRBV1-63 | TRBJ4 | 1270 | 0.090522 |
| b | TRBV1-64 | TRBJ1 | 73 | 0.005203 |
| b | TRBV1-64 | TRBJ2 | 190 | 0.013543 |
| b | TRBV1-64 | TRBJ3 | 215 | 0.015325 |
| b | TRBV1-64 | TRBJ4 | 159 | 0.011333 |
| b | TRBV1-66 | TRBJ1 | 211 | 0.01504 |
| b | TRBV1-66 | TRBJ2 | 587 | 0.04184 |
| b | TRBV1-66 | TRBJ3 | 411 | 0.029295 |
| b | TRBV1-66 | TRBJ4 | 452 | 0.032217 |
| b | TRBV1-67 | TRBJ1 | 1520 | 0.108342 |
| b | TRBV1-67 | TRBJ2 | 7634 | 0.544132 |
| b | TRBV1-67 | TRBJ3 | 3377 | 0.240704 |
| b | TRBV1-67 | TRBJ4 | 5534 | 0.394449 |
| b | TRBV1-69 | TRBJ1 | 1137 | 0.081042 |
| b | TRBV1-69 | TRBJ2 | 2834 | 0.202 |
| b | TRBV1-69 | TRBJ3 | 1742 | 0.124165 |
| b | TRBV1-69 | TRBJ4 | 1928 | 0.137423 |
| b | TRBV1-7 | TRBJ1 | 5117 | 0.364727 |
| b | TRBV1-7 | TRBJ2 | 16009 | 1.141081 |
| b | TRBV1-7 | TRBJ3 | 12511 | 0.891752 |
| b | TRBV1-7 | TRBJ4 | 11682 | 0.832663 |
| b | TRBV1-70 | TRBJ1 | 1 | 7.13E-05 |
| b | TRBV1-72 | TRBJ1 | 1 | 7.13E-05 |
| b | TRBV1-72 | TRBJ3 | 2 | 0.000143 |
| b | TRBV1-74 | TRBJ1 | 1568 | 0.111763 |
| b | TRBV1-74 | TRBJ2 | 3788 | 0.269999 |
| b | TRBV1-74 | TRBJ3 | 2348 | 0.167359 |
| b | TRBV1-74 | TRBJ4 | 3022 | 0.2154 |
| b | TRBV1-75 | TRBJ1 | 2 | 0.000143 |
| b | TRBV1-75 | TRBJ2 | 14 | 0.000998 |
| b | TRBV1-75 | TRBJ3 | 13 | 0.000927 |
| b | TRBV1-75 | TRBJ4 | 18 | 0.001283 |
| b | TRBV1-76 | TRBJ1 | 115 | 0.008197 |
| b | TRBV1-76 | TRBJ2 | 301 | 0.021455 |
| b | TRBV1-76 | TRBJ3 | 305 | 0.02174 |
| b | TRBV1-76 | TRBJ4 | 328 | 0.023379 |
| b | TRBV1-77 | TRBJ1 | 941 | 0.067072 |
| b | TRBV1-77 | TRBJ2 | 2349 | 0.167431 |
| b | TRBV1-77 | TRBJ3 | 1827 | 0.130224 |
| b | TRBV1-77 | TRBJ4 | 1517 | 0.108128 |
| b | TRBV1-78 | TRBJ1 | 38 | 0.002709 |
| b | TRBV1-78 | TRBJ2 | 104 | 0.007413 |
| b | TRBV1-78 | TRBJ3 | 90 | 0.006415 |
| b | TRBV1-78 | TRBJ4 | 87 | 0.006201 |
| b | TRBV1-79 | TRBJ1 | 1 | 7.13E-05 |
| b | TRBV1-79 | TRBJ4 | 1 | 7.13E-05 |
| b | TRBV1-8 | TRBJ1 | 1 | 7.13E-05 |
| b | TRBV1-8 | TRBJ2 | 3 | 0.000214 |
| b | TRBV1-8 | TRBJ3 | 2 | 0.000143 |
| b | TRBV1-80 | TRBJ1 | 2379 | 0.169569 |
| b | TRBV1-80 | TRBJ2 | 8294 | 0.591175 |
| b | TRBV1-80 | TRBJ3 | 5185 | 0.369574 |
| b | TRBV1-80 | TRBJ4 | 8713 | 0.621041 |
| b | TRBV1-81 | TRBJ1 | 294 | 0.020956 |
| b | TRBV1-81 | TRBJ2 | 1302 | 0.092803 |
| b | TRBV1-81 | TRBJ3 | 1242 | 0.088527 |
| b | TRBV1-81 | TRBJ4 | 1002 | 0.07142 |
| b | TRBV1-82 | TRBJ1 | 238 | 0.016964 |
| b | TRBV1-82 | TRBJ2 | 563 | 0.040129 |
| b | TRBV1-82 | TRBJ3 | 556 | 0.03963 |
| b | TRBV1-82 | TRBJ4 | 460 | 0.032788 |
| b | TRBV1-83 | TRBJ1 | 288 | 0.020528 |
| b | TRBV1-83 | TRBJ2 | 713 | 0.050821 |
| b | TRBV1-83 | TRBJ3 | 596 | 0.042481 |
| b | TRBV1-83 | TRBJ4 | 613 | 0.043693 |
| b | TRBV1-84 | TRBJ1 | 514 | 0.036637 |
| b | TRBV1-84 | TRBJ2 | 1116 | 0.079546 |
| b | TRBV1-84 | TRBJ3 | 1247 | 0.088883 |
| b | TRBV1-84 | TRBJ4 | 997 | 0.071064 |
| b | TRBV1-85 | TRBJ1 | 358 | 0.025517 |
| b | TRBV1-85 | TRBJ2 | 864 | 0.061584 |
| b | TRBV1-85 | TRBJ3 | 795 | 0.056666 |
| b | TRBV1-85 | TRBJ4 | 804 | 0.057307 |
| b | TRBV1-87 | TRBJ1 | 2405 | 0.171422 |
| b | TRBV1-87 | TRBJ2 | 6225 | 0.443702 |
| b | TRBV1-87 | TRBJ3 | 6165 | 0.439426 |
| b | TRBV1-87 | TRBJ4 | 5663 | 0.403644 |
| b | TRBV1-9 | TRBJ1 | 9244 | 0.658889 |
| b | TRBV1-9 | TRBJ2 | 21870 | 1.558838 |
| b | TRBV1-9 | TRBJ3 | 20516 | 1.462328 |
| b | TRBV1-9 | TRBJ4 | 18169 | 1.29504 |
| b | TRBV10-1 | TRBJ1 | 2166 | 0.154387 |
| b | TRBV10-1 | TRBJ2 | 4064 | 0.289672 |
| b | TRBV10-1 | TRBJ3 | 4935 | 0.351754 |
| b | TRBV10-1 | TRBJ4 | 7090 | 0.505357 |
| b | TRBV10-3 | TRBJ1 | 188 | 0.0134 |
| b | TRBV10-3 | TRBJ2 | 129 | 0.009195 |
| b | TRBV10-3 | TRBJ3 | 322 | 0.022951 |
| b | TRBV10-3 | TRBJ4 | 643 | 0.045831 |
| b | TRBV10S3 | TRBJ1 | 74 | 0.005275 |
| b | TRBV10S3 | TRBJ2 | 133 | 0.00948 |
| b | TRBV10S3 | TRBJ3 | 267 | 0.019031 |
| b | TRBV10S3 | TRBJ4 | 204 | 0.014541 |
| b | TRBV11-2 | TRBJ1 | 7 | 0.000499 |
| b | TRBV12-3 | TRBJ1 | 213 | 0.015182 |
| b | TRBV12-3 | TRBJ2 | 46 | 0.003279 |
| b | TRBV12-3 | TRBJ3 | 20 | 0.001426 |
| b | TRBV12-3 | TRBJ4 | 12 | 0.000855 |
| b | TRBV13-2 | TRBJ2 | 5 | 0.000356 |
| b | TRBV13-2 | TRBJ3 | 8 | 0.00057 |
| b | TRBV13-2 | TRBJ4 | 3 | 0.000214 |
| b | TRBV14-1 | TRBJ1 | 2424 | 0.172777 |
| b | TRBV14-1 | TRBJ2 | 5374 | 0.383045 |
| b | TRBV14-1 | TRBJ3 | 5963 | 0.425028 |
| b | TRBV14-1 | TRBJ4 | 3832 | 0.273135 |
| b | TRBV14-2 | TRBJ1 | 21 | 0.001497 |
| b | TRBV14-2 | TRBJ2 | 45 | 0.003207 |
| b | TRBV14-2 | TRBJ3 | 30 | 0.002138 |
| b | TRBV14-2 | TRBJ4 | 30 | 0.002138 |
| b | TRBV14-3 | TRBJ1 | 38486 | 2.743184 |
| b | TRBV14-3 | TRBJ2 | 59425 | 4.235663 |
| b | TRBV14-3 | TRBJ3 | 52679 | 3.754825 |
| b | TRBV14-3 | TRBJ4 | 45130 | 3.216752 |
| b | TRBV14-4 | TRBJ1 | 2337 | 0.166575 |
| b | TRBV14-4 | TRBJ2 | 6669 | 0.475349 |
| b | TRBV14-4 | TRBJ3 | 6606 | 0.470859 |
| b | TRBV14-4 | TRBJ4 | 6502 | 0.463446 |
| b | TRBV15-2 | TRBJ3 | 1 | 7.13E-05 |
| b | TRBV1S10 | TRBJ2 | 2 | 0.000143 |
| b | TRBV1S10 | TRBJ3 | 1 | 7.13E-05 |
| b | TRBV1S10 | TRBJ4 | 2 | 0.000143 |
| b | TRBV1S11 | TRBJ1 | 2 | 0.000143 |
| b | TRBV1S11 | TRBJ4 | 2 | 0.000143 |
| b | TRBV1S12 | TRBJ1 | 17 | 0.001212 |
| b | TRBV1S12 | TRBJ2 | 70 | 0.004989 |
| b | TRBV1S12 | TRBJ3 | 64 | 0.004562 |
| b | TRBV1S12 | TRBJ4 | 50 | 0.003564 |
| b | TRBV1S126 | TRBJ1 | 223 | 0.015895 |
| b | TRBV1S126 | TRBJ2 | 311 | 0.022167 |
| b | TRBV1S126 | TRBJ3 | 216 | 0.015396 |
| b | TRBV1S126 | TRBJ4 | 269 | 0.019174 |
| b | TRBV1S127 | TRBJ1 | 3114 | 0.221958 |
| b | TRBV1S127 | TRBJ2 | 1797 | 0.128086 |
| b | TRBV1S127 | TRBJ3 | 1596 | 0.113759 |
| b | TRBV1S127 | TRBJ4 | 1415 | 0.100858 |
| b | TRBV1S130 | TRBJ1 | 759 | 0.0541 |
| b | TRBV1S130 | TRBJ2 | 2504 | 0.178479 |
| b | TRBV1S130 | TRBJ3 | 1943 | 0.138492 |
| b | TRBV1S130 | TRBJ4 | 1697 | 0.120958 |
| b | TRBV1S132 | TRBJ1 | 580 | 0.041341 |
| b | TRBV1S132 | TRBJ2 | 5640 | 0.402005 |
| b | TRBV1S132 | TRBJ3 | 1675 | 0.11939 |
| b | TRBV1S132 | TRBJ4 | 1884 | 0.134287 |
| b | TRBV1S134 | TRBJ1 | 33 | 0.002352 |
| b | TRBV1S134 | TRBJ2 | 30 | 0.002138 |
| b | TRBV1S134 | TRBJ3 | 21 | 0.001497 |
| b | TRBV1S134 | TRBJ4 | 16 | 0.00114 |
| b | TRBV1S135 | TRBJ1 | 1556 | 0.110908 |
| b | TRBV1S135 | TRBJ2 | 4091 | 0.291596 |
| b | TRBV1S135 | TRBJ3 | 4024 | 0.286821 |
| b | TRBV1S135 | TRBJ4 | 3711 | 0.264511 |
| b | TRBV1S136 | TRBJ1 | 80 | 0.005702 |
| b | TRBV1S136 | TRBJ2 | 236 | 0.016821 |
| b | TRBV1S136 | TRBJ3 | 213 | 0.015182 |
| b | TRBV1S136 | TRBJ4 | 763 | 0.054385 |
| b | TRBV1S137 | TRBJ1 | 1337 | 0.095298 |
| b | TRBV1S137 | TRBJ2 | 2660 | 0.189598 |
| b | TRBV1S137 | TRBJ3 | 2136 | 0.152249 |
| b | TRBV1S137 | TRBJ4 | 2782 | 0.198294 |
| b | TRBV1S14 | TRBJ1 | 20 | 0.001426 |
| b | TRBV1S14 | TRBJ2 | 38 | 0.002709 |
| b | TRBV1S14 | TRBJ3 | 37 | 0.002637 |
| b | TRBV1S14 | TRBJ4 | 39 | 0.00278 |
| b | TRBV1S16 | TRBJ1 | 314 | 0.022381 |
| b | TRBV1S16 | TRBJ2 | 961 | 0.068498 |
| b | TRBV1S16 | TRBJ3 | 1028 | 0.073273 |
| b | TRBV1S16 | TRBJ4 | 1086 | 0.077407 |
| b | TRBV1S17 | TRBJ1 | 20 | 0.001426 |
| b | TRBV1S17 | TRBJ2 | 59 | 0.004205 |
| b | TRBV1S17 | TRBJ3 | 37 | 0.002637 |
| b | TRBV1S17 | TRBJ4 | 35 | 0.002495 |
| b | TRBV1S18 | TRBJ1 | 1 | 7.13E-05 |
| b | TRBV1S18 | TRBJ4 | 2 | 0.000143 |
| b | TRBV1S19 | TRBJ1 | 1 | 7.13E-05 |
| b | TRBV1S19 | TRBJ2 | 12 | 0.000855 |
| b | TRBV1S19 | TRBJ3 | 11 | 0.000784 |
| b | TRBV1S19 | TRBJ4 | 5 | 0.000356 |
| b | TRBV1S22 | TRBJ1 | 936 | 0.066716 |
| b | TRBV1S22 | TRBJ2 | 3195 | 0.227731 |
| b | TRBV1S22 | TRBJ3 | 3620 | 0.258024 |
| b | TRBV1S22 | TRBJ4 | 3114 | 0.221958 |
| b | TRBV1S26 | TRBJ1 | 711 | 0.050678 |
| b | TRBV1S26 | TRBJ2 | 2101 | 0.149754 |
| b | TRBV1S26 | TRBJ3 | 1811 | 0.129083 |
| b | TRBV1S26 | TRBJ4 | 1614 | 0.115042 |
| b | TRBV1S28 | TRBJ1 | 1 | 7.13E-05 |
| b | TRBV1S28 | TRBJ2 | 4 | 0.000285 |
| b | TRBV1S28 | TRBJ3 | 9 | 0.000641 |
| b | TRBV1S28 | TRBJ4 | 5 | 0.000356 |
| b | TRBV1S29 | TRBJ1 | 1456 | 0.10378 |
| b | TRBV1S29 | TRBJ2 | 3778 | 0.269286 |
| b | TRBV1S29 | TRBJ3 | 2817 | 0.200789 |
| b | TRBV1S29 | TRBJ4 | 2396 | 0.170781 |
| b | TRBV1S30 | TRBJ1 | 74 | 0.005275 |
| b | TRBV1S30 | TRBJ2 | 144 | 0.010264 |
| b | TRBV1S30 | TRBJ3 | 112 | 0.007983 |
| b | TRBV1S30 | TRBJ4 | 150 | 0.010692 |
| b | TRBV1S34 | TRBJ1 | 1383 | 0.098577 |
| b | TRBV1S34 | TRBJ2 | 2284 | 0.162798 |
| b | TRBV1S34 | TRBJ3 | 1616 | 0.115184 |
| b | TRBV1S34 | TRBJ4 | 2766 | 0.197153 |
| b | TRBV1S35 | TRBJ2 | 1 | 7.13E-05 |
| b | TRBV1S36 | TRBJ1 | 12 | 0.000855 |
| b | TRBV1S36 | TRBJ2 | 27 | 0.001924 |
| b | TRBV1S36 | TRBJ3 | 16 | 0.00114 |
| b | TRBV1S36 | TRBJ4 | 14 | 0.000998 |
| b | TRBV1S40 | TRBJ1 | 11 | 0.000784 |
| b | TRBV1S40 | TRBJ2 | 24 | 0.001711 |
| b | TRBV1S40 | TRBJ3 | 38 | 0.002709 |
| b | TRBV1S40 | TRBJ4 | 38 | 0.002709 |
| b | TRBV1S41 | TRBJ1 | 1475 | 0.105134 |
| b | TRBV1S41 | TRBJ2 | 2866 | 0.204281 |
| b | TRBV1S41 | TRBJ3 | 2048 | 0.145976 |
| b | TRBV1S41 | TRBJ4 | 2102 | 0.149825 |
| b | TRBV1S45 | TRBJ1 | 543 | 0.038704 |
| b | TRBV1S45 | TRBJ2 | 1303 | 0.092875 |
| b | TRBV1S45 | TRBJ3 | 489 | 0.034855 |
| b | TRBV1S45 | TRBJ4 | 588 | 0.041911 |
| b | TRBV1S46 | TRBJ1 | 27 | 0.001924 |
| b | TRBV1S46 | TRBJ2 | 72 | 0.005132 |
| b | TRBV1S46 | TRBJ3 | 49 | 0.003493 |
| b | TRBV1S46 | TRBJ4 | 72 | 0.005132 |
| b | TRBV1S5 | TRBJ1 | 35 | 0.002495 |
| b | TRBV1S5 | TRBJ2 | 154 | 0.010977 |
| b | TRBV1S5 | TRBJ3 | 118 | 0.008411 |
| b | TRBV1S5 | TRBJ4 | 147 | 0.010478 |
| b | TRBV1S50 | TRBJ1 | 12 | 0.000855 |
| b | TRBV1S50 | TRBJ2 | 13 | 0.000927 |
| b | TRBV1S50 | TRBJ3 | 14 | 0.000998 |
| b | TRBV1S50 | TRBJ4 | 34 | 0.002423 |
| b | TRBV1S51 | TRBJ2 | 1 | 7.13E-05 |
| b | TRBV1S51 | TRBJ3 | 1 | 7.13E-05 |
| b | TRBV1S52 | TRBJ1 | 2 | 0.000143 |
| b | TRBV1S52 | TRBJ2 | 2 | 0.000143 |
| b | TRBV1S52 | TRBJ3 | 7 | 0.000499 |
| b | TRBV1S52 | TRBJ4 | 1 | 7.13E-05 |
| b | TRBV1S53 | TRBJ1 | 1278 | 0.091093 |
| b | TRBV1S53 | TRBJ2 | 7210 | 0.513911 |
| b | TRBV1S53 | TRBJ3 | 2824 | 0.201288 |
| b | TRBV1S53 | TRBJ4 | 1949 | 0.13892 |
| b | TRBV1S55 | TRBJ1 | 25 | 0.001782 |
| b | TRBV1S55 | TRBJ2 | 44 | 0.003136 |
| b | TRBV1S55 | TRBJ3 | 78 | 0.00556 |
| b | TRBV1S55 | TRBJ4 | 27 | 0.001924 |
| b | TRBV1S56 | TRBJ1 | 1046 | 0.074556 |
| b | TRBV1S56 | TRBJ2 | 3076 | 0.219249 |
| b | TRBV1S56 | TRBJ3 | 2484 | 0.177053 |
| b | TRBV1S56 | TRBJ4 | 2499 | 0.178122 |
| b | TRBV1S61 | TRBJ1 | 2 | 0.000143 |
| b | TRBV1S61 | TRBJ2 | 5 | 0.000356 |
| b | TRBV1S61 | TRBJ3 | 2 | 0.000143 |
| b | TRBV1S61 | TRBJ4 | 3 | 0.000214 |
| b | TRBV1S74 | TRBJ3 | 1 | 7.13E-05 |
| b | TRBV1S74 | TRBJ4 | 1 | 7.13E-05 |
| b | TRBV1S81 | TRBJ1 | 3732 | 0.266007 |
| b | TRBV1S81 | TRBJ2 | 11359 | 0.809641 |
| b | TRBV1S81 | TRBJ3 | 12124 | 0.864168 |
| b | TRBV1S81 | TRBJ4 | 8666 | 0.61769 |
| b | TRBV1S82 | TRBJ1 | 106 | 0.007555 |
| b | TRBV1S82 | TRBJ2 | 477 | 0.033999 |
| b | TRBV1S82 | TRBJ3 | 233 | 0.016608 |
| b | TRBV1S82 | TRBJ4 | 317 | 0.022595 |
| b | TRBV1S9 | TRBJ1 | 11 | 0.000784 |
| b | TRBV1S9 | TRBJ2 | 14 | 0.000998 |
| b | TRBV1S9 | TRBJ3 | 12 | 0.000855 |
| b | TRBV1S9 | TRBJ4 | 10 | 0.000713 |
| b | TRBV2-2 | TRBJ1 | 2347 | 0.167288 |
| b | TRBV2-2 | TRBJ2 | 7391 | 0.526812 |
| b | TRBV2-2 | TRBJ3 | 5584 | 0.398013 |
| b | TRBV2-2 | TRBJ4 | 11852 | 0.84478 |
| b | TRBV2-2-1 | TRBJ1 | 3 | 0.000214 |
| b | TRBV2-2-1 | TRBJ2 | 7 | 0.000499 |
| b | TRBV2-2-1 | TRBJ3 | 17 | 0.001212 |
| b | TRBV2-2-1 | TRBJ4 | 20 | 0.001426 |
| b | TRBV2-2-2 | TRBJ1 | 25 | 0.001782 |
| b | TRBV2-2-2 | TRBJ2 | 34 | 0.002423 |
| b | TRBV2-2-2 | TRBJ3 | 19 | 0.001354 |
| b | TRBV2-2-2 | TRBJ4 | 37 | 0.002637 |
| b | TRBV2-3 | TRBJ1 | 2586 | 0.184324 |
| b | TRBV2-3 | TRBJ2 | 2727 | 0.194374 |
| b | TRBV2-3 | TRBJ3 | 5201 | 0.370714 |
| b | TRBV2-3 | TRBJ4 | 7352 | 0.524032 |
| b | TRBV2-3-1 | TRBJ1 | 1 | 7.13E-05 |
| b | TRBV2-3-1 | TRBJ2 | 3 | 0.000214 |
| b | TRBV2-3-1 | TRBJ3 | 5 | 0.000356 |
| b | TRBV2-3-1 | TRBJ4 | 1 | 7.13E-05 |
| b | TRBV2-4 | TRBJ1 | 443 | 0.031576 |
| b | TRBV2-4 | TRBJ2 | 1410 | 0.100501 |
| b | TRBV2-4 | TRBJ3 | 842 | 0.060016 |
| b | TRBV2-4 | TRBJ4 | 1283 | 0.091449 |
| b | TRBV2-4-1 | TRBJ1 | 790 | 0.056309 |
| b | TRBV2-4-1 | TRBJ2 | 1445 | 0.102996 |
| b | TRBV2-4-1 | TRBJ3 | 2360 | 0.168215 |
| b | TRBV2-4-1 | TRBJ4 | 2543 | 0.181259 |
| b | TRBV2-5 | TRBJ1 | 1266 | 0.090237 |
| b | TRBV2-5 | TRBJ2 | 1789 | 0.127515 |
| b | TRBV2-5 | TRBJ3 | 2219 | 0.158165 |
| b | TRBV2-5 | TRBJ4 | 4184 | 0.298225 |
| b | TRBV2-5-1 | TRBJ1 | 200 | 0.014255 |
| b | TRBV2-5-1 | TRBJ2 | 349 | 0.024876 |
| b | TRBV2-5-1 | TRBJ3 | 549 | 0.039131 |
| b | TRBV2-5-1 | TRBJ4 | 1244 | 0.088669 |
| b | TRBV2-6 | TRBJ1 | 1258 | 0.089667 |
| b | TRBV2-6 | TRBJ2 | 1411 | 0.100573 |
| b | TRBV2-6 | TRBJ3 | 1846 | 0.131578 |
| b | TRBV2-6 | TRBJ4 | 4041 | 0.288032 |
| b | TRBV2-6-1 | TRBJ1 | 1022 | 0.072846 |
| b | TRBV2-6-1 | TRBJ2 | 609 | 0.043408 |
| b | TRBV2-6-1 | TRBJ3 | 979 | 0.069781 |
| b | TRBV2-6-1 | TRBJ4 | 2570 | 0.183183 |
| b | TRBV2-6-2 | TRBJ1 | 678 | 0.048326 |
| b | TRBV2-6-2 | TRBJ2 | 480 | 0.034213 |
| b | TRBV2-6-2 | TRBJ3 | 641 | 0.045689 |
| b | TRBV2-6-2 | TRBJ4 | 2206 | 0.157238 |
| b | TRBV2-6-4 | TRBJ1 | 627 | 0.044691 |
| b | TRBV2-6-4 | TRBJ2 | 661 | 0.047114 |
| b | TRBV2-6-4 | TRBJ3 | 965 | 0.068783 |
| b | TRBV2-6-4 | TRBJ4 | 1286 | 0.091663 |
| b | TRBV2-6-5 | TRBJ1 | 611 | 0.043551 |
| b | TRBV2-6-5 | TRBJ2 | 884 | 0.063009 |
| b | TRBV2-6-5 | TRBJ3 | 1934 | 0.137851 |
| b | TRBV2-6-5 | TRBJ4 | 1573 | 0.112119 |
| b | TRBV2-6-6 | TRBJ1 | 22 | 0.001568 |
| b | TRBV2-6-6 | TRBJ2 | 57 | 0.004063 |
| b | TRBV2-6-6 | TRBJ3 | 84 | 0.005987 |
| b | TRBV2-6-6 | TRBJ4 | 86 | 0.00613 |
| b | TRBV2-6-7 | TRBJ1 | 2260 | 0.161087 |
| b | TRBV2-6-7 | TRBJ2 | 3167 | 0.225736 |
| b | TRBV2-6-7 | TRBJ3 | 12261 | 0.873933 |
| b | TRBV2-6-7 | TRBJ4 | 7556 | 0.538573 |
| b | TRBV2-9 | TRBJ1 | 9191 | 0.655111 |
| b | TRBV2-9 | TRBJ2 | 17151 | 1.22248 |
| b | TRBV2-9 | TRBJ3 | 26639 | 1.89876 |
| b | TRBV2-9 | TRBJ4 | 28516 | 2.032548 |
| b | TRBV2-9-1 | TRBJ1 | 12 | 0.000855 |
| b | TRBV2-9-1 | TRBJ2 | 14 | 0.000998 |
| b | TRBV2-9-1 | TRBJ3 | 45 | 0.003207 |
| b | TRBV2-9-1 | TRBJ4 | 31 | 0.00221 |
| b | TRBV2-9-2 | TRBJ1 | 229 | 0.016323 |
| b | TRBV2-9-2 | TRBJ2 | 368 | 0.02623 |
| b | TRBV2-9-2 | TRBJ3 | 941 | 0.067072 |
| b | TRBV2-9-2 | TRBJ4 | 686 | 0.048896 |
| b | TRBV3-1 | TRBJ1 | 2873 | 0.20478 |
| b | TRBV3-1 | TRBJ2 | 5826 | 0.415263 |
| b | TRBV3-1 | TRBJ3 | 3712 | 0.264582 |
| b | TRBV3-1 | TRBJ4 | 4312 | 0.307348 |
| b | TRBV3-2 | TRBJ1 | 7855 | 0.559884 |
| b | TRBV3-2 | TRBJ2 | 38112 | 2.716527 |
| b | TRBV3-2 | TRBJ3 | 12977 | 0.924968 |
| b | TRBV3-2 | TRBJ4 | 13510 | 0.962959 |
| b | TRBV3-3 | TRBJ4 | 1 | 7.13E-05 |
| b | TRBV3-4 | TRBJ1 | 29 | 0.002067 |
| b | TRBV3-4 | TRBJ2 | 108 | 0.007698 |
| b | TRBV3-4 | TRBJ3 | 38 | 0.002709 |
| b | TRBV3-4 | TRBJ4 | 53 | 0.003778 |
| b | TRBV3-5 | TRBJ1 | 305 | 0.02174 |
| b | TRBV3-5 | TRBJ2 | 1234 | 0.087956 |
| b | TRBV3-5 | TRBJ3 | 195 | 0.013899 |
| b | TRBV3-5 | TRBJ4 | 249 | 0.017748 |
| b | TRBV3-6 | TRBJ1 | 4237 | 0.302003 |
| b | TRBV3-6 | TRBJ2 | 6150 | 0.438356 |
| b | TRBV3-6 | TRBJ3 | 5060 | 0.360664 |
| b | TRBV3-6 | TRBJ4 | 3546 | 0.25275 |
| b | TRBV3-8 | TRBJ1 | 4436 | 0.316187 |
| b | TRBV3-8 | TRBJ2 | 5417 | 0.38611 |
| b | TRBV3-8 | TRBJ3 | 3933 | 0.280334 |
| b | TRBV3-8 | TRBJ4 | 4428 | 0.315617 |
| b | TRBV3S1 | TRBJ2 | 1 | 7.13E-05 |
| b | TRBV4-1 | TRBJ1 | 1030 | 0.073416 |
| b | TRBV4-1 | TRBJ2 | 1484 | 0.105776 |
| b | TRBV4-1 | TRBJ3 | 2110 | 0.150395 |
| b | TRBV4-1 | TRBJ4 | 1660 | 0.118321 |
| b | TRBV4-2 | TRBJ1 | 2604 | 0.185607 |
| b | TRBV4-2 | TRBJ2 | 194 | 0.013828 |
| b | TRBV4-2 | TRBJ3 | 181 | 0.012901 |
| b | TRBV4-2 | TRBJ4 | 831 | 0.059232 |
| b | TRBV5-12 | TRBJ1 | 250 | 0.017819 |
| b | TRBV5-12 | TRBJ2 | 509 | 0.03628 |
| b | TRBV5-12 | TRBJ3 | 2107 | 0.150182 |
| b | TRBV5-12 | TRBJ4 | 755 | 0.053814 |
| b | TRBV5-12-1 | TRBJ1 | 1042 | 0.074271 |
| b | TRBV5-12-1 | TRBJ2 | 2402 | 0.171208 |
| b | TRBV5-12-1 | TRBJ3 | 2533 | 0.180546 |
| b | TRBV5-12-1 | TRBJ4 | 2050 | 0.146119 |
| b | TRBV5-12-2 | TRBJ1 | 420 | 0.029937 |
| b | TRBV5-12-2 | TRBJ2 | 769 | 0.054812 |
| b | TRBV5-12-2 | TRBJ3 | 840 | 0.059873 |
| b | TRBV5-12-2 | TRBJ4 | 978 | 0.069709 |
| b | TRBV5-15 | TRBJ1 | 1315 | 0.09373 |
| b | TRBV5-15 | TRBJ2 | 620 | 0.044192 |
| b | TRBV5-15 | TRBJ3 | 468 | 0.033358 |
| b | TRBV5-15 | TRBJ4 | 547 | 0.038989 |
| b | TRBV5-17 | TRBJ1 | 3626 | 0.258452 |
| b | TRBV5-17 | TRBJ2 | 10168 | 0.724749 |
| b | TRBV5-17 | TRBJ3 | 12426 | 0.885694 |
| b | TRBV5-17 | TRBJ4 | 12577 | 0.896457 |
| b | TRBV5-2 | TRBJ1 | 511 | 0.036423 |
| b | TRBV5-2 | TRBJ2 | 760 | 0.054171 |
| b | TRBV5-2 | TRBJ3 | 1407 | 0.100287 |
| b | TRBV5-2 | TRBJ4 | 1263 | 0.090023 |
| b | TRBV5-4 | TRBJ1 | 3676 | 0.262016 |
| b | TRBV5-4 | TRBJ2 | 3718 | 0.26501 |
| b | TRBV5-4 | TRBJ3 | 5033 | 0.358739 |
| b | TRBV5-4 | TRBJ4 | 3696 | 0.263442 |
| b | TRBV5-6 | TRBJ1 | 2956 | 0.210696 |
| b | TRBV5-6 | TRBJ2 | 7634 | 0.544132 |
| b | TRBV5-6 | TRBJ3 | 6321 | 0.450545 |
| b | TRBV5-6 | TRBJ4 | 6786 | 0.483689 |
| b | TRBV5-6-2 | TRBJ1 | 59 | 0.004205 |
| b | TRBV5-6-2 | TRBJ2 | 40 | 0.002851 |
| b | TRBV5-6-2 | TRBJ3 | 58 | 0.004134 |
| b | TRBV5-6-2 | TRBJ4 | 68 | 0.004847 |
| b | TRBV5-6-3 | TRBJ1 | 2814 | 0.200575 |
| b | TRBV5-6-3 | TRBJ2 | 9129 | 0.650692 |
| b | TRBV5-6-3 | TRBJ3 | 6843 | 0.487752 |
| b | TRBV5-6-3 | TRBJ4 | 4961 | 0.353607 |
| b | TRBV5-6-4 | TRBJ1 | 349 | 0.024876 |
| b | TRBV5-6-4 | TRBJ2 | 680 | 0.048469 |
| b | TRBV5-6-4 | TRBJ3 | 678 | 0.048326 |
| b | TRBV5-6-4 | TRBJ4 | 460 | 0.032788 |
| b | TRBV5-6-5 | TRBJ1 | 472 | 0.033643 |
| b | TRBV5-6-5 | TRBJ2 | 1065 | 0.07591 |
| b | TRBV5-6-5 | TRBJ3 | 912 | 0.065005 |
| b | TRBV5-6-5 | TRBJ4 | 1164 | 0.082967 |
| b | TRBV5-9 | TRBJ1 | 210 | 0.014968 |
| b | TRBV5-9 | TRBJ2 | 519 | 0.036993 |
| b | TRBV5-9 | TRBJ3 | 360 | 0.02566 |
| b | TRBV5-9 | TRBJ4 | 484 | 0.034498 |
| b | TRBV5-9-1 | TRBJ1 | 361 | 0.025731 |
| b | TRBV5-9-1 | TRBJ2 | 800 | 0.057022 |
| b | TRBV5-9-1 | TRBJ3 | 824 | 0.058733 |
| b | TRBV5-9-1 | TRBJ4 | 780 | 0.055596 |
| b | TRBV5-9-2 | TRBJ1 | 95 | 0.006771 |
| b | TRBV5-9-2 | TRBJ2 | 158 | 0.011262 |
| b | TRBV5-9-2 | TRBJ3 | 181 | 0.012901 |
| b | TRBV5-9-2 | TRBJ4 | 152 | 0.010834 |
| b | TRBV5-9-3 | TRBJ1 | 1355 | 0.096581 |
| b | TRBV5-9-3 | TRBJ2 | 3429 | 0.24441 |
| b | TRBV5-9-3 | TRBJ3 | 2120 | 0.151108 |
| b | TRBV5-9-3 | TRBJ4 | 2606 | 0.185749 |
| b | TRBV5-9-4 | TRBJ1 | 876 | 0.062439 |
| b | TRBV5-9-4 | TRBJ2 | 1466 | 0.104493 |
| b | TRBV5-9-4 | TRBJ3 | 1782 | 0.127016 |
| b | TRBV5-9-4 | TRBJ4 | 2044 | 0.145691 |
| b | TRBV5S21 | TRBJ1 | 1 | 7.13E-05 |
| b | TRBV5S21 | TRBJ2 | 6 | 0.000428 |
| b | TRBV5S21 | TRBJ3 | 3 | 0.000214 |
| b | TRBV5S21 | TRBJ4 | 2 | 0.000143 |
| b | TRBV5S4 | TRBJ2 | 2 | 0.000143 |
| b | TRBV5S4 | TRBJ3 | 2 | 0.000143 |
| b | TRBV5S4 | TRBJ4 | 2 | 0.000143 |
| b | TRBV5S9 | TRBJ1 | 384 | 0.027371 |
| b | TRBV5S9 | TRBJ2 | 1613 | 0.114971 |
| b | TRBV5S9 | TRBJ3 | 731 | 0.052104 |
| b | TRBV5S9 | TRBJ4 | 965 | 0.068783 |
| b | TRBV6-3 | TRBJ1 | 72 | 0.005132 |
| b | TRBV6-3 | TRBJ2 | 12 | 0.000855 |
| b | TRBV6-3 | TRBJ3 | 71 | 0.005061 |
| b | TRBV6-3 | TRBJ4 | 11 | 0.000784 |
| b | TRBV6-4 | TRBJ4 | 1 | 7.13E-05 |
| b | TRBV6-6 | TRBJ1 | 6087 | 0.433866 |
| b | TRBV6-6 | TRBJ2 | 7829 | 0.558031 |
| b | TRBV6-6 | TRBJ3 | 13883 | 0.989545 |
| b | TRBV6-6 | TRBJ4 | 4946 | 0.352538 |
| b | TRBV6-7 | TRBJ3 | 1 | 7.13E-05 |
| b | TRBV6-7 | TRBJ4 | 8 | 0.00057 |
| b | TRBV7-1 | TRBJ1 | 2294 | 0.163511 |
| b | TRBV7-1 | TRBJ2 | 1284 | 0.09152 |
| b | TRBV7-1 | TRBJ3 | 1621 | 0.115541 |
| b | TRBV7-1 | TRBJ4 | 1301 | 0.092732 |
| b | TRBV7-3 | TRBJ1 | 2583 | 0.18411 |
| b | TRBV7-3 | TRBJ2 | 2684 | 0.191309 |
| b | TRBV7-3 | TRBJ3 | 3624 | 0.25831 |
| b | TRBV7-3 | TRBJ4 | 2411 | 0.17185 |
| b | TRBV7-4 | TRBJ3 | 2 | 0.000143 |
| b | TRBV8-11 | TRBJ1 | 35 | 0.002495 |
| b | TRBV8-11 | TRBJ2 | 78 | 0.00556 |
| b | TRBV8-11 | TRBJ3 | 87 | 0.006201 |
| b | TRBV8-11 | TRBJ4 | 201 | 0.014327 |
| b | TRBV8-12 | TRBJ1 | 81 | 0.005773 |
| b | TRBV8-12 | TRBJ2 | 541 | 0.038561 |
| b | TRBV8-12 | TRBJ3 | 162 | 0.011547 |
| b | TRBV8-12 | TRBJ4 | 448 | 0.031932 |
| b | TRBV8-13 | TRBJ1 | 1 | 7.13E-05 |
| b | TRBV8-13 | TRBJ2 | 36 | 0.002566 |
| b | TRBV8-13 | TRBJ3 | 3 | 0.000214 |
| b | TRBV8-13 | TRBJ4 | 5 | 0.000356 |
| b | TRBV8-2 | TRBJ1 | 10 | 0.000713 |
| b | TRBV8-2 | TRBJ2 | 21 | 0.001497 |
| b | TRBV8-2 | TRBJ3 | 19 | 0.001354 |
| b | TRBV8-2 | TRBJ4 | 9 | 0.000641 |
| b | TRBV8-4 | TRBJ1 | 1 | 7.13E-05 |
| b | TRBV8-4 | TRBJ2 | 3 | 0.000214 |
| b | TRBV8-4 | TRBJ3 | 10 | 0.000713 |
| b | TRBV8-4 | TRBJ4 | 1 | 7.13E-05 |
| b | TRBV8-5 | TRBJ1 | 41 | 0.002922 |
| b | TRBV8-5 | TRBJ2 | 28 | 0.001996 |
| b | TRBV8-5 | TRBJ3 | 21 | 0.001497 |
| b | TRBV8-5 | TRBJ4 | 35 | 0.002495 |
| b | TRBV8-7 | TRBJ2 | 5 | 0.000356 |
| b | TRBV8-8 | TRBJ1 | 123 | 0.008767 |
| b | TRBV8-8 | TRBJ2 | 390 | 0.027798 |
| b | TRBV8-8 | TRBJ3 | 178 | 0.012687 |
| b | TRBV8-8 | TRBJ4 | 392 | 0.027941 |
| b | TRBV8-8-1 | TRBJ1 | 1 | 7.13E-05 |
| b | TRBV8-8-1 | TRBJ2 | 13 | 0.000927 |
| b | TRBV8-8-1 | TRBJ4 | 4 | 0.000285 |
| b | TRBV9-1 | TRBJ2 | 1 | 7.13E-05 |
| b | TRBV9-1 | TRBJ3 | 1 | 7.13E-05 |
| b | TRBV9-3-1 | TRBJ4 | 1 | 7.13E-05 |
| c | TRBV1-11 | TRBJ1 | 121 | 0.011377 |
| c | TRBV1-11 | TRBJ2 | 1184 | 0.111325 |
| c | TRBV1-11 | TRBJ3 | 206 | 0.019369 |
| c | TRBV1-11 | TRBJ4 | 276 | 0.025951 |
| c | TRBV1-12 | TRBJ1 | 129 | 0.012129 |
| c | TRBV1-12 | TRBJ2 | 90 | 0.008462 |
| c | TRBV1-12 | TRBJ3 | 64 | 0.006018 |
| c | TRBV1-12 | TRBJ4 | 85 | 0.007992 |
| c | TRBV1-13 | TRBJ1 | 1 | 9.40E-05 |
| c | TRBV1-13 | TRBJ2 | 4 | 0.000376 |
| c | TRBV1-13 | TRBJ3 | 5 | 0.00047 |
| c | TRBV1-14 | TRBJ1 | 3992 | 0.375346 |
| c | TRBV1-14 | TRBJ2 | 17021 | 1.600393 |
| c | TRBV1-14 | TRBJ3 | 9303 | 0.874711 |
| c | TRBV1-14 | TRBJ4 | 9911 | 0.931878 |
| c | TRBV1-15 | TRBJ1 | 2081 | 0.195665 |
| c | TRBV1-15 | TRBJ2 | 5258 | 0.494382 |
| c | TRBV1-15 | TRBJ3 | 5346 | 0.502656 |
| c | TRBV1-15 | TRBJ4 | 5450 | 0.512434 |
| c | TRBV1-18 | TRBJ1 | 1670 | 0.157021 |
| c | TRBV1-18 | TRBJ2 | 3929 | 0.369423 |
| c | TRBV1-18 | TRBJ3 | 3690 | 0.346951 |
| c | TRBV1-18 | TRBJ4 | 3702 | 0.348079 |
| c | TRBV1-19 | TRBJ1 | 250 | 0.023506 |
| c | TRBV1-19 | TRBJ2 | 879 | 0.082648 |
| c | TRBV1-19 | TRBJ3 | 559 | 0.05256 |
| c | TRBV1-19 | TRBJ4 | 509 | 0.047859 |
| c | TRBV1-20 | TRBJ1 | 711 | 0.066852 |
| c | TRBV1-20 | TRBJ2 | 1788 | 0.168116 |
| c | TRBV1-20 | TRBJ3 | 1493 | 0.140379 |
| c | TRBV1-20 | TRBJ4 | 1873 | 0.176108 |
| c | TRBV1-21 | TRBJ1 | 1 | 9.40E-05 |
| c | TRBV1-21 | TRBJ2 | 3 | 0.000282 |
| c | TRBV1-22 | TRBJ1 | 56 | 0.005265 |
| c | TRBV1-22 | TRBJ2 | 159 | 0.01495 |
| c | TRBV1-22 | TRBJ3 | 124 | 0.011659 |
| c | TRBV1-22 | TRBJ4 | 133 | 0.012505 |
| c | TRBV1-23 | TRBJ1 | 1 | 9.40E-05 |
| c | TRBV1-23 | TRBJ2 | 6 | 0.000564 |
| c | TRBV1-23 | TRBJ3 | 1 | 9.40E-05 |
| c | TRBV1-23 | TRBJ4 | 2 | 0.000188 |
| c | TRBV1-25 | TRBJ1 | 15 | 0.00141 |
| c | TRBV1-25 | TRBJ2 | 48 | 0.004513 |
| c | TRBV1-25 | TRBJ3 | 33 | 0.003103 |
| c | TRBV1-25 | TRBJ4 | 19 | 0.001786 |
| c | TRBV1-26 | TRBJ1 | 783 | 0.073621 |
| c | TRBV1-26 | TRBJ2 | 2188 | 0.205726 |
| c | TRBV1-26 | TRBJ3 | 1634 | 0.153636 |
| c | TRBV1-26 | TRBJ4 | 2593 | 0.243806 |
| c | TRBV1-28 | TRBJ1 | 3 | 0.000282 |
| c | TRBV1-28 | TRBJ2 | 4 | 0.000376 |
| c | TRBV1-28 | TRBJ3 | 11 | 0.001034 |
| c | TRBV1-28 | TRBJ4 | 7 | 0.000658 |
| c | TRBV1-31 | TRBJ1 | 148 | 0.013916 |
| c | TRBV1-31 | TRBJ2 | 433 | 0.040713 |
| c | TRBV1-31 | TRBJ3 | 308 | 0.02896 |
| c | TRBV1-31 | TRBJ4 | 448 | 0.042123 |
| c | TRBV1-34 | TRBJ1 | 479 | 0.045038 |
| c | TRBV1-34 | TRBJ2 | 1132 | 0.106436 |
| c | TRBV1-34 | TRBJ3 | 1047 | 0.098444 |
| c | TRBV1-34 | TRBJ4 | 875 | 0.082272 |
| c | TRBV1-36 | TRBJ1 | 14 | 0.001316 |
| c | TRBV1-36 | TRBJ2 | 36 | 0.003385 |
| c | TRBV1-36 | TRBJ3 | 31 | 0.002915 |
| c | TRBV1-36 | TRBJ4 | 23 | 0.002163 |
| c | TRBV1-37 | TRBJ1 | 637 | 0.059894 |
| c | TRBV1-37 | TRBJ2 | 1893 | 0.177989 |
| c | TRBV1-37 | TRBJ3 | 1103 | 0.103709 |
| c | TRBV1-37 | TRBJ4 | 1773 | 0.166706 |
| c | TRBV1-39 | TRBJ1 | 752 | 0.070707 |
| c | TRBV1-39 | TRBJ2 | 2347 | 0.220676 |
| c | TRBV1-39 | TRBJ3 | 1420 | 0.133515 |
| c | TRBV1-39 | TRBJ4 | 1715 | 0.161252 |
| c | TRBV1-4 | TRBJ1 | 1802 | 0.169432 |
| c | TRBV1-4 | TRBJ2 | 5703 | 0.536223 |
| c | TRBV1-4 | TRBJ3 | 4349 | 0.408913 |
| c | TRBV1-4 | TRBJ4 | 6451 | 0.606553 |
| c | TRBV1-42 | TRBJ1 | 229 | 0.021532 |
| c | TRBV1-42 | TRBJ2 | 751 | 0.070613 |
| c | TRBV1-42 | TRBJ3 | 712 | 0.066946 |
| c | TRBV1-42 | TRBJ4 | 509 | 0.047859 |
| c | TRBV1-43 | TRBJ1 | 8 | 0.000752 |
| c | TRBV1-43 | TRBJ2 | 46 | 0.004325 |
| c | TRBV1-43 | TRBJ3 | 16 | 0.001504 |
| c | TRBV1-43 | TRBJ4 | 27 | 0.002539 |
| c | TRBV1-47 | TRBJ1 | 307 | 0.028866 |
| c | TRBV1-47 | TRBJ2 | 1109 | 0.104273 |
| c | TRBV1-47 | TRBJ3 | 4846 | 0.455643 |
| c | TRBV1-47 | TRBJ4 | 869 | 0.081707 |
| c | TRBV1-48 | TRBJ4 | 1 | 9.40E-05 |
| c | TRBV1-49 | TRBJ2 | 5 | 0.00047 |
| c | TRBV1-49 | TRBJ3 | 12 | 0.001128 |
| c | TRBV1-49 | TRBJ4 | 4 | 0.000376 |
| c | TRBV1-5 | TRBJ1 | 1453 | 0.136618 |
| c | TRBV1-5 | TRBJ2 | 3290 | 0.309341 |
| c | TRBV1-5 | TRBJ3 | 5022 | 0.472192 |
| c | TRBV1-5 | TRBJ4 | 3173 | 0.29834 |
| c | TRBV1-50 | TRBJ1 | 58 | 0.005453 |
| c | TRBV1-50 | TRBJ2 | 71 | 0.006676 |
| c | TRBV1-50 | TRBJ3 | 67 | 0.0063 |
| c | TRBV1-50 | TRBJ4 | 60 | 0.005641 |
| c | TRBV1-52 | TRBJ1 | 240 | 0.022566 |
| c | TRBV1-52 | TRBJ2 | 623 | 0.058577 |
| c | TRBV1-52 | TRBJ3 | 610 | 0.057355 |
| c | TRBV1-52 | TRBJ4 | 322 | 0.030276 |
| c | TRBV1-53 | TRBJ1 | 682 | 0.064125 |
| c | TRBV1-53 | TRBJ2 | 1814 | 0.170561 |
| c | TRBV1-53 | TRBJ3 | 1291 | 0.121386 |
| c | TRBV1-53 | TRBJ4 | 1335 | 0.125523 |
| c | TRBV1-54 | TRBJ1 | 1577 | 0.148277 |
| c | TRBV1-54 | TRBJ2 | 4297 | 0.404024 |
| c | TRBV1-54 | TRBJ3 | 3152 | 0.296366 |
| c | TRBV1-54 | TRBJ4 | 3127 | 0.294015 |
| c | TRBV1-55 | TRBJ1 | 650 | 0.061116 |
| c | TRBV1-55 | TRBJ2 | 1126 | 0.105872 |
| c | TRBV1-55 | TRBJ3 | 903 | 0.084904 |
| c | TRBV1-55 | TRBJ4 | 829 | 0.077946 |
| c | TRBV1-56 | TRBJ1 | 25 | 0.002351 |
| c | TRBV1-56 | TRBJ2 | 80 | 0.007522 |
| c | TRBV1-56 | TRBJ3 | 41 | 0.003855 |
| c | TRBV1-56 | TRBJ4 | 51 | 0.004795 |
| c | TRBV1-58 | TRBJ1 | 2 | 0.000188 |
| c | TRBV1-58 | TRBJ2 | 4 | 0.000376 |
| c | TRBV1-58 | TRBJ3 | 3 | 0.000282 |
| c | TRBV1-58 | TRBJ4 | 1 | 9.40E-05 |
| c | TRBV1-59 | TRBJ1 | 47 | 0.004419 |
| c | TRBV1-59 | TRBJ2 | 167 | 0.015702 |
| c | TRBV1-59 | TRBJ3 | 79 | 0.007428 |
| c | TRBV1-59 | TRBJ4 | 86 | 0.008086 |
| c | TRBV1-61 | TRBJ1 | 203 | 0.019087 |
| c | TRBV1-61 | TRBJ2 | 958 | 0.090076 |
| c | TRBV1-61 | TRBJ3 | 840 | 0.078981 |
| c | TRBV1-61 | TRBJ4 | 613 | 0.057637 |
| c | TRBV1-62-1 | TRBJ2 | 1 | 9.40E-05 |
| c | TRBV1-62-2 | TRBJ1 | 661 | 0.06215 |
| c | TRBV1-62-2 | TRBJ2 | 1104 | 0.103803 |
| c | TRBV1-62-2 | TRBJ3 | 1156 | 0.108692 |
| c | TRBV1-62-2 | TRBJ4 | 1103 | 0.103709 |
| c | TRBV1-62-3 | TRBJ1 | 14 | 0.001316 |
| c | TRBV1-62-3 | TRBJ2 | 37 | 0.003479 |
| c | TRBV1-62-3 | TRBJ3 | 18 | 0.001692 |
| c | TRBV1-62-3 | TRBJ4 | 17 | 0.001598 |
| c | TRBV1-63 | TRBJ1 | 2417 | 0.227258 |
| c | TRBV1-63 | TRBJ2 | 2253 | 0.211838 |
| c | TRBV1-63 | TRBJ3 | 1722 | 0.16191 |
| c | TRBV1-63 | TRBJ4 | 1674 | 0.157397 |
| c | TRBV1-64 | TRBJ1 | 62 | 0.00583 |
| c | TRBV1-64 | TRBJ2 | 190 | 0.017865 |
| c | TRBV1-64 | TRBJ3 | 139 | 0.013069 |
| c | TRBV1-64 | TRBJ4 | 115 | 0.010813 |
| c | TRBV1-66 | TRBJ1 | 211 | 0.019839 |
| c | TRBV1-66 | TRBJ2 | 418 | 0.039302 |
| c | TRBV1-66 | TRBJ3 | 285 | 0.026797 |
| c | TRBV1-66 | TRBJ4 | 336 | 0.031592 |
| c | TRBV1-67 | TRBJ1 | 1182 | 0.111137 |
| c | TRBV1-67 | TRBJ2 | 2809 | 0.264115 |
| c | TRBV1-67 | TRBJ3 | 1585 | 0.149029 |
| c | TRBV1-67 | TRBJ4 | 4446 | 0.418034 |
| c | TRBV1-69 | TRBJ1 | 730 | 0.068638 |
| c | TRBV1-69 | TRBJ2 | 2319 | 0.218043 |
| c | TRBV1-69 | TRBJ3 | 2113 | 0.198674 |
| c | TRBV1-69 | TRBJ4 | 1771 | 0.166518 |
| c | TRBV1-7 | TRBJ1 | 5817 | 0.546941 |
| c | TRBV1-7 | TRBJ2 | 18280 | 1.71877 |
| c | TRBV1-7 | TRBJ3 | 10574 | 0.994217 |
| c | TRBV1-7 | TRBJ4 | 12540 | 1.179069 |
| c | TRBV1-70 | TRBJ1 | 3 | 0.000282 |
| c | TRBV1-70 | TRBJ2 | 28 | 0.002633 |
| c | TRBV1-70 | TRBJ3 | 20 | 0.00188 |
| c | TRBV1-70 | TRBJ4 | 16 | 0.001504 |
| c | TRBV1-72 | TRBJ1 | 3 | 0.000282 |
| c | TRBV1-72 | TRBJ3 | 2 | 0.000188 |
| c | TRBV1-74 | TRBJ1 | 1127 | 0.105966 |
| c | TRBV1-74 | TRBJ2 | 2948 | 0.277185 |
| c | TRBV1-74 | TRBJ3 | 2064 | 0.194067 |
| c | TRBV1-74 | TRBJ4 | 1804 | 0.16962 |
| c | TRBV1-75 | TRBJ1 | 2 | 0.000188 |
| c | TRBV1-75 | TRBJ2 | 14 | 0.001316 |
| c | TRBV1-75 | TRBJ3 | 6 | 0.000564 |
| c | TRBV1-75 | TRBJ4 | 8 | 0.000752 |
| c | TRBV1-76 | TRBJ1 | 105 | 0.009873 |
| c | TRBV1-76 | TRBJ2 | 268 | 0.025199 |
| c | TRBV1-76 | TRBJ3 | 216 | 0.020309 |
| c | TRBV1-76 | TRBJ4 | 241 | 0.02266 |
| c | TRBV1-77 | TRBJ1 | 847 | 0.079639 |
| c | TRBV1-77 | TRBJ2 | 1826 | 0.171689 |
| c | TRBV1-77 | TRBJ3 | 2343 | 0.2203 |
| c | TRBV1-77 | TRBJ4 | 1347 | 0.126651 |
| c | TRBV1-78 | TRBJ1 | 15 | 0.00141 |
| c | TRBV1-78 | TRBJ2 | 74 | 0.006958 |
| c | TRBV1-78 | TRBJ3 | 40 | 0.003761 |
| c | TRBV1-78 | TRBJ4 | 62 | 0.00583 |
| c | TRBV1-8 | TRBJ3 | 2 | 0.000188 |
| c | TRBV1-8 | TRBJ4 | 2 | 0.000188 |
| c | TRBV1-80 | TRBJ1 | 2190 | 0.205914 |
| c | TRBV1-80 | TRBJ2 | 5936 | 0.55813 |
| c | TRBV1-80 | TRBJ3 | 2559 | 0.240609 |
| c | TRBV1-80 | TRBJ4 | 5872 | 0.552113 |
| c | TRBV1-81 | TRBJ1 | 337 | 0.031686 |
| c | TRBV1-81 | TRBJ2 | 1164 | 0.109445 |
| c | TRBV1-81 | TRBJ3 | 783 | 0.073621 |
| c | TRBV1-81 | TRBJ4 | 1055 | 0.099196 |
| c | TRBV1-82 | TRBJ1 | 1885 | 0.177236 |
| c | TRBV1-82 | TRBJ2 | 4275 | 0.401955 |
| c | TRBV1-82 | TRBJ3 | 3407 | 0.320342 |
| c | TRBV1-82 | TRBJ4 | 4034 | 0.379295 |
| c | TRBV1-83 | TRBJ1 | 252 | 0.023694 |
| c | TRBV1-83 | TRBJ2 | 598 | 0.056227 |
| c | TRBV1-83 | TRBJ3 | 370 | 0.034789 |
| c | TRBV1-83 | TRBJ4 | 395 | 0.03714 |
| c | TRBV1-84 | TRBJ1 | 578 | 0.054346 |
| c | TRBV1-84 | TRBJ2 | 1452 | 0.136524 |
| c | TRBV1-84 | TRBJ3 | 1329 | 0.124959 |
| c | TRBV1-84 | TRBJ4 | 961 | 0.090358 |
| c | TRBV1-85 | TRBJ1 | 353 | 0.033191 |
| c | TRBV1-85 | TRBJ2 | 1032 | 0.097033 |
| c | TRBV1-85 | TRBJ3 | 615 | 0.057825 |
| c | TRBV1-85 | TRBJ4 | 720 | 0.067698 |
| c | TRBV1-87 | TRBJ1 | 1580 | 0.148559 |
| c | TRBV1-87 | TRBJ2 | 5738 | 0.539513 |
| c | TRBV1-87 | TRBJ3 | 2887 | 0.271449 |
| c | TRBV1-87 | TRBJ4 | 4206 | 0.395468 |
| c | TRBV1-9 | TRBJ1 | 5590 | 0.525598 |
| c | TRBV1-9 | TRBJ2 | 20278 | 1.906632 |
| c | TRBV1-9 | TRBJ3 | 13071 | 1.228996 |
| c | TRBV1-9 | TRBJ4 | 12769 | 1.200601 |
| c | TRBV10-1 | TRBJ1 | 1138 | 0.107 |
| c | TRBV10-1 | TRBJ2 | 2848 | 0.267782 |
| c | TRBV10-1 | TRBJ3 | 3806 | 0.357858 |
| c | TRBV10-1 | TRBJ4 | 3971 | 0.373372 |
| c | TRBV10-3 | TRBJ1 | 73 | 0.006864 |
| c | TRBV10-3 | TRBJ2 | 118 | 0.011095 |
| c | TRBV10-3 | TRBJ3 | 336 | 0.031592 |
| c | TRBV10-3 | TRBJ4 | 453 | 0.042593 |
| c | TRBV10S3 | TRBJ1 | 45 | 0.004231 |
| c | TRBV10S3 | TRBJ2 | 103 | 0.009685 |
| c | TRBV10S3 | TRBJ3 | 179 | 0.01683 |
| c | TRBV10S3 | TRBJ4 | 95 | 0.008932 |
| c | TRBV11-1 | TRBJ1 | 2 | 0.000188 |
| c | TRBV11-2 | TRBJ1 | 5 | 0.00047 |
| c | TRBV12-3 | TRBJ1 | 100 | 0.009402 |
| c | TRBV12-3 | TRBJ2 | 15 | 0.00141 |
| c | TRBV12-3 | TRBJ3 | 41 | 0.003855 |
| c | TRBV12-3 | TRBJ4 | 5 | 0.00047 |
| c | TRBV13-2 | TRBJ1 | 2 | 0.000188 |
| c | TRBV13-2 | TRBJ2 | 3 | 0.000282 |
| c | TRBV13-2 | TRBJ3 | 5 | 0.00047 |
| c | TRBV14-1 | TRBJ1 | 3426 | 0.322128 |
| c | TRBV14-1 | TRBJ2 | 5404 | 0.508109 |
| c | TRBV14-1 | TRBJ3 | 5188 | 0.4878 |
| c | TRBV14-1 | TRBJ4 | 2691 | 0.25302 |
| c | TRBV14-2 | TRBJ1 | 970 | 0.091204 |
| c | TRBV14-2 | TRBJ2 | 2161 | 0.203187 |
| c | TRBV14-2 | TRBJ3 | 1604 | 0.150816 |
| c | TRBV14-2 | TRBJ4 | 1167 | 0.109727 |
| c | TRBV14-3 | TRBJ1 | 62407 | 5.867796 |
| c | TRBV14-3 | TRBJ2 | 39002 | 3.667149 |
| c | TRBV14-3 | TRBJ3 | 23749 | 2.232991 |
| c | TRBV14-3 | TRBJ4 | 26415 | 2.483661 |
| c | TRBV14-4 | TRBJ1 | 2835 | 0.26656 |
| c | TRBV14-4 | TRBJ2 | 6586 | 0.619246 |
| c | TRBV14-4 | TRBJ3 | 5951 | 0.559541 |
| c | TRBV14-4 | TRBJ4 | 4456 | 0.418974 |
| c | TRBV15-2 | TRBJ3 | 1 | 9.40E-05 |
| c | TRBV15-2 | TRBJ4 | 4 | 0.000376 |
| c | TRBV1S10 | TRBJ1 | 2 | 0.000188 |
| c | TRBV1S10 | TRBJ2 | 12 | 0.001128 |
| c | TRBV1S10 | TRBJ3 | 5 | 0.00047 |
| c | TRBV1S10 | TRBJ4 | 11 | 0.001034 |
| c | TRBV1S11 | TRBJ2 | 1 | 9.40E-05 |
| c | TRBV1S11 | TRBJ4 | 1 | 9.40E-05 |
| c | TRBV1S12 | TRBJ1 | 27 | 0.002539 |
| c | TRBV1S12 | TRBJ2 | 65 | 0.006112 |
| c | TRBV1S12 | TRBJ3 | 52 | 0.004889 |
| c | TRBV1S12 | TRBJ4 | 48 | 0.004513 |
| c | TRBV1S126 | TRBJ1 | 122 | 0.011471 |
| c | TRBV1S126 | TRBJ2 | 893 | 0.083964 |
| c | TRBV1S126 | TRBJ3 | 264 | 0.024823 |
| c | TRBV1S126 | TRBJ4 | 255 | 0.023976 |
| c | TRBV1S127 | TRBJ1 | 404 | 0.037986 |
| c | TRBV1S127 | TRBJ2 | 1021 | 0.095999 |
| c | TRBV1S127 | TRBJ3 | 609 | 0.057261 |
| c | TRBV1S127 | TRBJ4 | 647 | 0.060834 |
| c | TRBV1S130 | TRBJ1 | 722 | 0.067886 |
| c | TRBV1S130 | TRBJ2 | 1370 | 0.128814 |
| c | TRBV1S130 | TRBJ3 | 1184 | 0.111325 |
| c | TRBV1S130 | TRBJ4 | 1530 | 0.143858 |
| c | TRBV1S132 | TRBJ1 | 190 | 0.017865 |
| c | TRBV1S132 | TRBJ2 | 538 | 0.050585 |
| c | TRBV1S132 | TRBJ3 | 335 | 0.031498 |
| c | TRBV1S132 | TRBJ4 | 400 | 0.03761 |
| c | TRBV1S134 | TRBJ1 | 7 | 0.000658 |
| c | TRBV1S134 | TRBJ2 | 11 | 0.001034 |
| c | TRBV1S134 | TRBJ3 | 11 | 0.001034 |
| c | TRBV1S134 | TRBJ4 | 17 | 0.001598 |
| c | TRBV1S135 | TRBJ1 | 807 | 0.075878 |
| c | TRBV1S135 | TRBJ2 | 2731 | 0.256781 |
| c | TRBV1S135 | TRBJ3 | 2400 | 0.225659 |
| c | TRBV1S135 | TRBJ4 | 2216 | 0.208359 |
| c | TRBV1S136 | TRBJ1 | 28 | 0.002633 |
| c | TRBV1S136 | TRBJ2 | 177 | 0.016642 |
| c | TRBV1S136 | TRBJ3 | 92 | 0.00865 |
| c | TRBV1S136 | TRBJ4 | 144 | 0.01354 |
| c | TRBV1S137 | TRBJ1 | 519 | 0.048799 |
| c | TRBV1S137 | TRBJ2 | 1549 | 0.145644 |
| c | TRBV1S137 | TRBJ3 | 1283 | 0.120634 |
| c | TRBV1S137 | TRBJ4 | 1948 | 0.18316 |
| c | TRBV1S14 | TRBJ1 | 16 | 0.001504 |
| c | TRBV1S14 | TRBJ2 | 23 | 0.002163 |
| c | TRBV1S14 | TRBJ3 | 19 | 0.001786 |
| c | TRBV1S14 | TRBJ4 | 23 | 0.002163 |
| c | TRBV1S15 | TRBJ2 | 8 | 0.000752 |
| c | TRBV1S15 | TRBJ3 | 1 | 9.40E-05 |
| c | TRBV1S16 | TRBJ1 | 643 | 0.060458 |
| c | TRBV1S16 | TRBJ2 | 1533 | 0.14414 |
| c | TRBV1S16 | TRBJ3 | 1478 | 0.138968 |
| c | TRBV1S16 | TRBJ4 | 1092 | 0.102675 |
| c | TRBV1S17 | TRBJ1 | 10 | 0.00094 |
| c | TRBV1S17 | TRBJ2 | 36 | 0.003385 |
| c | TRBV1S17 | TRBJ3 | 39 | 0.003667 |
| c | TRBV1S17 | TRBJ4 | 22 | 0.002069 |
| c | TRBV1S18 | TRBJ2 | 2 | 0.000188 |
| c | TRBV1S18 | TRBJ4 | 3 | 0.000282 |
| c | TRBV1S19 | TRBJ1 | 4 | 0.000376 |
| c | TRBV1S19 | TRBJ2 | 13 | 0.001222 |
| c | TRBV1S19 | TRBJ3 | 5 | 0.00047 |
| c | TRBV1S19 | TRBJ4 | 8 | 0.000752 |
| c | TRBV1S22 | TRBJ1 | 737 | 0.069296 |
| c | TRBV1S22 | TRBJ2 | 1733 | 0.162945 |
| c | TRBV1S22 | TRBJ3 | 1644 | 0.154577 |
| c | TRBV1S22 | TRBJ4 | 1370 | 0.128814 |
| c | TRBV1S26 | TRBJ1 | 593 | 0.055757 |
| c | TRBV1S26 | TRBJ2 | 1763 | 0.165765 |
| c | TRBV1S26 | TRBJ3 | 1119 | 0.105214 |
| c | TRBV1S26 | TRBJ4 | 1623 | 0.152602 |
| c | TRBV1S28 | TRBJ1 | 1 | 9.40E-05 |
| c | TRBV1S28 | TRBJ2 | 3 | 0.000282 |
| c | TRBV1S28 | TRBJ3 | 5 | 0.00047 |
| c | TRBV1S28 | TRBJ4 | 7 | 0.000658 |
| c | TRBV1S29 | TRBJ1 | 1114 | 0.104743 |
| c | TRBV1S29 | TRBJ2 | 2363 | 0.22218 |
| c | TRBV1S29 | TRBJ3 | 2052 | 0.192939 |
| c | TRBV1S29 | TRBJ4 | 2087 | 0.196229 |
| c | TRBV1S30 | TRBJ1 | 32 | 0.003009 |
| c | TRBV1S30 | TRBJ2 | 82 | 0.00771 |
| c | TRBV1S30 | TRBJ3 | 75 | 0.007052 |
| c | TRBV1S30 | TRBJ4 | 104 | 0.009779 |
| c | TRBV1S34 | TRBJ1 | 918 | 0.086315 |
| c | TRBV1S34 | TRBJ2 | 1604 | 0.150816 |
| c | TRBV1S34 | TRBJ3 | 1378 | 0.129566 |
| c | TRBV1S34 | TRBJ4 | 2153 | 0.202435 |
| c | TRBV1S36 | TRBJ1 | 11 | 0.001034 |
| c | TRBV1S36 | TRBJ2 | 22 | 0.002069 |
| c | TRBV1S36 | TRBJ3 | 13 | 0.001222 |
| c | TRBV1S36 | TRBJ4 | 19 | 0.001786 |
| c | TRBV1S40 | TRBJ1 | 60 | 0.005641 |
| c | TRBV1S40 | TRBJ2 | 77 | 0.00724 |
| c | TRBV1S40 | TRBJ3 | 26 | 0.002445 |
| c | TRBV1S40 | TRBJ4 | 37 | 0.003479 |
| c | TRBV1S41 | TRBJ1 | 893 | 0.083964 |
| c | TRBV1S41 | TRBJ2 | 4005 | 0.376569 |
| c | TRBV1S41 | TRBJ3 | 1338 | 0.125805 |
| c | TRBV1S41 | TRBJ4 | 1623 | 0.152602 |
| c | TRBV1S45 | TRBJ1 | 271 | 0.025481 |
| c | TRBV1S45 | TRBJ2 | 651 | 0.06121 |
| c | TRBV1S45 | TRBJ3 | 400 | 0.03761 |
| c | TRBV1S45 | TRBJ4 | 2088 | 0.196323 |
| c | TRBV1S46 | TRBJ1 | 25 | 0.002351 |
| c | TRBV1S46 | TRBJ2 | 46 | 0.004325 |
| c | TRBV1S46 | TRBJ3 | 24 | 0.002257 |
| c | TRBV1S46 | TRBJ4 | 34 | 0.003197 |
| c | TRBV1S5 | TRBJ1 | 90 | 0.008462 |
| c | TRBV1S5 | TRBJ2 | 137 | 0.012881 |
| c | TRBV1S5 | TRBJ3 | 147 | 0.013822 |
| c | TRBV1S5 | TRBJ4 | 107 | 0.010061 |
| c | TRBV1S50 | TRBJ1 | 7 | 0.000658 |
| c | TRBV1S50 | TRBJ2 | 15 | 0.00141 |
| c | TRBV1S50 | TRBJ3 | 6 | 0.000564 |
| c | TRBV1S50 | TRBJ4 | 12 | 0.001128 |
| c | TRBV1S52 | TRBJ2 | 2 | 0.000188 |
| c | TRBV1S52 | TRBJ4 | 1 | 9.40E-05 |
| c | TRBV1S53 | TRBJ1 | 789 | 0.074185 |
| c | TRBV1S53 | TRBJ2 | 3479 | 0.327112 |
| c | TRBV1S53 | TRBJ3 | 1739 | 0.163509 |
| c | TRBV1S53 | TRBJ4 | 1343 | 0.126275 |
| c | TRBV1S55 | TRBJ1 | 25 | 0.002351 |
| c | TRBV1S55 | TRBJ2 | 41 | 0.003855 |
| c | TRBV1S55 | TRBJ3 | 54 | 0.005077 |
| c | TRBV1S55 | TRBJ4 | 41 | 0.003855 |
| c | TRBV1S56 | TRBJ1 | 1043 | 0.098068 |
| c | TRBV1S56 | TRBJ2 | 2198 | 0.206666 |
| c | TRBV1S56 | TRBJ3 | 1651 | 0.155235 |
| c | TRBV1S56 | TRBJ4 | 3124 | 0.293733 |
| c | TRBV1S61 | TRBJ1 | 2008 | 0.188801 |
| c | TRBV1S61 | TRBJ2 | 2245 | 0.211085 |
| c | TRBV1S61 | TRBJ3 | 2293 | 0.215598 |
| c | TRBV1S61 | TRBJ4 | 1729 | 0.162569 |
| c | TRBV1S72 | TRBJ2 | 2 | 0.000188 |
| c | TRBV1S74 | TRBJ1 | 1 | 9.40E-05 |
| c | TRBV1S81 | TRBJ1 | 2149 | 0.202059 |
| c | TRBV1S81 | TRBJ2 | 8537 | 0.802688 |
| c | TRBV1S81 | TRBJ3 | 7636 | 0.717972 |
| c | TRBV1S81 | TRBJ4 | 6708 | 0.630717 |
| c | TRBV1S82 | TRBJ1 | 58 | 0.005453 |
| c | TRBV1S82 | TRBJ2 | 126 | 0.011847 |
| c | TRBV1S82 | TRBJ3 | 96 | 0.009026 |
| c | TRBV1S82 | TRBJ4 | 99 | 0.009308 |
| c | TRBV1S9 | TRBJ1 | 4 | 0.000376 |
| c | TRBV1S9 | TRBJ2 | 9 | 0.000846 |
| c | TRBV1S9 | TRBJ3 | 4 | 0.000376 |
| c | TRBV1S9 | TRBJ4 | 12 | 0.001128 |
| c | TRBV2-2 | TRBJ1 | 1362 | 0.128062 |
| c | TRBV2-2 | TRBJ2 | 5395 | 0.507263 |
| c | TRBV2-2 | TRBJ3 | 3691 | 0.347045 |
| c | TRBV2-2 | TRBJ4 | 6492 | 0.610408 |
| c | TRBV2-2-1 | TRBJ2 | 9 | 0.000846 |
| c | TRBV2-2-1 | TRBJ3 | 6 | 0.000564 |
| c | TRBV2-2-1 | TRBJ4 | 10 | 0.00094 |
| c | TRBV2-2-2 | TRBJ1 | 6 | 0.000564 |
| c | TRBV2-2-2 | TRBJ2 | 32 | 0.003009 |
| c | TRBV2-2-2 | TRBJ3 | 35 | 0.003291 |
| c | TRBV2-2-2 | TRBJ4 | 20 | 0.00188 |
| c | TRBV2-3 | TRBJ1 | 1527 | 0.143576 |
| c | TRBV2-3 | TRBJ2 | 1750 | 0.164543 |
| c | TRBV2-3 | TRBJ3 | 3088 | 0.290348 |
| c | TRBV2-3 | TRBJ4 | 4775 | 0.448968 |
| c | TRBV2-3-1 | TRBJ1 | 2 | 0.000188 |
| c | TRBV2-3-1 | TRBJ2 | 5 | 0.00047 |
| c | TRBV2-3-1 | TRBJ3 | 3 | 0.000282 |
| c | TRBV2-3-1 | TRBJ4 | 7 | 0.000658 |
| c | TRBV2-4 | TRBJ1 | 179 | 0.01683 |
| c | TRBV2-4 | TRBJ2 | 682 | 0.064125 |
| c | TRBV2-4 | TRBJ3 | 636 | 0.0598 |
| c | TRBV2-4 | TRBJ4 | 775 | 0.072869 |
| c | TRBV2-4-1 | TRBJ1 | 793 | 0.074562 |
| c | TRBV2-4-1 | TRBJ2 | 1543 | 0.14508 |
| c | TRBV2-4-1 | TRBJ3 | 1806 | 0.169809 |
| c | TRBV2-4-1 | TRBJ4 | 2213 | 0.208077 |
| c | TRBV2-5 | TRBJ1 | 623 | 0.058577 |
| c | TRBV2-5 | TRBJ2 | 1043 | 0.098068 |
| c | TRBV2-5 | TRBJ3 | 1437 | 0.135113 |
| c | TRBV2-5 | TRBJ4 | 4169 | 0.391989 |
| c | TRBV2-5-1 | TRBJ1 | 102 | 0.009591 |
| c | TRBV2-5-1 | TRBJ2 | 273 | 0.025669 |
| c | TRBV2-5-1 | TRBJ3 | 361 | 0.033943 |
| c | TRBV2-5-1 | TRBJ4 | 752 | 0.070707 |
| c | TRBV2-6 | TRBJ1 | 900 | 0.084622 |
| c | TRBV2-6 | TRBJ2 | 1960 | 0.184288 |
| c | TRBV2-6 | TRBJ3 | 3304 | 0.310657 |
| c | TRBV2-6 | TRBJ4 | 3615 | 0.339899 |
| c | TRBV2-6-1 | TRBJ1 | 585 | 0.055004 |
| c | TRBV2-6-1 | TRBJ2 | 475 | 0.044662 |
| c | TRBV2-6-1 | TRBJ3 | 752 | 0.070707 |
| c | TRBV2-6-1 | TRBJ4 | 1688 | 0.158714 |
| c | TRBV2-6-2 | TRBJ1 | 230 | 0.021626 |
| c | TRBV2-6-2 | TRBJ2 | 236 | 0.02219 |
| c | TRBV2-6-2 | TRBJ3 | 257 | 0.024164 |
| c | TRBV2-6-2 | TRBJ4 | 793 | 0.074562 |
| c | TRBV2-6-4 | TRBJ1 | 193 | 0.018147 |
| c | TRBV2-6-4 | TRBJ2 | 352 | 0.033097 |
| c | TRBV2-6-4 | TRBJ3 | 535 | 0.050303 |
| c | TRBV2-6-4 | TRBJ4 | 709 | 0.066663 |
| c | TRBV2-6-5 | TRBJ1 | 344 | 0.032344 |
| c | TRBV2-6-5 | TRBJ2 | 1144 | 0.107564 |
| c | TRBV2-6-5 | TRBJ3 | 918 | 0.086315 |
| c | TRBV2-6-5 | TRBJ4 | 780 | 0.073339 |
| c | TRBV2-6-6 | TRBJ1 | 6 | 0.000564 |
| c | TRBV2-6-6 | TRBJ2 | 25 | 0.002351 |
| c | TRBV2-6-6 | TRBJ3 | 20 | 0.00188 |
| c | TRBV2-6-6 | TRBJ4 | 25 | 0.002351 |
| c | TRBV2-6-7 | TRBJ1 | 1735 | 0.163133 |
| c | TRBV2-6-7 | TRBJ2 | 2244 | 0.210991 |
| c | TRBV2-6-7 | TRBJ3 | 6868 | 0.645761 |
| c | TRBV2-6-7 | TRBJ4 | 3889 | 0.365662 |
| c | TRBV2-7 | TRBJ1 | 3 | 0.000282 |
| c | TRBV2-7 | TRBJ2 | 1 | 9.40E-05 |
| c | TRBV2-9 | TRBJ1 | 5662 | 0.532368 |
| c | TRBV2-9 | TRBJ2 | 11558 | 1.086737 |
| c | TRBV2-9 | TRBJ3 | 18691 | 1.757415 |
| c | TRBV2-9 | TRBJ4 | 17943 | 1.687084 |
| c | TRBV2-9-1 | TRBJ1 | 296 | 0.027831 |
| c | TRBV2-9-1 | TRBJ2 | 580 | 0.054534 |
| c | TRBV2-9-1 | TRBJ3 | 650 | 0.061116 |
| c | TRBV2-9-1 | TRBJ4 | 737 | 0.069296 |
| c | TRBV2-9-2 | TRBJ1 | 156 | 0.014668 |
| c | TRBV2-9-2 | TRBJ2 | 336 | 0.031592 |
| c | TRBV2-9-2 | TRBJ3 | 389 | 0.036576 |
| c | TRBV2-9-2 | TRBJ4 | 373 | 0.035071 |
| c | TRBV2S3 | TRBJ1 | 214 | 0.020121 |
| c | TRBV2S3 | TRBJ2 | 87 | 0.00818 |
| c | TRBV2S3 | TRBJ3 | 203 | 0.019087 |
| c | TRBV2S3 | TRBJ4 | 143 | 0.013446 |
| c | TRBV3-1 | TRBJ1 | 2180 | 0.204974 |
| c | TRBV3-1 | TRBJ2 | 2949 | 0.277279 |
| c | TRBV3-1 | TRBJ3 | 2346 | 0.220582 |
| c | TRBV3-1 | TRBJ4 | 2711 | 0.254901 |
| c | TRBV3-2 | TRBJ1 | 5660 | 0.532179 |
| c | TRBV3-2 | TRBJ2 | 17759 | 1.669784 |
| c | TRBV3-2 | TRBJ3 | 8254 | 0.776079 |
| c | TRBV3-2 | TRBJ4 | 9376 | 0.881575 |
| c | TRBV3-3 | TRBJ1 | 41 | 0.003855 |
| c | TRBV3-3 | TRBJ2 | 8 | 0.000752 |
| c | TRBV3-3 | TRBJ3 | 14 | 0.001316 |
| c | TRBV3-3 | TRBJ4 | 92 | 0.00865 |
| c | TRBV3-4 | TRBJ1 | 480 | 0.045132 |
| c | TRBV3-4 | TRBJ2 | 2241 | 0.210709 |
| c | TRBV3-4 | TRBJ3 | 474 | 0.044568 |
| c | TRBV3-4 | TRBJ4 | 361 | 0.033943 |
| c | TRBV3-5 | TRBJ1 | 156 | 0.014668 |
| c | TRBV3-5 | TRBJ2 | 454 | 0.042687 |
| c | TRBV3-5 | TRBJ3 | 93 | 0.008744 |
| c | TRBV3-5 | TRBJ4 | 258 | 0.024258 |
| c | TRBV3-6 | TRBJ1 | 2255 | 0.212026 |
| c | TRBV3-6 | TRBJ2 | 5162 | 0.485355 |
| c | TRBV3-6 | TRBJ3 | 2784 | 0.261765 |
| c | TRBV3-6 | TRBJ4 | 2787 | 0.262047 |
| c | TRBV3-8 | TRBJ1 | 4696 | 0.44154 |
| c | TRBV3-8 | TRBJ2 | 3724 | 0.350148 |
| c | TRBV3-8 | TRBJ3 | 2595 | 0.243994 |
| c | TRBV3-8 | TRBJ4 | 5576 | 0.524281 |
| c | TRBV3S1 | TRBJ1 | 1 | 9.40E-05 |
| c | TRBV4-1 | TRBJ1 | 428 | 0.040243 |
| c | TRBV4-1 | TRBJ2 | 1104 | 0.103803 |
| c | TRBV4-1 | TRBJ3 | 997 | 0.093743 |
| c | TRBV4-1 | TRBJ4 | 604 | 0.056791 |
| c | TRBV4-2 | TRBJ1 | 22 | 0.002069 |
| c | TRBV4-2 | TRBJ2 | 23 | 0.002163 |
| c | TRBV4-2 | TRBJ3 | 15 | 0.00141 |
| c | TRBV4-2 | TRBJ4 | 170 | 0.015984 |
| c | TRBV5-12 | TRBJ1 | 122 | 0.011471 |
| c | TRBV5-12 | TRBJ2 | 154 | 0.01448 |
| c | TRBV5-12 | TRBJ3 | 329 | 0.030934 |
| c | TRBV5-12 | TRBJ4 | 272 | 0.025575 |
| c | TRBV5-12-1 | TRBJ1 | 675 | 0.063467 |
| c | TRBV5-12-1 | TRBJ2 | 1660 | 0.156081 |
| c | TRBV5-12-1 | TRBJ3 | 1464 | 0.137652 |
| c | TRBV5-12-1 | TRBJ4 | 1262 | 0.118659 |
| c | TRBV5-12-2 | TRBJ1 | 624 | 0.058671 |
| c | TRBV5-12-2 | TRBJ2 | 612 | 0.057543 |
| c | TRBV5-12-2 | TRBJ3 | 871 | 0.081895 |
| c | TRBV5-12-2 | TRBJ4 | 1026 | 0.096469 |
| c | TRBV5-15 | TRBJ1 | 741 | 0.069672 |
| c | TRBV5-15 | TRBJ2 | 548 | 0.051526 |
| c | TRBV5-15 | TRBJ3 | 222 | 0.020873 |
| c | TRBV5-15 | TRBJ4 | 363 | 0.034131 |
| c | TRBV5-16 | TRBJ1 | 46 | 0.004325 |
| c | TRBV5-16 | TRBJ2 | 95 | 0.008932 |
| c | TRBV5-16 | TRBJ3 | 83 | 0.007804 |
| c | TRBV5-16 | TRBJ4 | 115 | 0.010813 |
| c | TRBV5-17 | TRBJ1 | 2576 | 0.242207 |
| c | TRBV5-17 | TRBJ2 | 6344 | 0.596492 |
| c | TRBV5-17 | TRBJ3 | 6366 | 0.598561 |
| c | TRBV5-17 | TRBJ4 | 7790 | 0.732452 |
| c | TRBV5-2 | TRBJ1 | 403 | 0.037892 |
| c | TRBV5-2 | TRBJ2 | 1825 | 0.171595 |
| c | TRBV5-2 | TRBJ3 | 1065 | 0.100136 |
| c | TRBV5-2 | TRBJ4 | 1017 | 0.095623 |
| c | TRBV5-4 | TRBJ1 | 2383 | 0.224061 |
| c | TRBV5-4 | TRBJ2 | 1810 | 0.170185 |
| c | TRBV5-4 | TRBJ3 | 2349 | 0.220864 |
| c | TRBV5-4 | TRBJ4 | 2454 | 0.230736 |
| c | TRBV5-6 | TRBJ1 | 1582 | 0.148747 |
| c | TRBV5-6 | TRBJ2 | 4814 | 0.452635 |
| c | TRBV5-6 | TRBJ3 | 3434 | 0.322881 |
| c | TRBV5-6 | TRBJ4 | 4357 | 0.409665 |
| c | TRBV5-6-2 | TRBJ1 | 294 | 0.027643 |
| c | TRBV5-6-2 | TRBJ2 | 1199 | 0.112736 |
| c | TRBV5-6-2 | TRBJ3 | 973 | 0.091486 |
| c | TRBV5-6-2 | TRBJ4 | 645 | 0.060646 |
| c | TRBV5-6-3 | TRBJ1 | 1754 | 0.164919 |
| c | TRBV5-6-3 | TRBJ2 | 5323 | 0.500493 |
| c | TRBV5-6-3 | TRBJ3 | 4489 | 0.422077 |
| c | TRBV5-6-3 | TRBJ4 | 3639 | 0.342156 |
| c | TRBV5-6-4 | TRBJ1 | 144 | 0.01354 |
| c | TRBV5-6-4 | TRBJ2 | 362 | 0.034037 |
| c | TRBV5-6-4 | TRBJ3 | 356 | 0.033473 |
| c | TRBV5-6-4 | TRBJ4 | 315 | 0.029618 |
| c | TRBV5-6-5 | TRBJ1 | 255 | 0.023976 |
| c | TRBV5-6-5 | TRBJ2 | 732 | 0.068826 |
| c | TRBV5-6-5 | TRBJ3 | 574 | 0.05397 |
| c | TRBV5-6-5 | TRBJ4 | 535 | 0.050303 |
| c | TRBV5-9 | TRBJ1 | 232 | 0.021814 |
| c | TRBV5-9 | TRBJ2 | 662 | 0.062244 |
| c | TRBV5-9 | TRBJ3 | 495 | 0.046542 |
| c | TRBV5-9 | TRBJ4 | 612 | 0.057543 |
| c | TRBV5-9-1 | TRBJ1 | 208 | 0.019557 |
| c | TRBV5-9-1 | TRBJ2 | 477 | 0.04485 |
| c | TRBV5-9-1 | TRBJ3 | 557 | 0.052372 |
| c | TRBV5-9-1 | TRBJ4 | 524 | 0.049269 |
| c | TRBV5-9-2 | TRBJ1 | 51 | 0.004795 |
| c | TRBV5-9-2 | TRBJ2 | 117 | 0.011001 |
| c | TRBV5-9-2 | TRBJ3 | 121 | 0.011377 |
| c | TRBV5-9-2 | TRBJ4 | 117 | 0.011001 |
| c | TRBV5-9-3 | TRBJ1 | 604 | 0.056791 |
| c | TRBV5-9-3 | TRBJ2 | 4113 | 0.386723 |
| c | TRBV5-9-3 | TRBJ3 | 1856 | 0.17451 |
| c | TRBV5-9-3 | TRBJ4 | 1754 | 0.164919 |
| c | TRBV5-9-4 | TRBJ1 | 380 | 0.035729 |
| c | TRBV5-9-4 | TRBJ2 | 890 | 0.083682 |
| c | TRBV5-9-4 | TRBJ3 | 1247 | 0.117249 |
| c | TRBV5-9-4 | TRBJ4 | 1397 | 0.131352 |
| c | TRBV5S21 | TRBJ2 | 3 | 0.000282 |
| c | TRBV5S21 | TRBJ3 | 1 | 9.40E-05 |
| c | TRBV5S9 | TRBJ1 | 314 | 0.029524 |
| c | TRBV5S9 | TRBJ2 | 1569 | 0.147525 |
| c | TRBV5S9 | TRBJ3 | 667 | 0.062714 |
| c | TRBV5S9 | TRBJ4 | 596 | 0.056039 |
| c | TRBV6-3 | TRBJ1 | 36 | 0.003385 |
| c | TRBV6-3 | TRBJ2 | 281 | 0.026421 |
| c | TRBV6-3 | TRBJ3 | 284 | 0.026703 |
| c | TRBV6-3 | TRBJ4 | 166 | 0.015608 |
| c | TRBV6-6 | TRBJ1 | 1838 | 0.172817 |
| c | TRBV6-6 | TRBJ2 | 4251 | 0.399699 |
| c | TRBV6-6 | TRBJ3 | 7044 | 0.66231 |
| c | TRBV6-6 | TRBJ4 | 2569 | 0.241549 |
| c | TRBV6-7 | TRBJ3 | 1 | 9.40E-05 |
| c | TRBV6-7 | TRBJ4 | 1 | 9.40E-05 |
| c | TRBV7-1 | TRBJ1 | 3111 | 0.292511 |
| c | TRBV7-1 | TRBJ2 | 449 | 0.042217 |
| c | TRBV7-1 | TRBJ3 | 1504 | 0.141413 |
| c | TRBV7-1 | TRBJ4 | 1307 | 0.12289 |
| c | TRBV7-3 | TRBJ1 | 3067 | 0.288374 |
| c | TRBV7-3 | TRBJ2 | 2650 | 0.249165 |
| c | TRBV7-3 | TRBJ3 | 2730 | 0.256687 |
| c | TRBV7-3 | TRBJ4 | 2997 | 0.281792 |
| c | TRBV7-4 | TRBJ1 | 10 | 0.00094 |
| c | TRBV7-4 | TRBJ2 | 12 | 0.001128 |
| c | TRBV7-4 | TRBJ3 | 29 | 0.002727 |
| c | TRBV7-4 | TRBJ4 | 38 | 0.003573 |
| c | TRBV8-11 | TRBJ1 | 21 | 0.001975 |
| c | TRBV8-11 | TRBJ2 | 59 | 0.005547 |
| c | TRBV8-11 | TRBJ3 | 68 | 0.006394 |
| c | TRBV8-11 | TRBJ4 | 45 | 0.004231 |
| c | TRBV8-12 | TRBJ1 | 60 | 0.005641 |
| c | TRBV8-12 | TRBJ2 | 98 | 0.009214 |
| c | TRBV8-12 | TRBJ3 | 129 | 0.012129 |
| c | TRBV8-12 | TRBJ4 | 139 | 0.013069 |
| c | TRBV8-13 | TRBJ2 | 5 | 0.00047 |
| c | TRBV8-13 | TRBJ3 | 2 | 0.000188 |
| c | TRBV8-13 | TRBJ4 | 3 | 0.000282 |
| c | TRBV8-2 | TRBJ1 | 2 | 0.000188 |
| c | TRBV8-2 | TRBJ2 | 10 | 0.00094 |
| c | TRBV8-2 | TRBJ3 | 24 | 0.002257 |
| c | TRBV8-2 | TRBJ4 | 44 | 0.004137 |
| c | TRBV8-4 | TRBJ2 | 2 | 0.000188 |
| c | TRBV8-4 | TRBJ3 | 7 | 0.000658 |
| c | TRBV8-4 | TRBJ4 | 11 | 0.001034 |
| c | TRBV8-5 | TRBJ1 | 42 | 0.003949 |
| c | TRBV8-5 | TRBJ2 | 45 | 0.004231 |
| c | TRBV8-5 | TRBJ3 | 25 | 0.002351 |
| c | TRBV8-5 | TRBJ4 | 21 | 0.001975 |
| c | TRBV8-8 | TRBJ1 | 105 | 0.009873 |
| c | TRBV8-8 | TRBJ2 | 194 | 0.018241 |
| c | TRBV8-8 | TRBJ3 | 231 | 0.02172 |
| c | TRBV8-8 | TRBJ4 | 190 | 0.017865 |
| c | TRBV8-8-1 | TRBJ2 | 1 | 9.40E-05 |
| c | TRBV8-8-1 | TRBJ4 | 2 | 0.000188 |
| c | TRBV9-3-1 | TRBJ1 | 1 | 9.40E-05 |
| c | TRBV9-3-1 | TRBJ3 | 3 | 0.000282 |
| x | TRBV1-11 | TRBJ1 | 50 | 0.004532 |
| x | TRBV1-11 | TRBJ2 | 982 | 0.089015 |
| x | TRBV1-11 | TRBJ3 | 169 | 0.015319 |
| x | TRBV1-11 | TRBJ4 | 296 | 0.026831 |
| x | TRBV1-12 | TRBJ1 | 43 | 0.003898 |
| x | TRBV1-12 | TRBJ2 | 112 | 0.010152 |
| x | TRBV1-12 | TRBJ3 | 136 | 0.012328 |
| x | TRBV1-12 | TRBJ4 | 186 | 0.01686 |
| x | TRBV1-13 | TRBJ2 | 2 | 0.000181 |
| x | TRBV1-13 | TRBJ3 | 1 | 9.06E-05 |
| x | TRBV1-13 | TRBJ4 | 4 | 0.000363 |
| x | TRBV1-14 | TRBJ1 | 2848 | 0.258163 |
| x | TRBV1-14 | TRBJ2 | 7616 | 0.690367 |
| x | TRBV1-14 | TRBJ3 | 8968 | 0.812922 |
| x | TRBV1-14 | TRBJ4 | 8025 | 0.727442 |
| x | TRBV1-15 | TRBJ1 | 3312 | 0.300223 |
| x | TRBV1-15 | TRBJ2 | 5374 | 0.487137 |
| x | TRBV1-15 | TRBJ3 | 4838 | 0.43855 |
| x | TRBV1-15 | TRBJ4 | 4556 | 0.412988 |
| x | TRBV1-16 | TRBJ2 | 1 | 9.06E-05 |
| x | TRBV1-18 | TRBJ1 | 1775 | 0.160898 |
| x | TRBV1-18 | TRBJ2 | 4844 | 0.439094 |
| x | TRBV1-18 | TRBJ3 | 5024 | 0.45541 |
| x | TRBV1-18 | TRBJ4 | 4534 | 0.410993 |
| x | TRBV1-19 | TRBJ1 | 80 | 0.007252 |
| x | TRBV1-19 | TRBJ2 | 270 | 0.024475 |
| x | TRBV1-19 | TRBJ3 | 207 | 0.018764 |
| x | TRBV1-19 | TRBJ4 | 271 | 0.024565 |
| x | TRBV1-20 | TRBJ1 | 507 | 0.045958 |
| x | TRBV1-20 | TRBJ2 | 1369 | 0.124096 |
| x | TRBV1-20 | TRBJ3 | 1222 | 0.110771 |
| x | TRBV1-20 | TRBJ4 | 1403 | 0.127178 |
| x | TRBV1-21 | TRBJ2 | 1 | 9.06E-05 |
| x | TRBV1-22 | TRBJ1 | 48 | 0.004351 |
| x | TRBV1-22 | TRBJ2 | 165 | 0.014957 |
| x | TRBV1-22 | TRBJ3 | 124 | 0.01124 |
| x | TRBV1-22 | TRBJ4 | 116 | 0.010515 |
| x | TRBV1-23 | TRBJ2 | 1 | 9.06E-05 |
| x | TRBV1-23 | TRBJ3 | 5 | 0.000453 |
| x | TRBV1-23 | TRBJ4 | 4 | 0.000363 |
| x | TRBV1-25 | TRBJ1 | 14 | 0.001269 |
| x | TRBV1-25 | TRBJ2 | 27 | 0.002447 |
| x | TRBV1-25 | TRBJ3 | 30 | 0.002719 |
| x | TRBV1-25 | TRBJ4 | 27 | 0.002447 |
| x | TRBV1-26 | TRBJ1 | 850 | 0.07705 |
| x | TRBV1-26 | TRBJ2 | 2119 | 0.192081 |
| x | TRBV1-26 | TRBJ3 | 1911 | 0.173226 |
| x | TRBV1-26 | TRBJ4 | 2315 | 0.209848 |
| x | TRBV1-28 | TRBJ1 | 14 | 0.001269 |
| x | TRBV1-28 | TRBJ2 | 10 | 0.000906 |
| x | TRBV1-28 | TRBJ3 | 8 | 0.000725 |
| x | TRBV1-28 | TRBJ4 | 3 | 0.000272 |
| x | TRBV1-31 | TRBJ1 | 166 | 0.015047 |
| x | TRBV1-31 | TRBJ2 | 336 | 0.030457 |
| x | TRBV1-31 | TRBJ3 | 307 | 0.027829 |
| x | TRBV1-31 | TRBJ4 | 416 | 0.037709 |
| x | TRBV1-34 | TRBJ1 | 69 | 0.006255 |
| x | TRBV1-34 | TRBJ2 | 141 | 0.012781 |
| x | TRBV1-34 | TRBJ3 | 127 | 0.011512 |
| x | TRBV1-34 | TRBJ4 | 217 | 0.01967 |
| x | TRBV1-36 | TRBJ1 | 10 | 0.000906 |
| x | TRBV1-36 | TRBJ2 | 15 | 0.00136 |
| x | TRBV1-36 | TRBJ3 | 7 | 0.000635 |
| x | TRBV1-36 | TRBJ4 | 6 | 0.000544 |
| x | TRBV1-37 | TRBJ1 | 778 | 0.070523 |
| x | TRBV1-37 | TRBJ2 | 2359 | 0.213836 |
| x | TRBV1-37 | TRBJ3 | 1752 | 0.158813 |
| x | TRBV1-37 | TRBJ4 | 2832 | 0.256712 |
| x | TRBV1-39 | TRBJ1 | 606 | 0.054932 |
| x | TRBV1-39 | TRBJ2 | 1557 | 0.141137 |
| x | TRBV1-39 | TRBJ3 | 1351 | 0.122464 |
| x | TRBV1-39 | TRBJ4 | 1736 | 0.157363 |
| x | TRBV1-4 | TRBJ1 | 2061 | 0.186823 |
| x | TRBV1-4 | TRBJ2 | 5558 | 0.503816 |
| x | TRBV1-4 | TRBJ3 | 4242 | 0.384524 |
| x | TRBV1-4 | TRBJ4 | 4022 | 0.364582 |
| x | TRBV1-42 | TRBJ1 | 90 | 0.008158 |
| x | TRBV1-42 | TRBJ2 | 559 | 0.050672 |
| x | TRBV1-42 | TRBJ3 | 312 | 0.028282 |
| x | TRBV1-42 | TRBJ4 | 336 | 0.030457 |
| x | TRBV1-43 | TRBJ1 | 6 | 0.000544 |
| x | TRBV1-43 | TRBJ2 | 18 | 0.001632 |
| x | TRBV1-43 | TRBJ3 | 26 | 0.002357 |
| x | TRBV1-43 | TRBJ4 | 23 | 0.002085 |
| x | TRBV1-47 | TRBJ1 | 265 | 0.024021 |
| x | TRBV1-47 | TRBJ2 | 903 | 0.081854 |
| x | TRBV1-47 | TRBJ3 | 3827 | 0.346906 |
| x | TRBV1-47 | TRBJ4 | 696 | 0.06309 |
| x | TRBV1-49 | TRBJ3 | 1 | 9.06E-05 |
| x | TRBV1-5 | TRBJ1 | 1208 | 0.109502 |
| x | TRBV1-5 | TRBJ2 | 3743 | 0.339292 |
| x | TRBV1-5 | TRBJ3 | 2656 | 0.240758 |
| x | TRBV1-5 | TRBJ4 | 3113 | 0.282184 |
| x | TRBV1-50 | TRBJ1 | 9 | 0.000816 |
| x | TRBV1-50 | TRBJ2 | 15 | 0.00136 |
| x | TRBV1-50 | TRBJ3 | 16 | 0.00145 |
| x | TRBV1-50 | TRBJ4 | 23 | 0.002085 |
| x | TRBV1-52 | TRBJ1 | 15 | 0.00136 |
| x | TRBV1-52 | TRBJ2 | 75 | 0.006799 |
| x | TRBV1-52 | TRBJ3 | 59 | 0.005348 |
| x | TRBV1-52 | TRBJ4 | 27 | 0.002447 |
| x | TRBV1-53 | TRBJ1 | 440 | 0.039885 |
| x | TRBV1-53 | TRBJ2 | 1136 | 0.102975 |
| x | TRBV1-53 | TRBJ3 | 1051 | 0.09527 |
| x | TRBV1-53 | TRBJ4 | 1146 | 0.103881 |
| x | TRBV1-54 | TRBJ1 | 1779 | 0.161261 |
| x | TRBV1-54 | TRBJ2 | 5487 | 0.49738 |
| x | TRBV1-54 | TRBJ3 | 3818 | 0.34609 |
| x | TRBV1-54 | TRBJ4 | 4880 | 0.442357 |
| x | TRBV1-55 | TRBJ1 | 703 | 0.063725 |
| x | TRBV1-55 | TRBJ2 | 1127 | 0.102159 |
| x | TRBV1-55 | TRBJ3 | 1402 | 0.127087 |
| x | TRBV1-55 | TRBJ4 | 1046 | 0.094817 |
| x | TRBV1-56 | TRBJ1 | 16 | 0.00145 |
| x | TRBV1-56 | TRBJ2 | 45 | 0.004079 |
| x | TRBV1-56 | TRBJ3 | 47 | 0.00426 |
| x | TRBV1-56 | TRBJ4 | 45 | 0.004079 |
| x | TRBV1-58 | TRBJ2 | 1 | 9.06E-05 |
| x | TRBV1-59 | TRBJ1 | 49 | 0.004442 |
| x | TRBV1-59 | TRBJ2 | 140 | 0.012691 |
| x | TRBV1-59 | TRBJ3 | 99 | 0.008974 |
| x | TRBV1-59 | TRBJ4 | 93 | 0.00843 |
| x | TRBV1-61 | TRBJ1 | 241 | 0.021846 |
| x | TRBV1-61 | TRBJ2 | 921 | 0.083486 |
| x | TRBV1-61 | TRBJ3 | 993 | 0.090012 |
| x | TRBV1-61 | TRBJ4 | 655 | 0.059374 |
| x | TRBV1-62-2 | TRBJ1 | 439 | 0.039794 |
| x | TRBV1-62-2 | TRBJ2 | 2192 | 0.198698 |
| x | TRBV1-62-2 | TRBJ3 | 1145 | 0.103791 |
| x | TRBV1-62-2 | TRBJ4 | 1501 | 0.136061 |
| x | TRBV1-62-3 | TRBJ1 | 27 | 0.002447 |
| x | TRBV1-62-3 | TRBJ2 | 27 | 0.002447 |
| x | TRBV1-62-3 | TRBJ3 | 39 | 0.003535 |
| x | TRBV1-62-3 | TRBJ4 | 27 | 0.002447 |
| x | TRBV1-63 | TRBJ1 | 1089 | 0.098715 |
| x | TRBV1-63 | TRBJ2 | 1432 | 0.129806 |
| x | TRBV1-63 | TRBJ3 | 1334 | 0.120923 |
| x | TRBV1-63 | TRBJ4 | 1138 | 0.103156 |
| x | TRBV1-64 | TRBJ1 | 24 | 0.002176 |
| x | TRBV1-64 | TRBJ2 | 73 | 0.006617 |
| x | TRBV1-64 | TRBJ3 | 80 | 0.007252 |
| x | TRBV1-64 | TRBJ4 | 65 | 0.005892 |
| x | TRBV1-66 | TRBJ1 | 167 | 0.015138 |
| x | TRBV1-66 | TRBJ2 | 477 | 0.043239 |
| x | TRBV1-66 | TRBJ3 | 367 | 0.033267 |
| x | TRBV1-66 | TRBJ4 | 395 | 0.035806 |
| x | TRBV1-67 | TRBJ1 | 1152 | 0.104425 |
| x | TRBV1-67 | TRBJ2 | 3475 | 0.314998 |
| x | TRBV1-67 | TRBJ3 | 2300 | 0.208488 |
| x | TRBV1-67 | TRBJ4 | 4677 | 0.423956 |
| x | TRBV1-69 | TRBJ1 | 644 | 0.058377 |
| x | TRBV1-69 | TRBJ2 | 2583 | 0.234141 |
| x | TRBV1-69 | TRBJ3 | 2364 | 0.214289 |
| x | TRBV1-69 | TRBJ4 | 2056 | 0.18637 |
| x | TRBV1-7 | TRBJ1 | 4370 | 0.396127 |
| x | TRBV1-7 | TRBJ2 | 16155 | 1.464402 |
| x | TRBV1-7 | TRBJ3 | 11774 | 1.067277 |
| x | TRBV1-7 | TRBJ4 | 13373 | 1.212222 |
| x | TRBV1-72 | TRBJ1 | 1 | 9.06E-05 |
| x | TRBV1-72 | TRBJ4 | 1 | 9.06E-05 |
| x | TRBV1-74 | TRBJ1 | 814 | 0.073787 |
| x | TRBV1-74 | TRBJ2 | 2531 | 0.229427 |
| x | TRBV1-74 | TRBJ3 | 2043 | 0.185192 |
| x | TRBV1-74 | TRBJ4 | 2957 | 0.268043 |
| x | TRBV1-75 | TRBJ2 | 9 | 0.000816 |
| x | TRBV1-75 | TRBJ3 | 9 | 0.000816 |
| x | TRBV1-75 | TRBJ4 | 16 | 0.00145 |
| x | TRBV1-76 | TRBJ1 | 97 | 0.008793 |
| x | TRBV1-76 | TRBJ2 | 179 | 0.016226 |
| x | TRBV1-76 | TRBJ3 | 147 | 0.013325 |
| x | TRBV1-76 | TRBJ4 | 206 | 0.018673 |
| x | TRBV1-77 | TRBJ1 | 480 | 0.043511 |
| x | TRBV1-77 | TRBJ2 | 1152 | 0.104425 |
| x | TRBV1-77 | TRBJ3 | 1066 | 0.09663 |
| x | TRBV1-77 | TRBJ4 | 978 | 0.088653 |
| x | TRBV1-78 | TRBJ1 | 35 | 0.003173 |
| x | TRBV1-78 | TRBJ2 | 94 | 0.008521 |
| x | TRBV1-78 | TRBJ3 | 70 | 0.006345 |
| x | TRBV1-78 | TRBJ4 | 87 | 0.007886 |
| x | TRBV1-8 | TRBJ3 | 2 | 0.000181 |
| x | TRBV1-8 | TRBJ4 | 1 | 9.06E-05 |
| x | TRBV1-80 | TRBJ1 | 1532 | 0.138871 |
| x | TRBV1-80 | TRBJ2 | 4676 | 0.423865 |
| x | TRBV1-80 | TRBJ3 | 3461 | 0.313729 |
| x | TRBV1-80 | TRBJ4 | 7249 | 0.6571 |
| x | TRBV1-81 | TRBJ1 | 241 | 0.021846 |
| x | TRBV1-81 | TRBJ2 | 709 | 0.064269 |
| x | TRBV1-81 | TRBJ3 | 683 | 0.061912 |
| x | TRBV1-81 | TRBJ4 | 601 | 0.054479 |
| x | TRBV1-82 | TRBJ1 | 113 | 0.010243 |
| x | TRBV1-82 | TRBJ2 | 366 | 0.033177 |
| x | TRBV1-82 | TRBJ3 | 375 | 0.033993 |
| x | TRBV1-82 | TRBJ4 | 376 | 0.034083 |
| x | TRBV1-83 | TRBJ1 | 149 | 0.013506 |
| x | TRBV1-83 | TRBJ2 | 439 | 0.039794 |
| x | TRBV1-83 | TRBJ3 | 361 | 0.032724 |
| x | TRBV1-83 | TRBJ4 | 368 | 0.033358 |
| x | TRBV1-84 | TRBJ1 | 326 | 0.029551 |
| x | TRBV1-84 | TRBJ2 | 825 | 0.074784 |
| x | TRBV1-84 | TRBJ3 | 683 | 0.061912 |
| x | TRBV1-84 | TRBJ4 | 642 | 0.058195 |
| x | TRBV1-85 | TRBJ1 | 307 | 0.027829 |
| x | TRBV1-85 | TRBJ2 | 860 | 0.077956 |
| x | TRBV1-85 | TRBJ3 | 822 | 0.074512 |
| x | TRBV1-85 | TRBJ4 | 764 | 0.069254 |
| x | TRBV1-87 | TRBJ1 | 1851 | 0.167788 |
| x | TRBV1-87 | TRBJ2 | 4432 | 0.401747 |
| x | TRBV1-87 | TRBJ3 | 4169 | 0.377907 |
| x | TRBV1-87 | TRBJ4 | 4755 | 0.431026 |
| x | TRBV1-9 | TRBJ1 | 6822 | 0.618394 |
| x | TRBV1-9 | TRBJ2 | 19107 | 1.731991 |
| x | TRBV1-9 | TRBJ3 | 15037 | 1.363058 |
| x | TRBV1-9 | TRBJ4 | 14072 | 1.275584 |
| x | TRBV10-1 | TRBJ1 | 1471 | 0.133342 |
| x | TRBV10-1 | TRBJ2 | 4446 | 0.403016 |
| x | TRBV10-1 | TRBJ3 | 4847 | 0.439366 |
| x | TRBV10-1 | TRBJ4 | 6391 | 0.579325 |
| x | TRBV10-3 | TRBJ1 | 123 | 0.01115 |
| x | TRBV10-3 | TRBJ2 | 120 | 0.010878 |
| x | TRBV10-3 | TRBJ3 | 370 | 0.033539 |
| x | TRBV10-3 | TRBJ4 | 654 | 0.059283 |
| x | TRBV10S3 | TRBJ1 | 57 | 0.005167 |
| x | TRBV10S3 | TRBJ2 | 114 | 0.010334 |
| x | TRBV10S3 | TRBJ3 | 178 | 0.016135 |
| x | TRBV10S3 | TRBJ4 | 122 | 0.011059 |
| x | TRBV11-2 | TRBJ1 | 5 | 0.000453 |
| x | TRBV11-2 | TRBJ2 | 1 | 9.06E-05 |
| x | TRBV12-3 | TRBJ1 | 253 | 0.022934 |
| x | TRBV12-3 | TRBJ2 | 38 | 0.003445 |
| x | TRBV12-3 | TRBJ3 | 22 | 0.001994 |
| x | TRBV12-3 | TRBJ4 | 152 | 0.013778 |
| x | TRBV13-2 | TRBJ1 | 1 | 9.06E-05 |
| x | TRBV13-2 | TRBJ2 | 5 | 0.000453 |
| x | TRBV13-2 | TRBJ3 | 8 | 0.000725 |
| x | TRBV13-2 | TRBJ4 | 2 | 0.000181 |
| x | TRBV14-1 | TRBJ1 | 1578 | 0.143041 |
| x | TRBV14-1 | TRBJ2 | 5342 | 0.484236 |
| x | TRBV14-1 | TRBJ3 | 4400 | 0.398847 |
| x | TRBV14-1 | TRBJ4 | 2753 | 0.249551 |
| x | TRBV14-2 | TRBJ1 | 16 | 0.00145 |
| x | TRBV14-2 | TRBJ2 | 12 | 0.001088 |
| x | TRBV14-2 | TRBJ3 | 12 | 0.001088 |
| x | TRBV14-2 | TRBJ4 | 9 | 0.000816 |
| x | TRBV14-3 | TRBJ1 | 23991 | 2.174711 |
| x | TRBV14-3 | TRBJ2 | 43729 | 3.963901 |
| x | TRBV14-3 | TRBJ3 | 28374 | 2.572017 |
| x | TRBV14-3 | TRBJ4 | 25933 | 2.350748 |
| x | TRBV14-4 | TRBJ1 | 1762 | 0.15972 |
| x | TRBV14-4 | TRBJ2 | 7039 | 0.638064 |
| x | TRBV14-4 | TRBJ3 | 7388 | 0.6697 |
| x | TRBV14-4 | TRBJ4 | 5448 | 0.493845 |
| x | TRBV1S10 | TRBJ2 | 1 | 9.06E-05 |
| x | TRBV1S10 | TRBJ3 | 1 | 9.06E-05 |
| x | TRBV1S11 | TRBJ2 | 2 | 0.000181 |
| x | TRBV1S11 | TRBJ3 | 2 | 0.000181 |
| x | TRBV1S12 | TRBJ1 | 24 | 0.002176 |
| x | TRBV1S12 | TRBJ2 | 48 | 0.004351 |
| x | TRBV1S12 | TRBJ3 | 63 | 0.005711 |
| x | TRBV1S12 | TRBJ4 | 48 | 0.004351 |
| x | TRBV1S126 | TRBJ1 | 67 | 0.006073 |
| x | TRBV1S126 | TRBJ2 | 222 | 0.020124 |
| x | TRBV1S126 | TRBJ3 | 187 | 0.016951 |
| x | TRBV1S126 | TRBJ4 | 203 | 0.018401 |
| x | TRBV1S127 | TRBJ1 | 335 | 0.030367 |
| x | TRBV1S127 | TRBJ2 | 1245 | 0.112855 |
| x | TRBV1S127 | TRBJ3 | 920 | 0.083395 |
| x | TRBV1S127 | TRBJ4 | 761 | 0.068982 |
| x | TRBV1S130 | TRBJ1 | 679 | 0.061549 |
| x | TRBV1S130 | TRBJ2 | 1389 | 0.125909 |
| x | TRBV1S130 | TRBJ3 | 1433 | 0.129897 |
| x | TRBV1S130 | TRBJ4 | 1283 | 0.1163 |
| x | TRBV1S132 | TRBJ1 | 189 | 0.017132 |
| x | TRBV1S132 | TRBJ2 | 631 | 0.057198 |
| x | TRBV1S132 | TRBJ3 | 665 | 0.06028 |
| x | TRBV1S132 | TRBJ4 | 493 | 0.044689 |
| x | TRBV1S134 | TRBJ1 | 16 | 0.00145 |
| x | TRBV1S134 | TRBJ2 | 172 | 0.015591 |
| x | TRBV1S134 | TRBJ3 | 40 | 0.003626 |
| x | TRBV1S134 | TRBJ4 | 43 | 0.003898 |
| x | TRBV1S135 | TRBJ1 | 1431 | 0.129716 |
| x | TRBV1S135 | TRBJ2 | 2533 | 0.229609 |
| x | TRBV1S135 | TRBJ3 | 2526 | 0.228974 |
| x | TRBV1S135 | TRBJ4 | 3010 | 0.272847 |
| x | TRBV1S136 | TRBJ1 | 242 | 0.021937 |
| x | TRBV1S136 | TRBJ2 | 625 | 0.056654 |
| x | TRBV1S136 | TRBJ3 | 577 | 0.052303 |
| x | TRBV1S136 | TRBJ4 | 3716 | 0.336844 |
| x | TRBV1S137 | TRBJ1 | 742 | 0.06726 |
| x | TRBV1S137 | TRBJ2 | 1657 | 0.150202 |
| x | TRBV1S137 | TRBJ3 | 1572 | 0.142497 |
| x | TRBV1S137 | TRBJ4 | 2251 | 0.204046 |
| x | TRBV1S14 | TRBJ1 | 23 | 0.002085 |
| x | TRBV1S14 | TRBJ2 | 30 | 0.002719 |
| x | TRBV1S14 | TRBJ3 | 23 | 0.002085 |
| x | TRBV1S14 | TRBJ4 | 32 | 0.002901 |
| x | TRBV1S16 | TRBJ1 | 248 | 0.02248 |
| x | TRBV1S16 | TRBJ2 | 906 | 0.082126 |
| x | TRBV1S16 | TRBJ3 | 1033 | 0.093638 |
| x | TRBV1S16 | TRBJ4 | 674 | 0.061096 |
| x | TRBV1S17 | TRBJ1 | 3 | 0.000272 |
| x | TRBV1S17 | TRBJ2 | 16 | 0.00145 |
| x | TRBV1S17 | TRBJ3 | 21 | 0.001904 |
| x | TRBV1S17 | TRBJ4 | 25 | 0.002266 |
| x | TRBV1S18 | TRBJ2 | 1 | 9.06E-05 |
| x | TRBV1S18 | TRBJ3 | 1 | 9.06E-05 |
| x | TRBV1S19 | TRBJ1 | 6 | 0.000544 |
| x | TRBV1S19 | TRBJ2 | 10 | 0.000906 |
| x | TRBV1S19 | TRBJ3 | 9 | 0.000816 |
| x | TRBV1S19 | TRBJ4 | 10 | 0.000906 |
| x | TRBV1S22 | TRBJ1 | 917 | 0.083123 |
| x | TRBV1S22 | TRBJ2 | 1953 | 0.177034 |
| x | TRBV1S22 | TRBJ3 | 3007 | 0.272575 |
| x | TRBV1S22 | TRBJ4 | 2713 | 0.245925 |
| x | TRBV1S26 | TRBJ1 | 400 | 0.036259 |
| x | TRBV1S26 | TRBJ2 | 1357 | 0.123008 |
| x | TRBV1S26 | TRBJ3 | 992 | 0.089922 |
| x | TRBV1S26 | TRBJ4 | 1114 | 0.100981 |
| x | TRBV1S28 | TRBJ2 | 1 | 9.06E-05 |
| x | TRBV1S28 | TRBJ3 | 1 | 9.06E-05 |
| x | TRBV1S28 | TRBJ4 | 2 | 0.000181 |
| x | TRBV1S29 | TRBJ1 | 1336 | 0.121104 |
| x | TRBV1S29 | TRBJ2 | 3151 | 0.285629 |
| x | TRBV1S29 | TRBJ3 | 3350 | 0.303667 |
| x | TRBV1S29 | TRBJ4 | 6234 | 0.565093 |
| x | TRBV1S30 | TRBJ1 | 24 | 0.002176 |
| x | TRBV1S30 | TRBJ2 | 66 | 0.005983 |
| x | TRBV1S30 | TRBJ3 | 53 | 0.004804 |
| x | TRBV1S30 | TRBJ4 | 65 | 0.005892 |
| x | TRBV1S34 | TRBJ1 | 1209 | 0.109592 |
| x | TRBV1S34 | TRBJ2 | 1953 | 0.177034 |
| x | TRBV1S34 | TRBJ3 | 1860 | 0.168603 |
| x | TRBV1S34 | TRBJ4 | 2590 | 0.234776 |
| x | TRBV1S35 | TRBJ2 | 1 | 9.06E-05 |
| x | TRBV1S35 | TRBJ4 | 1 | 9.06E-05 |
| x | TRBV1S36 | TRBJ1 | 24 | 0.002176 |
| x | TRBV1S36 | TRBJ2 | 22 | 0.001994 |
| x | TRBV1S36 | TRBJ3 | 17 | 0.001541 |
| x | TRBV1S36 | TRBJ4 | 15 | 0.00136 |
| x | TRBV1S40 | TRBJ1 | 12 | 0.001088 |
| x | TRBV1S40 | TRBJ2 | 28 | 0.002538 |
| x | TRBV1S40 | TRBJ3 | 29 | 0.002629 |
| x | TRBV1S40 | TRBJ4 | 33 | 0.002991 |
| x | TRBV1S41 | TRBJ1 | 1069 | 0.096902 |
| x | TRBV1S41 | TRBJ2 | 3562 | 0.322884 |
| x | TRBV1S41 | TRBJ3 | 2621 | 0.237586 |
| x | TRBV1S41 | TRBJ4 | 2616 | 0.237132 |
| x | TRBV1S45 | TRBJ1 | 237 | 0.021483 |
| x | TRBV1S45 | TRBJ2 | 2012 | 0.182382 |
| x | TRBV1S45 | TRBJ3 | 377 | 0.034174 |
| x | TRBV1S45 | TRBJ4 | 585 | 0.053028 |
| x | TRBV1S46 | TRBJ1 | 19 | 0.001722 |
| x | TRBV1S46 | TRBJ2 | 30 | 0.002719 |
| x | TRBV1S46 | TRBJ3 | 27 | 0.002447 |
| x | TRBV1S46 | TRBJ4 | 23 | 0.002085 |
| x | TRBV1S5 | TRBJ1 | 19 | 0.001722 |
| x | TRBV1S5 | TRBJ2 | 51 | 0.004623 |
| x | TRBV1S5 | TRBJ3 | 67 | 0.006073 |
| x | TRBV1S5 | TRBJ4 | 51 | 0.004623 |
| x | TRBV1S50 | TRBJ1 | 4 | 0.000363 |
| x | TRBV1S50 | TRBJ2 | 12 | 0.001088 |
| x | TRBV1S50 | TRBJ3 | 12 | 0.001088 |
| x | TRBV1S50 | TRBJ4 | 11 | 0.000997 |
| x | TRBV1S51 | TRBJ2 | 1 | 9.06E-05 |
| x | TRBV1S51 | TRBJ3 | 1 | 9.06E-05 |
| x | TRBV1S51 | TRBJ4 | 2 | 0.000181 |
| x | TRBV1S52 | TRBJ1 | 1 | 9.06E-05 |
| x | TRBV1S52 | TRBJ2 | 1 | 9.06E-05 |
| x | TRBV1S52 | TRBJ3 | 1 | 9.06E-05 |
| x | TRBV1S52 | TRBJ4 | 4 | 0.000363 |
| x | TRBV1S53 | TRBJ1 | 813 | 0.073696 |
| x | TRBV1S53 | TRBJ2 | 4949 | 0.448612 |
| x | TRBV1S53 | TRBJ3 | 3392 | 0.307474 |
| x | TRBV1S53 | TRBJ4 | 4066 | 0.368571 |
| x | TRBV1S55 | TRBJ1 | 6 | 0.000544 |
| x | TRBV1S55 | TRBJ2 | 14 | 0.001269 |
| x | TRBV1S55 | TRBJ3 | 15 | 0.00136 |
| x | TRBV1S55 | TRBJ4 | 20 | 0.001813 |
| x | TRBV1S56 | TRBJ1 | 1068 | 0.096811 |
| x | TRBV1S56 | TRBJ2 | 2092 | 0.189633 |
| x | TRBV1S56 | TRBJ3 | 2307 | 0.209123 |
| x | TRBV1S56 | TRBJ4 | 3465 | 0.314092 |
| x | TRBV1S61 | TRBJ1 | 1 | 9.06E-05 |
| x | TRBV1S61 | TRBJ4 | 1 | 9.06E-05 |
| x | TRBV1S72 | TRBJ2 | 1 | 9.06E-05 |
| x | TRBV1S81 | TRBJ1 | 2611 | 0.236679 |
| x | TRBV1S81 | TRBJ2 | 8168 | 0.740404 |
| x | TRBV1S81 | TRBJ3 | 9574 | 0.867854 |
| x | TRBV1S81 | TRBJ4 | 7430 | 0.673507 |
| x | TRBV1S82 | TRBJ1 | 69 | 0.006255 |
| x | TRBV1S82 | TRBJ2 | 420 | 0.038072 |
| x | TRBV1S82 | TRBJ3 | 224 | 0.020305 |
| x | TRBV1S82 | TRBJ4 | 157 | 0.014232 |
| x | TRBV1S9 | TRBJ1 | 1 | 9.06E-05 |
| x | TRBV1S9 | TRBJ2 | 7 | 0.000635 |
| x | TRBV1S9 | TRBJ3 | 4 | 0.000363 |
| x | TRBV1S9 | TRBJ4 | 8 | 0.000725 |
| x | TRBV2-2 | TRBJ1 | 3433 | 0.311191 |
| x | TRBV2-2 | TRBJ2 | 5547 | 0.502819 |
| x | TRBV2-2 | TRBJ3 | 5447 | 0.493754 |
| x | TRBV2-2 | TRBJ4 | 6303 | 0.571348 |
| x | TRBV2-2-1 | TRBJ2 | 5 | 0.000453 |
| x | TRBV2-2-1 | TRBJ3 | 6 | 0.000544 |
| x | TRBV2-2-1 | TRBJ4 | 11 | 0.000997 |
| x | TRBV2-2-2 | TRBJ1 | 18 | 0.001632 |
| x | TRBV2-2-2 | TRBJ2 | 36 | 0.003263 |
| x | TRBV2-2-2 | TRBJ3 | 13 | 0.001178 |
| x | TRBV2-2-2 | TRBJ4 | 56 | 0.005076 |
| x | TRBV2-3 | TRBJ1 | 2241 | 0.20314 |
| x | TRBV2-3 | TRBJ2 | 2223 | 0.201508 |
| x | TRBV2-3 | TRBJ3 | 4581 | 0.415254 |
| x | TRBV2-3 | TRBJ4 | 5496 | 0.498196 |
| x | TRBV2-3-1 | TRBJ1 | 1 | 9.06E-05 |
| x | TRBV2-3-1 | TRBJ3 | 2 | 0.000181 |
| x | TRBV2-3-1 | TRBJ4 | 2 | 0.000181 |
| x | TRBV2-4 | TRBJ1 | 408 | 0.036984 |
| x | TRBV2-4 | TRBJ2 | 1039 | 0.094182 |
| x | TRBV2-4 | TRBJ3 | 756 | 0.068529 |
| x | TRBV2-4 | TRBJ4 | 994 | 0.090103 |
| x | TRBV2-4-1 | TRBJ1 | 536 | 0.048587 |
| x | TRBV2-4-1 | TRBJ2 | 1558 | 0.141228 |
| x | TRBV2-4-1 | TRBJ3 | 2212 | 0.200511 |
| x | TRBV2-4-1 | TRBJ4 | 2250 | 0.203956 |
| x | TRBV2-5 | TRBJ1 | 926 | 0.083939 |
| x | TRBV2-5 | TRBJ2 | 1459 | 0.132254 |
| x | TRBV2-5 | TRBJ3 | 2390 | 0.216646 |
| x | TRBV2-5 | TRBJ4 | 4696 | 0.425678 |
| x | TRBV2-5-1 | TRBJ1 | 178 | 0.016135 |
| x | TRBV2-5-1 | TRBJ2 | 405 | 0.036712 |
| x | TRBV2-5-1 | TRBJ3 | 812 | 0.073605 |
| x | TRBV2-5-1 | TRBJ4 | 959 | 0.08693 |
| x | TRBV2-6 | TRBJ1 | 766 | 0.069436 |
| x | TRBV2-6 | TRBJ2 | 956 | 0.086658 |
| x | TRBV2-6 | TRBJ3 | 1697 | 0.153828 |
| x | TRBV2-6 | TRBJ4 | 3317 | 0.300676 |
| x | TRBV2-6-1 | TRBJ1 | 834 | 0.0756 |
| x | TRBV2-6-1 | TRBJ2 | 601 | 0.054479 |
| x | TRBV2-6-1 | TRBJ3 | 1192 | 0.108051 |
| x | TRBV2-6-1 | TRBJ4 | 4595 | 0.416523 |
| x | TRBV2-6-2 | TRBJ1 | 614 | 0.055657 |
| x | TRBV2-6-2 | TRBJ2 | 484 | 0.043873 |
| x | TRBV2-6-2 | TRBJ3 | 816 | 0.073968 |
| x | TRBV2-6-2 | TRBJ4 | 1892 | 0.171504 |
| x | TRBV2-6-4 | TRBJ1 | 303 | 0.027466 |
| x | TRBV2-6-4 | TRBJ2 | 367 | 0.033267 |
| x | TRBV2-6-4 | TRBJ3 | 654 | 0.059283 |
| x | TRBV2-6-4 | TRBJ4 | 837 | 0.075872 |
| x | TRBV2-6-5 | TRBJ1 | 433 | 0.03925 |
| x | TRBV2-6-5 | TRBJ2 | 817 | 0.074059 |
| x | TRBV2-6-5 | TRBJ3 | 1584 | 0.143585 |
| x | TRBV2-6-5 | TRBJ4 | 1159 | 0.10506 |
| x | TRBV2-6-6 | TRBJ1 | 6 | 0.000544 |
| x | TRBV2-6-6 | TRBJ2 | 20 | 0.001813 |
| x | TRBV2-6-6 | TRBJ3 | 53 | 0.004804 |
| x | TRBV2-6-6 | TRBJ4 | 52 | 0.004714 |
| x | TRBV2-6-7 | TRBJ1 | 3477 | 0.315179 |
| x | TRBV2-6-7 | TRBJ2 | 3276 | 0.296959 |
| x | TRBV2-6-7 | TRBJ3 | 18084 | 1.63926 |
| x | TRBV2-6-7 | TRBJ4 | 7969 | 0.722366 |
| x | TRBV2-9 | TRBJ1 | 6286 | 0.569807 |
| x | TRBV2-9 | TRBJ2 | 13658 | 1.238056 |
| x | TRBV2-9 | TRBJ3 | 24898 | 2.256928 |
| x | TRBV2-9 | TRBJ4 | 21558 | 1.954167 |
| x | TRBV2-9-1 | TRBJ1 | 4 | 0.000363 |
| x | TRBV2-9-1 | TRBJ2 | 8 | 0.000725 |
| x | TRBV2-9-1 | TRBJ3 | 24 | 0.002176 |
| x | TRBV2-9-1 | TRBJ4 | 21 | 0.001904 |
| x | TRBV2-9-2 | TRBJ1 | 308 | 0.027919 |
| x | TRBV2-9-2 | TRBJ2 | 432 | 0.039159 |
| x | TRBV2-9-2 | TRBJ3 | 601 | 0.054479 |
| x | TRBV2-9-2 | TRBJ4 | 541 | 0.04904 |
| x | TRBV2S3 | TRBJ2 | 1 | 9.06E-05 |
| x | TRBV3-1 | TRBJ1 | 1886 | 0.17096 |
| x | TRBV3-1 | TRBJ2 | 4634 | 0.420058 |
| x | TRBV3-1 | TRBJ3 | 4324 | 0.391957 |
| x | TRBV3-1 | TRBJ4 | 2735 | 0.247919 |
| x | TRBV3-2 | TRBJ1 | 7384 | 0.669337 |
| x | TRBV3-2 | TRBJ2 | 27523 | 2.494876 |
| x | TRBV3-2 | TRBJ3 | 11511 | 1.043437 |
| x | TRBV3-2 | TRBJ4 | 11282 | 1.022679 |
| x | TRBV3-3 | TRBJ3 | 1 | 9.06E-05 |
| x | TRBV3-4 | TRBJ1 | 32 | 0.002901 |
| x | TRBV3-4 | TRBJ2 | 128 | 0.011603 |
| x | TRBV3-4 | TRBJ3 | 85 | 0.007705 |
| x | TRBV3-4 | TRBJ4 | 64 | 0.005801 |
| x | TRBV3-5 | TRBJ1 | 505 | 0.045777 |
| x | TRBV3-5 | TRBJ2 | 580 | 0.052575 |
| x | TRBV3-5 | TRBJ3 | 116 | 0.010515 |
| x | TRBV3-5 | TRBJ4 | 316 | 0.028644 |
| x | TRBV3-6 | TRBJ1 | 3215 | 0.29143 |
| x | TRBV3-6 | TRBJ2 | 4337 | 0.393136 |
| x | TRBV3-6 | TRBJ3 | 3186 | 0.288801 |
| x | TRBV3-6 | TRBJ4 | 3671 | 0.332765 |
| x | TRBV3-8 | TRBJ1 | 3374 | 0.305843 |
| x | TRBV3-8 | TRBJ2 | 4706 | 0.426585 |
| x | TRBV3-8 | TRBJ3 | 3172 | 0.287532 |
| x | TRBV3-8 | TRBJ4 | 4477 | 0.405826 |
| x | TRBV4-1 | TRBJ1 | 884 | 0.080132 |
| x | TRBV4-1 | TRBJ2 | 1310 | 0.118748 |
| x | TRBV4-1 | TRBJ3 | 1321 | 0.119745 |
| x | TRBV4-1 | TRBJ4 | 1283 | 0.1163 |
| x | TRBV4-2 | TRBJ1 | 219 | 0.019852 |
| x | TRBV4-2 | TRBJ2 | 22 | 0.001994 |
| x | TRBV4-2 | TRBJ3 | 18 | 0.001632 |
| x | TRBV4-2 | TRBJ4 | 124 | 0.01124 |
| x | TRBV5-1 | TRBJ4 | 1 | 9.06E-05 |
| x | TRBV5-12 | TRBJ1 | 151 | 0.013688 |
| x | TRBV5-12 | TRBJ2 | 262 | 0.02375 |
| x | TRBV5-12 | TRBJ3 | 782 | 0.070886 |
| x | TRBV5-12 | TRBJ4 | 393 | 0.035624 |
| x | TRBV5-12-1 | TRBJ1 | 842 | 0.076325 |
| x | TRBV5-12-1 | TRBJ2 | 1609 | 0.145851 |
| x | TRBV5-12-1 | TRBJ3 | 1579 | 0.143132 |
| x | TRBV5-12-1 | TRBJ4 | 1273 | 0.115394 |
| x | TRBV5-12-2 | TRBJ1 | 517 | 0.046864 |
| x | TRBV5-12-2 | TRBJ2 | 817 | 0.074059 |
| x | TRBV5-12-2 | TRBJ3 | 904 | 0.081945 |
| x | TRBV5-12-2 | TRBJ4 | 1134 | 0.102794 |
| x | TRBV5-15 | TRBJ1 | 762 | 0.069073 |
| x | TRBV5-15 | TRBJ2 | 255 | 0.023115 |
| x | TRBV5-15 | TRBJ3 | 257 | 0.023296 |
| x | TRBV5-15 | TRBJ4 | 401 | 0.036349 |
| x | TRBV5-17 | TRBJ1 | 3497 | 0.316992 |
| x | TRBV5-17 | TRBJ2 | 7783 | 0.705505 |
| x | TRBV5-17 | TRBJ3 | 8436 | 0.764698 |
| x | TRBV5-17 | TRBJ4 | 10646 | 0.965027 |
| x | TRBV5-2 | TRBJ1 | 633 | 0.05738 |
| x | TRBV5-2 | TRBJ2 | 970 | 0.087928 |
| x | TRBV5-2 | TRBJ3 | 1025 | 0.092913 |
| x | TRBV5-2 | TRBJ4 | 905 | 0.082035 |
| x | TRBV5-4 | TRBJ1 | 5897 | 0.534545 |
| x | TRBV5-4 | TRBJ2 | 2953 | 0.26768 |
| x | TRBV5-4 | TRBJ3 | 3280 | 0.297322 |
| x | TRBV5-4 | TRBJ4 | 3139 | 0.284541 |
| x | TRBV5-6 | TRBJ1 | 3824 | 0.346634 |
| x | TRBV5-6 | TRBJ2 | 5638 | 0.511068 |
| x | TRBV5-6 | TRBJ3 | 3896 | 0.353161 |
| x | TRBV5-6 | TRBJ4 | 4910 | 0.445077 |
| x | TRBV5-6-2 | TRBJ1 | 23 | 0.002085 |
| x | TRBV5-6-2 | TRBJ2 | 32 | 0.002901 |
| x | TRBV5-6-2 | TRBJ3 | 28 | 0.002538 |
| x | TRBV5-6-2 | TRBJ4 | 36 | 0.003263 |
| x | TRBV5-6-3 | TRBJ1 | 3458 | 0.313457 |
| x | TRBV5-6-3 | TRBJ2 | 6753 | 0.612139 |
| x | TRBV5-6-3 | TRBJ3 | 5107 | 0.462934 |
| x | TRBV5-6-3 | TRBJ4 | 4470 | 0.405192 |
| x | TRBV5-6-4 | TRBJ1 | 165 | 0.014957 |
| x | TRBV5-6-4 | TRBJ2 | 330 | 0.029913 |
| x | TRBV5-6-4 | TRBJ3 | 309 | 0.02801 |
| x | TRBV5-6-4 | TRBJ4 | 298 | 0.027013 |
| x | TRBV5-6-5 | TRBJ1 | 485 | 0.043964 |
| x | TRBV5-6-5 | TRBJ2 | 1223 | 0.110861 |
| x | TRBV5-6-5 | TRBJ3 | 628 | 0.056926 |
| x | TRBV5-6-5 | TRBJ4 | 849 | 0.076959 |
| x | TRBV5-9 | TRBJ1 | 115 | 0.010424 |
| x | TRBV5-9 | TRBJ2 | 302 | 0.027375 |
| x | TRBV5-9 | TRBJ3 | 277 | 0.025109 |
| x | TRBV5-9 | TRBJ4 | 258 | 0.023387 |
| x | TRBV5-9-1 | TRBJ1 | 325 | 0.02946 |
| x | TRBV5-9-1 | TRBJ2 | 544 | 0.049312 |
| x | TRBV5-9-1 | TRBJ3 | 481 | 0.043601 |
| x | TRBV5-9-1 | TRBJ4 | 502 | 0.045505 |
| x | TRBV5-9-2 | TRBJ1 | 60 | 0.005439 |
| x | TRBV5-9-2 | TRBJ2 | 118 | 0.010696 |
| x | TRBV5-9-2 | TRBJ3 | 79 | 0.007161 |
| x | TRBV5-9-2 | TRBJ4 | 92 | 0.00834 |
| x | TRBV5-9-3 | TRBJ1 | 953 | 0.086387 |
| x | TRBV5-9-3 | TRBJ2 | 2299 | 0.208397 |
| x | TRBV5-9-3 | TRBJ3 | 1440 | 0.130532 |
| x | TRBV5-9-3 | TRBJ4 | 1599 | 0.144944 |
| x | TRBV5-9-4 | TRBJ1 | 623 | 0.056473 |
| x | TRBV5-9-4 | TRBJ2 | 1099 | 0.099621 |
| x | TRBV5-9-4 | TRBJ3 | 1274 | 0.115484 |
| x | TRBV5-9-4 | TRBJ4 | 1522 | 0.137965 |
| x | TRBV5S21 | TRBJ1 | 1 | 9.06E-05 |
| x | TRBV5S21 | TRBJ2 | 3 | 0.000272 |
| x | TRBV5S21 | TRBJ3 | 3 | 0.000272 |
| x | TRBV5S4 | TRBJ1 | 1 | 9.06E-05 |
| x | TRBV5S4 | TRBJ3 | 2 | 0.000181 |
| x | TRBV5S9 | TRBJ1 | 311 | 0.028191 |
| x | TRBV5S9 | TRBJ2 | 985 | 0.089287 |
| x | TRBV5S9 | TRBJ3 | 556 | 0.0504 |
| x | TRBV5S9 | TRBJ4 | 665 | 0.06028 |
| x | TRBV6-3 | TRBJ1 | 7 | 0.000635 |
| x | TRBV6-3 | TRBJ2 | 10 | 0.000906 |
| x | TRBV6-3 | TRBJ3 | 14 | 0.001269 |
| x | TRBV6-3 | TRBJ4 | 2 | 0.000181 |
| x | TRBV6-6 | TRBJ1 | 2421 | 0.219456 |
| x | TRBV6-6 | TRBJ2 | 4840 | 0.438731 |
| x | TRBV6-6 | TRBJ3 | 8846 | 0.801863 |
| x | TRBV6-6 | TRBJ4 | 3413 | 0.309378 |
| x | TRBV6-7 | TRBJ3 | 2 | 0.000181 |
| x | TRBV6-7 | TRBJ4 | 2 | 0.000181 |
| x | TRBV7-1 | TRBJ1 | 2979 | 0.270037 |
| x | TRBV7-1 | TRBJ2 | 637 | 0.057742 |
| x | TRBV7-1 | TRBJ3 | 1589 | 0.144038 |
| x | TRBV7-1 | TRBJ4 | 1501 | 0.136061 |
| x | TRBV7-3 | TRBJ1 | 3776 | 0.342283 |
| x | TRBV7-3 | TRBJ2 | 3028 | 0.274479 |
| x | TRBV7-3 | TRBJ3 | 3291 | 0.298319 |
| x | TRBV7-3 | TRBJ4 | 2967 | 0.26895 |
| x | TRBV7-4 | TRBJ3 | 4 | 0.000363 |
| x | TRBV7-4 | TRBJ4 | 1 | 9.06E-05 |
| x | TRBV8-11 | TRBJ1 | 24 | 0.002176 |
| x | TRBV8-11 | TRBJ2 | 40 | 0.003626 |
| x | TRBV8-11 | TRBJ3 | 71 | 0.006436 |
| x | TRBV8-11 | TRBJ4 | 53 | 0.004804 |
| x | TRBV8-12 | TRBJ1 | 66 | 0.005983 |
| x | TRBV8-12 | TRBJ2 | 163 | 0.014775 |
| x | TRBV8-12 | TRBJ3 | 218 | 0.019761 |
| x | TRBV8-12 | TRBJ4 | 209 | 0.018945 |
| x | TRBV8-13 | TRBJ1 | 2 | 0.000181 |
| x | TRBV8-13 | TRBJ2 | 2 | 0.000181 |
| x | TRBV8-13 | TRBJ3 | 4 | 0.000363 |
| x | TRBV8-13 | TRBJ4 | 13 | 0.001178 |
| x | TRBV8-2 | TRBJ1 | 19 | 0.001722 |
| x | TRBV8-2 | TRBJ2 | 21 | 0.001904 |
| x | TRBV8-2 | TRBJ3 | 37 | 0.003354 |
| x | TRBV8-2 | TRBJ4 | 16 | 0.00145 |
| x | TRBV8-4 | TRBJ1 | 2 | 0.000181 |
| x | TRBV8-4 | TRBJ2 | 1 | 9.06E-05 |
| x | TRBV8-4 | TRBJ3 | 5 | 0.000453 |
| x | TRBV8-4 | TRBJ4 | 5 | 0.000453 |
| x | TRBV8-5 | TRBJ1 | 35 | 0.003173 |
| x | TRBV8-5 | TRBJ2 | 19 | 0.001722 |
| x | TRBV8-5 | TRBJ3 | 11 | 0.000997 |
| x | TRBV8-5 | TRBJ4 | 44 | 0.003988 |
| x | TRBV8-8 | TRBJ1 | 108 | 0.00979 |
| x | TRBV8-8 | TRBJ2 | 373 | 0.033811 |
| x | TRBV8-8 | TRBJ3 | 203 | 0.018401 |
| x | TRBV8-8 | TRBJ4 | 320 | 0.029007 |
| x | TRBV8-8-1 | TRBJ1 | 2 | 0.000181 |
| x | TRBV8-8-1 | TRBJ3 | 3 | 0.000272 |
| x | TRBV8-8-1 | TRBJ4 | 1 | 9.06E-05 |

c: control group; b: Bartha-K61 strain infection group; x: XJ strain infection group.

**Supplementary Table 8** The top 100 CDR3 nt sequences in XJ strain infection group.

| CDR3 ID | CDR3 Sequence (nt) | Reads unique | Ratio |  |
| --- | --- | --- | --- | --- |
| Unique CDR3 nt_1 | GCTAACTGGGACTGGTACTTCGATGTC | 9263 | 0.84% |  |
| Unique CDR3 nt_2 | GCCAGAGATGGGTTTGCTTAC | 8938 | 0.81% |  |
| Unique CDR3 nt_3 | GCAAGAGATGGTAACTACTGGTACTTCGATGTC | 6476 | 0.59% |  |
| Unique CDR3 nt_4 | GCCAGAGATGGTTTTGCTTAC | 5439 | 0.49% |  |
| Unique CDR3 nt_5 | GCTAGATGGGACTGGTACTTCGATGTC | 4536 | 0.41% |  |
| Unique CDR3 nt_6 | ACTAGGGGGTACGTTGACTAC | 4161 | 0.38% |  |
| Unique CDR3 nt_7 | GCCAGACATAGGGATGCTATGGACTAC | 3522 | 0.32% |  |
| Unique CDR3 nt_8 | GCCAGAGACTGGGCTTAC | 3327 | 0.30% |  |
| Unique CDR3 nt_9 | GCAAGAGGGGTTTATTACTACGGTAGTAGCTACGCTATGGACTAC | 3210 | 0.29% |  |
| Unique CDR3 nt_10 | GCAAGAGAGGGTATTTATTACTACGGTAGTTACTATGCTATGGACTAC | 2996 | 0.27% |  |
| Unique CDR3 nt_11 | AAAAGATACGATTACTATGCTATGGACTAC | 2953 | 0.27% |  |
| Unique CDR3 nt_12 | GCAAGTTACTACGGTAGTAGCTACTTTGACTAC | 2911 | 0.26% |  |
| Unique CDR3 nt_13 | GCCAGAAACTGGGACTAC | 2822 | 0.26% |  |
| Unique CDR3 nt_14 | GCCAGAGATGATGGTTACTACTTCGATGTC | 2612 | 0.24% |  |
| Unique CDR3 nt_15 | GCCAGACAGCTCGGGCACTATGCTATGGACTAC | 2149 | 0.19% |  |
| Unique CDR3 nt_16 | GCAAGTTACTACGGTAGTAGCTACGCTATGGACTAC | 2104 | 0.19% |  |
| Unique CDR3 nt_17 | GCCAACTGGGGGTTTGCTTAC | 2061 | 0.19% |  |
| Unique CDR3 nt_18 | GCCAGAGTACCCGTCTACTATGGTTACGCCTACTGGTACTTCGATGTC | 1937 | 0.17% |  |
| Unique CDR3 nt_19 | GCCAGAACTGACTAC | 1901 | 0.17% |  |
| Unique CDR3 nt_20 | ACCACTGGGTTTGCTTAC | 1887 | 0.17% |  |
| Unique CDR3 nt_21 | GCAAGTTACTACGGTAGTAGCTATGCTATGGACTAC | 1841 | 0.17% |  |
| Unique CDR3 nt_22 | GCTAGGGGGTACTTCGATGTC | 1823 | 0.17% |  |
| Unique CDR3 nt_23 | GCAAGTCAGACAGCTCGGGCTACCTGGTTTGCTTAC | 1514 | 0.14% |  |
| Unique CDR3 nt_24 | GCAAGTTACTACGGTAGTAGCTACTGGTACTTCGATGTC | 1501 | 0.14% |  |
| Unique CDR3 nt_25 | GCAAGATTTTACTACGGTAGTAGCTACTGGTACTTCGATGTC | 1402 | 0.13% |  |
| Unique CDR3 nt_26 | GCTAGAGGTTACTTTGACTAC | 1394 | 0.13% |  |
| Unique CDR3 nt_27 | GCCAGAGACGGGTTTGCTTAC | 1347 | 0.12% |  |
| Unique CDR3 nt_28 | GCAAGATTATTTGACTAC | 1325 | 0.12% |  |
| Unique CDR3 nt_29 | GCAAGAGAGGACTATGGTAACTACTAC AREDYGNYY | 1303 | 0.12% |  |
| Unique CDR3 nt_30 | ACAAGAGAGGGATACTGGTACTTCGATGTC | 1291 | 0.12% |  |
| Unique CDR3 nt_31 | GCAAGGGGGGGGTTTGCTTAC | 1257 | 0.11% |  |
| Unique CDR3 nt_32 | AATGCATGTCAACTGTCCTGGTTTGCTTAC | 1239 | 0.11% |  |
| Unique CDR3 nt_33 | GCCAAATGGAGGGATGGTTACTACTATGCTATGGACTAC | 1236 | 0.11% |  |
| Unique CDR3 nt_34 | GCCACTGGGTTTGACTAC | 1230 | 0.11% |  |
| Unique CDR3 nt_35 | GCATATGATTACGACTATGCTATGGACTAC | 1228 | 0.11% |  |
| Unique CDR3 nt_36 | GCAAGACGCCTTTACTACGGCTATTACTATGCTATGGACTAC | 1214 | 0.11% |  |
| Unique CDR3 nt_37 | GCTAACTGGGACTAC | 1121 | 0.10% |  |
| Unique CDR3 nt_38 | GCCAGACATGATTACGCTATGGACTAC | 1112 | 0.10% |  |
| Unique CDR3 nt_39 | GCAAGATCCGCTTACGACTACTTTGACTAC | 1110 | 0.10% |  |
| Unique CDR3 nt_40 | GCAAGATATGGTAACTACGCTATGGACTAC | 1084 | 0.10% |  |
| Unique CDR3 nt_41 | GCCAGACATGATTATGCTATGGACTAC | 1037 | 0.09% |  |
| Unique CDR3 nt_42 | GCTAGAAGCGGGTTTAACTGGGACTACTTTGACTAC | 1031 | 0.09% |  |
| Unique CDR3 nt_43 | GCAACTGGGTTTGCTTAC | 1022 | 0.09% |  |
| Unique CDR3 nt_44 | GCAAGAAACTGGGACTACTTTGACTAC | 1000 | 0.09% |  |
| Unique CDR3 nt_45 | GCAAGATCTGATGGTTATGCTATGGACTAC | 996 | 0.09% |  |
| Unique CDR3 nt_46 | GCAAGAGCCTATGGTAACTTCTTTCTTTACTGGTACTTCGATGTC | 994 | 0.09% |  |
| Unique CDR3 nt_47 | GCTAGATCGGGCATTACTACGGGCTTTGACTAC | 989 | 0.09% |  |
| Unique CDR3 nt_48 | GCCAGAGAAGGACAGCTCGGGTTCCTTTATTACTATGCTATGGACTAC | 986 | 0.09% |  |
| Unique CDR3 nt_49 | GCAAGATACTACGGTAGTAGCTACTGGTACTTCGATGTC | 977 | 0.09% |  |
| Unique CDR3 nt_50 | GCAAGATACTATAGGTACGACTTTGACTAC | 956 | 0.09% |  |
| Unique CDR3 nt_51 | GCTAGCTGGGACTTTGACTAC | 901 | 0.08% |  |
| Unique CDR3 nt_52 | GCAAGAGAGGGGTTTGCTTAC | 890 | 0.08% |  |
| Unique CDR3 nt_53 | GCAAGAGCCTACTATGGTAACTACTTTGACTAC | 885 | 0.08% |  |
| Unique CDR3 nt_54 | GCTAGACGCTGGCTTGACTAC | 881 | 0.08% |  |
| Unique CDR3 nt_55 | GCCAGAGATCTGGGGAGTGCTTAC | 874 | 0.08% |  |
| Unique CDR3 nt_56 | GCAAGAGGCCTTTATTACTACGGTAGTTCCTATGCTATGGACTAC | 838 | 0.08% |  |
| Unique CDR3 nt_57 | GCTAGCGGTAGTAGCTACTTTGACTAC | 823 | 0.07% |  |
| Unique CDR3 nt_58 | GCAAGCTACTATGGTAACTACTATGCTATGGACTAC | 822 | 0.07% |  |
| Unique CDR3 nt_59 | GCAAGATCGTACGACTACTTTGACTAC | 782 | 0.07% |  |
| Unique CDR3 nt_60 | GCCAATGGTTACTTTGCTTAC | 781 | 0.07% |  |
| Unique CDR3 nt_61 | GCTAGAGGGTACTTTGACTAC | 779 | 0.07% |  |
| Unique CDR3 nt_62 | GCAAGAGGCTACGGTAACTACGGGTACTACTTTGACTAC | 756 | 0.07% |  |
| Unique CDR3 nt_63 | GCTAGAAGTACTGGGACGGTTGACTAC | 754 | 0.07% |  |
| Unique CDR3 nt_64 | GCTAGAGGGGACGGATCCCTCTTTGACTAC | 752 | 0.07% |  |
| Unique CDR3 nt_65 | GCAAGGTACGACTATGCTATGGACTAC | 750 | 0.07% |  |
| Unique CDR3 nt_66 | GATGGAGGGTACTTTGACTAC | 741 | 0.07% |  |
| Unique CDR3 nt_67 | GCCAGACACTACGGTAGTAGCTACTGGTACTTCGATGTC | 739 | 0.07% |  |
| Unique CDR3 nt_68 | AAAAGATCACTGGGACCGTTTGCTTAC | 733 | 0.07% |  |
| Unique CDR3 nt_69 | GCTAGAGGGTACCAGAGAGCCTACTTTGACTAC | 731 | 0.07% |  |
| Unique CDR3 nt_70 | GCAAGCCTTTACTACGGTAGTAGCTATTACTATGCTATGGACTAC | 710 | 0.06% |  |
| Unique CDR3 nt_71 | GCAAGAACGGCTACGGTACCCCTTGCTTAC | 709 | 0.06% |  |
| Unique CDR3 nt_72 | GCTAGAGGGGTCTACTATGGTAACGGGGGGTACTTCGATGTC | 699 | 0.06% |  |
| Unique CDR3 nt_73 | GCGAGGAACGCCTCCTTTGACTAC | 698 | 0.06% |  |
| Unique CDR3 nt_74 | GCAAGAGACCGTTACTACTTTGACTAC | 698 | 0.06% |  |
| Unique CDR3 nt_75 | GCAACCGTCTATGGTTACGACGCTATGGACTAC | 696 | 0.06% |  |
| Unique CDR3 nt_76 | GCTAGATCTAACTGGGACTAC | 688 | 0.06% |  |
| Unique CDR3 nt_77 | ACTAGAGGGTACTTCGATGTC | 668 | 0.06% |  |
| Unique CDR3 nt_78 | GCAAGAAGGGACTATGCTATGGACTAC | 668 | 0.06% |  |
| Unique CDR3 nt_79 | GCTAGACTGGGGGACTTTGCTTAC | 667 | 0.06% |  |
| Unique CDR3 nt_80 | GCAAGATATTACTACGGTAGTAGCTACTACTTTGACTAC | 663 | 0.06% |  |
| Unique CDR3 nt_81 | GCCACTGGTATGGACTAC | 661 | 0.06% |  |
| Unique CDR3 nt_82 | ACAAGAGGGTTTGCTTAC | 658 | 0.06% |  |
| Unique CDR3 nt_83 | GCATACGGTAGTAGCTACTGGTACTTCGATGTC | 656 | 0.06% |  |
| Unique CDR3 nt_84 | ACAAGAGAGAGCTATGGTAACTACGGGGAGTACTTCGATGTC | 655 | 0.06% |  |
| Unique CDR3 nt_85 | GCAAGAAACTATAGGTACGACGACTACTATGCTATGGACTAC | 643 | 0.06% |  |
| Unique CDR3 nt_86 | GCCAGAGAAAGGACGGGGTTTGCTTAC | 640 | 0.06% |  |
| Unique CDR3 nt_87 | GCCAGAAATTACTACGGTAGTAGCTACTATGCTATGGACTAC | 628 | 0.06% |  |
| Unique CDR3 nt_88 | GCAAGATATTACTACGGTAGTAGTGACTAC | 624 | 0.06% |  |
| Unique CDR3 nt_89 | GCAAGATTTATTACTACGGTAGTAGCTACGTACTACTTTGACTAC | 618 | 0.06% |  |
| Unique CDR3 nt_90 | GACTACGGTAGTAGCTACTTCGATGTC | 618 | 0.06% |  |
| Unique CDR3 nt_91 | GCTAGAGGGTACTTCGATGTC | 605 | 0.05% |  |
| Unique CDR3 nt_92 | GCAAGACATTACTACGGTCGCTATGCTATGGACTAC | 592 | 0.05% |  |
| Unique CDR3 nt_93 | GCAAGAGAGAACTGGTACTTCGATGTC | 587 | 0.05% |  |
| Unique CDR3 nt_94 | GCAAGAAGATATGGTAACTCTGCTATGGACTAC | 582 | 0.05% |  |
| Unique CDR3 nt_95 | GCCAGATATGGTTACGACGACGGGTTTGACTAC | 579 | 0.05% |  |
| Unique CDR3 nt_96 | GCAAGATATGGTAACTATGCTATGGACTAC | 574 | 0.05% |  |
| Unique CDR3 nt_97 | GCAAGACCTTCTACGGCTACCCGGTACTTCGATGTC | 569 | 0.05% |  |
| Unique CDR3 nt_98 | GCCAGAGACCCCTCTACGGTAGTAGCTACGTATGCTATGGACTAC | 568 | 0.05% |  |
| Unique CDR3 nt_99 | GCAATCTACTATGGTTACGAC | 567 | 0.05% |  |
| Unique CDR3 nt_100 | GCAAGGGATGATTACGACGGGGCCTGGTTTGCTTAC | 565 | 0.05% |  |

**Supplementary Table 9** The top 10 CDR3 nt sequences in Bartha-K61 strain infection group.

| CDR3 ID | CDR3 Sequence (nt) | Reads unique | Ratio |  |
| --- | --- | --- | --- | --- |
| Unique CDR3 nt_1 | GCTAACTGGGACTGGTACTTCGATGTC | 24027 | 1.71% |  |
| Unique CDR3 nt_2 | GCTAGAAACGGTCGGGCTACTTGGTTTGCTTAC | 14487 | 1.03% |  |
| Unique CDR3 nt_3 | GCTAGAGGGTACTTTGACTAC | 13149 | 0.94% |  |
| Unique CDR3 nt_4 | GCCAGAGATGGGTTTGCTTAC | 6859 | 0.49% |  |
| Unique CDR3 nt_5 | GCAAGTTACTACGGTAGTAGCTACTTTGACTAC | 6792 | 0.48% |  |
| Unique CDR3 nt_6 | GCAAGCTACGGTAGTAGCTACGTCTACTGGTACTTCGATGTC | 5308 | 0.38% |  |
| Unique CDR3 nt_7 | GCACTTAACTGGGACGCTGACTAC | 5171 | 0.37% |  |
| Unique CDR3 nt_8 | GCTAGGGGGTACTTCGATGTC | 3756 | 0.27% |  |
| Unique CDR3 nt_9 | GCAAGAAGCCACTACGGTAGTAGCTACGTAGGGGACTAC | 3735 | 0.27% |  |
| Unique CDR3 nt_10 | GCAAGAGATGGTAACTACTGGTACTTCGATGTC | 3733 | 0.27% |  |
| Unique CDR3 nt_11 | GCCAGTTCATTACTACGGCTACGAGGGGTTTACTATGCTATGGACTAC | 3612 | 0.26% |  |
| Unique CDR3 nt_12 | AAAAGATCGCAAACCTCCTACGACTAC | 3434 | 0.24% |  |
| Unique CDR3 nt_13 | GCCAGAAACTGGGACTAC | 3118 | 0.22% |  |
| Unique CDR3 nt_14 | ACCACTGGGTTTGCTTAC | 3056 | 0.22% |  |
| Unique CDR3 nt_15 | GCAAGATCCCTGTATGTATGGTTACGACGACGGTCCTATGCTATGGACTAC | 2911 | 0.21% |  |
| Unique CDR3 nt_16 | ACAATCCTTCCTTATTACTACGGTAGTAGCTACAGGTACTTCGATGTC | 2753 | 0.20% |  |
| Unique CDR3 nt_17 | GCAAGAGATTACCTTGCTTAC | 2697 | 0.19% |  |
| Unique CDR3 nt_18 | GCAAGATATGATGGTTACTACTGGTACTTCGATGTC | 2400 | 0.17% |  |
| Unique CDR3 nt_19 | GCCAGAACTGACTAC | 2229 | 0.16% |  |
| Unique CDR3 nt_20 | GCAAGAGGGGACGGGGCTTAC | 2199 | 0.16% |  |
| Unique CDR3 nt_21 | GCTAGATCACGACCGTACTATGCTATGGACTAC | 2189 | 0.16% |  |
| Unique CDR3 nt_22 | GCCAGAGATGGTTTTGCTTAC | 2172 | 0.15% |  |
| Unique CDR3 nt_23 | GCACTTAACTGGGACGCTGACTTC | 2149 | 0.15% |  |
| Unique CDR3 nt_24 | GCAAGAGGGGATGGTTACCCC | 2140 | 0.15% |  |
| Unique CDR3 nt_25 | GCCAGTGCCTACTATGGTATCTATTACTATGCTATGGACTAC | 2098 | 0.15% |  |
| Unique CDR3 nt_26 | GCCCAATCTTACTACGGCTACGGGGTTTACTATGCTATGGACTAC | 2025 | 0.14% |  |
| Unique CDR3 nt_27 | ACAAGATTTTACTACGGTAGTAGCTACTACTTTGACTAC | 1952 | 0.14% |  |
| Unique CDR3 nt_28 | GCCAGAGACTGGGCTTAC | 1947 | 0.14% |  |
| Unique CDR3 nt_29 | GCAACGTCCTCCATATATTACTATGCTATGGACTAC | 1939 | 0.14% |  |
| Unique CDR3 nt_30 | TACTACGGTAGTAGCTACTGGTACTTCGATGTC | 1913 | 0.14% |  |
| Unique CDR3 nt_31 | GCAAGGGTGGTTACTACCTATTACTATGCTATGGACTAC | 1823 | 0.13% |  |
| Unique CDR3 nt_32 | GCTAGATCTGGCTTTATTACTACGGTAGTAGCTGTTGACTAC | 1790 | 0.13% |  |
| Unique CDR3 nt_33 | GCAAGATGGTCTACTACGGCCTTCGATGTC | 1706 | 0.12% |  |
| Unique CDR3 nt_34 | GCTAGAATGAGGGGGTTTGCTTAC | 1673 | 0.12% |  |
| Unique CDR3 nt_35 | GCCAGTTCCTCTTATGGTAACCTTTATTACTATGCTATGGACTAC | 1651 | 0.12% |  |
| Unique CDR3 nt_36 | GACTATGGGAACTCTTTTGCTTAC | 1641 | 0.12% |  |
| Unique CDR3 nt_37 | GCTAGATGGGACTGGTACTTCGATGTC | 1639 | 0.12% |  |
| Unique CDR3 nt_38 | GCCAGACACTACGGCTATGCTATGGACTAC | 1551 | 0.12% |  |
| Unique CDR3 nt_39 | GCAAGTGGGTTTGCTTAC | 1527 | 0.11% |  |
| Unique CDR3 nt_40 | GCAAGTTACTACGGTAGTAGCTATGCTATGGACTAC | 1506 | 0.11% |  |
| Unique CDR3 nt_41 | GCAAGACATGATTACGCCTGGTTTGCTTAC | 1496 | 0.11% |  |
| Unique CDR3 nt_42 | GCAAGAAGGGACTATGGTTACGATGGCTATGCTATGGACTAC | 1486 | 0.11% |  |
| Unique CDR3 nt_43 | GCAAGGGGGGGGTTTGCTTAC | 1464 | 0.10% |  |
| Unique CDR3 nt_44 | GCCAGACACTACGGTAGTAGCTACTGGTACTTCGATGTC | 1428 | 0.10% |  |
| Unique CDR3 nt_45 | GCTAGAACTTACTACGGTAGTAGCTTTGACTAC | 1366 | 0.10% |  |
| Unique CDR3 nt_46 | GCTAGTTATTACTACGGTAGTAGCTACGAGGGGTTTGCTTAC | 1339 | 0.10% |  |
| Unique CDR3 nt_47 | GCAAGATTTTACTACGGTAGTAGCTACTGGTACTTCGATGTC | 1327 | 0.09% |  |
| Unique CDR3 nt_48 | GCATATTACTACGGTAGTTTTGTC | 1306 | 0.09% |  |
| Unique CDR3 nt_49 | GCCAGAGGAGGATTTGCTTAC | 1282 | 0.09% |  |
| Unique CDR3 nt_50 | ACAAGAGATGGCTTTGCTTAC | 1279 | 0.09% |  |
| Unique CDR3 nt_51 | GCTAGATGGGGTACTACGGTTCCGTTTGCTTAC | 1262 | 0.09% |  |
| Unique CDR3 nt_52 | AATGCCCCCTTCTACTATGGTTACGGCCCCTATACTATGGACTAC | 1258 | 0.09% |  |
| Unique CDR3 nt_53 | ACAAGATCCTATTACTACGGTAGTAGCTACGCTATGGACTAC | 1248 | 0.09% |  |
| Unique CDR3 nt_54 | GCTAGACGAGCTAACTGGGCGCATTACTATGCTATGGACTAC | 1245 | 0.09% |  |
| Unique CDR3 nt_55 | ACCAGGCCCTGGGAAGCTTAC | 1225 | 0.09% |  |
| Unique CDR3 nt_56 | GCAAGAGCGTATTACTACGGTAGTCTTGACTAC | 1225 | 0.09% |  |
| Unique CDR3 nt_57 | GCTAGTGGGGATCCCTCCTGGTTTGCTTAC | 1217 | 0.09% |  |
| Unique CDR3 nt_58 | GCTAGATCGACTGGGACAGTTGACTAC | 1187 | 0.08% |  |
| Unique CDR3 nt_59 | GCTAGAAAGACGAATAGGTACGACGTCTATGCTATGGACTAC | 1141 | 0.08% |  |
| Unique CDR3 nt_60 | GCAAGAAGGGGGTACTACGGTAGTAGCTACGAGTGGTACTTCGATGTC | 1130 | 0.08% |  |
| Unique CDR3 nt_61 | GCAAGACATGGTTACGACTGGTTTGCTTAC | 1089 | 0.08% |  |
| Unique CDR3 nt_62 | GCAAGGTCGGACTACGGTAGTACCTGGTTTGCTTAC | 1071 | 0.08% |  |
| Unique CDR3 nt_63 | GCAAGATGGCGGGATTACTATGCTATGGACTAC | 1064 | 0.08% |  |
| Unique CDR3 nt_64 | GCAAGTGGTAACTACTGGTACTTCGATGTC | 1061 | 0.08% |  |
| Unique CDR3 nt_65 | GCAAGATGCGTCTATGGTTACGACGGCGACTAC | 1048 | 0.07% |  |
| Unique CDR3 nt_66 | GCAAGGCACTACGGGTACTACTTTGACTAC | 1035 | 0.07% |  |
| Unique CDR3 nt_67 | GCAAGATTGGCCTACTACGGCTACGCTATGGACTAC | 1030 | 0.07% |  |
| Unique CDR3 nt_68 | GCCAACTGGGGGTTTGCTTAC | 1023 | 0.07% |  |
| Unique CDR3 nt_69 | GCTGCTATCTACTATCCTGACTCCGACGGCTAC | 1022 | 0.07% |  |
| Unique CDR3 nt_70 | GCCAGAGATCTTTATTACTACGGTAGTAGCTATGCTATGGACTAC | 1013 | 0.07% |  |
| Unique CDR3 nt_71 | GCAAGGGATTACTACGGTAGTAGCTACTGGTACTTCGATGTC | 1012 | 0.07% |  |
| Unique CDR3 nt_72 | GCTAACTGGGACTAC | 1012 | 0.07% |  |
| Unique CDR3 nt_73 | GCTAGAGGGTACGCGGTCTAC | 1000 | 0.07% |  |
| Unique CDR3 nt_74 | GCAAGAGAGGGGTTTGCTTAC | 999 | 0.07% |  |
| Unique CDR3 nt_75 | GCAAGACAGCTAATCTATGCTATGGACTAC | 988 | 0.07% |  |
| Unique CDR3 nt_76 | GCAAGATCCTACGGTTACTATGGTATGGACTAC | 979 | 0.07% |  |
| Unique CDR3 nt_77 | GCAACTATGGTAACTACCTGGTTTGCTTAC | 972 | 0.07% |  |
| Unique CDR3 nt_78 | GCAAGGGGGGGTAACTACCCTCTCTTTGCTTAC | 958 | 0.07% |  |
| Unique CDR3 nt_79 | GCCAGACAGCTCGGGTTTGCTTAC | 949 | 0.07% |  |
| Unique CDR3 nt_80 | GCAAGAGCCCTCTACTATGATTACGACTTTGACTAC | 948 | 0.07% |  |
| Unique CDR3 nt_81 | GCTAGAAGGGGAGGGCCATATTACTATGCTATGGACTAC | 925 | 0.07% |  |
| Unique CDR3 nt_82 | GCAAGATCTCAACTGGGACACTTTGACTAC | 921 | 0.07% |  |
| Unique CDR3 nt_83 | GCTAGAAAGGCGGGTGATGATTACTATTTTGACTAC | 921 | 0.07% |  |
| Unique CDR3 nt_84 | GCTAGGGGGTACGGTAGTAGCTGGTTTGCTTAC | 919 | 0.07% |  |
| Unique CDR3 nt_85 | GGAAGACACGGCTACAGTGACTATGCTATGGACTAC | 903 | 0.06% |  |
| Unique CDR3 nt_86 | GCCAGACGGGGTGATGGTTACTACCTTGACTAC | 891 | 0.06% |  |
| Unique CDR3 nt_87 | GCAAGTTACTACGGTAGTAGCTACGCTATGGACTAC | 884 | 0.06% |  |
| Unique CDR3 nt_88 | GCAAGAAATGGGGTACGGCTAGGTTACTTTGACTAC | 870 | 0.06% |  |
| Unique CDR3 nt_89 | GCCAGCCCTGACAGCTCGGGCTACGTGGCTATGGACTAC | 865 | 0.06% |  |
| Unique CDR3 nt_90 | GCTAAGGGACTGGACTGGTACTTCGATGTC | 854 | 0.06% |  |
| Unique CDR3 nt_91 | ACCAGGATTAACTGGGGAGCTATGGACTAC | 847 | 0.05% |  |
| Unique CDR3 nt_92 | GCTAGAAGGGATGGTAACTACGATTACTATGCTATGGACTAC | 846 | 0.06% |  |
| Unique CDR3 nt_93 | GCCCCCCAATATTTTGATGGTTACTACGGATTTGCTTAC | 846 | 0.06% |  |
| Unique CDR3 nt_94 | GCCAAAGGGTACGACGGGATTTACTATGCTATGGACTAC | 846 | 0.06% |  |
| Unique CDR3 nt_95 | GCAAGGGCCGCGGCCTGGTTTGCTTAC | 841 | 0.06% |  |
| Unique CDR3 nt_96 | GCAAGATACTACGGTAGTAGCTACTACTTTGACTAC | 832 | 0.06% |  |
| Unique CDR3 nt_97 | GCTAGATGGTTACGACGGGGGAAC | 812 | 0.06% |  |
| Unique CDR3 nt_98 | GCAACATCCTATTACTACGGTAGTAGCAATTACTACTTTGACTAC | 805 | 0.06% |  |
| Unique CDR3 nt_99 | GTTCTTTATTACTACGGTAGTAGCTATGACTGGTACTTCGATGTC | 799 | 0.06% |  |
| Unique CDR3 nt_100 | GCAAGAAGGGTCCTGAATTACTATGCTATGGACTAC | 793 | 0.06% |  |

**Supplementary Table 10** The top 100 CDR3 nt sequences in control group.

| CDR3 ID | CDR3 Sequence (nt) | Reads unique | Ratio |  |
| --- | --- | --- | --- | --- |
| Unique CDR3 nt_1 | GCTAGATGGGACTGGTACTTCGATGTC | 46482 | 4.37% |  |
| Unique CDR3 nt_2 | GCTAACTGGGACTGGTACTTCGATGTC | 14734 | 1.39% |  |
| Unique CDR3 nt_3 | GCAAGAGGGGATTCCTACTAC | 8126 | 0.76% |  |
| Unique CDR3 nt_4 | GCTAGAGGGTACTTTGACTAC | 5596 | 0.53% |  |
| Unique CDR3 nt_5 | GCAAGGCCCTACGGTAGTACCTACGGCTACTACTTTGACTAC | 5105 | 0.48% |  |
| Unique CDR3 nt_6 | GCCAGAGATGGGTTTGCTTAC | 5001 | 0.47% |  |
| Unique CDR3 nt_7 | GCTAGAGAGGGTGGGGGTTACTATGGTGCTATGGACTAC | 4355 | 0.41% |  |
| Unique CDR3 nt_8 | GCGATCTACTATGGTTACGAC | 3869 | 0.36% |  |
| Unique CDR3 nt_9 | GCAAGTGCTATGGACTAC | 3049 | 0.29% |  |
| Unique CDR3 nt_10 | GCCAGAAACTGGGACTAC | 2892 | 0.27% |  |
| Unique CDR3 nt_11 | GCAAGTTACTACGGTAGTAGCTATGCTATGGACTAC | 2892 | 0.27% |  |
| Unique CDR3 nt_12 | GCAAGGCCCTACGGTAGTAGCTACGGCTACTACTTTGACTAC | 2846 | 0.27% |  |
| Unique CDR3 nt_13 | GCAAGAGATGGTAACTACTGGTACTTCGATGTC | 2500 | 0.24% |  |
| Unique CDR3 nt_14 | GCAAGAGGAGAGGCAATTTATTACTACGGTAGTAGCGGAGATTACTATGCTATGGACTAC | 2479 | 0.23% |  |
| Unique CDR3 nt_15 | GCAAGATCAGGTTACTTCTATGCTATGGACTAC | 2200 | 0.21% |  |
| Unique CDR3 nt_16 | TACTACGGTAGTAGCTACTGGTACTTCGATGTC | 2195 | 0.21% |  |
| Unique CDR3 nt_17 | ACAAGAAAGGGATCGTCCCGAACTCAATATGATTACGACGCCTGGTTTGCTTAC | 2035 | 0.19% |  |
| Unique CDR3 nt_18 | GCAAGAGGGCACTATGATTACGACTAC | 1963 | 0.18% |  |
| Unique CDR3 nt_19 | GCAAGGGGGGGGTTTGCTTAC | 1852 | 0.17% |  |
| Unique CDR3 nt_20 | GCAAGATCCCTTATTTATGCTATGGACTAC | 1834 | 0.17% |  |
| Unique CDR3 nt_21 | ACAAGATGGTTACTACGTGCTATGGACTAC | 1818 | 0.17% |  |
| Unique CDR3 nt_22 | GCTATGATTACTGCTATGGACTAC | 1701 | 0.16% |  |
| Unique CDR3 nt_23 | GCTAGAGAGGACTTGTGGTACTTCGATGTC | 1497 | 0.14% |  |
| Unique CDR3 nt_24 | GCAAAGTATGGTAACTACTACTTTGACTAC | 1464 | 0.14% |  |
| Unique CDR3 nt_25 | GCAAGGGGTGGGTTTGCTTAC | 1454 | 0.14% |  |
| Unique CDR3 nt_26 | GCCAGAACTGACTAC | 1452 | 0.14% |  |
| Unique CDR3 nt_27 | GCAAGAATTTACTACGGTAGTAGCTACTGGTACTTCGATGTC | 1447 | 0.14% |  |
| Unique CDR3 nt_28 | GCAAGATCTTACTACGATGGTAGCTGGTACTTCGATGTC | 1434 | 0.13% |  |
| Unique CDR3 nt_29 | GCAAGATACTACGGTAGTAGCTACTACTTTGACTAC | 1421 | 0.13% |  |
| Unique CDR3 nt_30 | GCAAGTAGTATGGACTAC | 1408 | 0.13% |  |
| Unique CDR3 nt_31 | GCAAGACATAGGTACGTTGACTAC | 1405 | 0.13% |  |
| Unique CDR3 nt_32 | GCAAGTAGGTACGACTGGTACTTCGATGTC | 1362 | 0.13% |  |
| Unique CDR3 nt_33 | GCAAGGGAGGATGGTTACTACTGGTTTGCTTAC | 1283 | 0.12% |  |
| Unique CDR3 nt_34 | ACTTATGATTACGAAACCTGGTTTGCTTAC | 1275 | 0.12% |  |
| Unique CDR3 nt_35 | GCAAGAAACGGCTACGTTGGTTACTACTTTGACTAC | 1208 | 0.11% |  |
| Unique CDR3 nt_36 | GCAAGAAGACTTCATTACTACGGCTACGTGGATTACTATGCTATGGACTAC | 1206 | 0.11% |  |
| Unique CDR3 nt_37 | GCAAGGCTAACTGGGACGAACTAC | 1156 | 0.11% |  |
| Unique CDR3 nt_38 | ACAAGATCGGGGTATGGTAACTACTTTGACTAC | 1131 | 0.11% |  |
| Unique CDR3 nt_39 | GCAAGACACGGCTATGCTATGGACTAC | 1130 | 0.11% |  |
| Unique CDR3 nt_40 | GCATACGACTATGCTATGGACTAC | 1103 | 0.10% |  |
| Unique CDR3 nt_41 | GCAACTGGGTTTGCTTAC | 1098 | 0.10% |  |
| Unique CDR3 nt_42 | GCAAGAAGGAGGTACTACCCCTGGTTTGCTTAC | 1096 | 0.10% |  |
| Unique CDR3 nt_43 | GCTAACTGGGACGCCTCTTTTGCTTAC | 1094 | 0.10% |  |
| Unique CDR3 nt_44 | ACCACTGGGTTTGCTTAC | 1040 | 0.10% |  |
| Unique CDR3 nt_45 | GCAAGACAAGGGTTTGACTAC | 1023 | 0.10% |  |
| Unique CDR3 nt_46 | GCAAGAGATGGTTACTACTTTGACTAC | 1003 | 0.09% |  |
| Unique CDR3 nt_47 | GCTAACTGGGACTAC | 976 | 0.09% |  |
| Unique CDR3 nt_48 | GCAAGATGGGACTAC | 964 | 0.09% |  |
| Unique CDR3 nt_49 | TATGGTAACTCGTTTGCTTAC | 935 | 0.09% |  |
| Unique CDR3 nt_50 | GCTAGATCGGGTTACTACGGTGACTAC | 922 | 0.09% |  |
| Unique CDR3 nt_51 | GCCAGGTACGACTACTATGCTATGGACTAC | 919 | 0.09% |  |
| Unique CDR3 nt_52 | GCCAGGGACTGGGCTTAC | 910 | 0.09% |  |
| Unique CDR3 nt_53 | GCCAAAAATGATTACGACCTATATTACTATGCTATGGACTAC | 910 | 0.09% |  |
| Unique CDR3 nt_54 | GCAAGATACTATAGGTACGACGCTATGGACTAC | 878 | 0.08% |  |
| Unique CDR3 nt_55 | GCCCGTGATGGTTACTACTTTGACTAC | 869 | 0.08% |  |
| Unique CDR3 nt_56 | GCAAGAGGGTACTTCGGTAGTACCTATTGGTACTTCGATGTC | 852 | 0.08% |  |
| Unique CDR3 nt_57 | GCCTACGGTAGTAGCTACTGGTACTTCGATGTC | 848 | 0.08% |  |
| Unique CDR3 nt_58 | GCAAGCTACTATGGTAACTACTATGCTATGGACTAC | 842 | 0.08% |  |
| Unique CDR3 nt_59 | GCAAGATTTTACTACGGTAGTAGCTACTGGTACTTCGATGTC | 803 | 0.08% |  |
| Unique CDR3 nt_60 | GCCAGAAATTGGGACTAC | 799 | 0.08% |  |
| Unique CDR3 nt_61 | GCCAACTGGGGGTTTGCTTAC | 798 | 0.08% |  |
| Unique CDR3 nt_62 | GCAAGAAAATGGTTACTACGTACGGCGTTTTCTTACTATGCTATGGACTAC | 786 | 0.07% |  |
| Unique CDR3 nt_63 | GCTAGAAGGGCTAATCGGGGGTACTTCGATGTC | 783 | 0.07% |  |
| Unique CDR3 nt_64 | ACAACTGGGTTTGCTTAC | 780 | 0.07% |  |
| Unique CDR3 nt_65 | GCAAGGCCCTACGGTAGTACCTACGGCTACTTCTTTGACTAC | 780 | 0.07% |  |
| Unique CDR3 nt_66 | GCAAGTGGTTACGACTTTGACTAC | 770 | 0.07% |  |
| Unique CDR3 nt_67 | GCCAGAGATCCGCGGTTTATTACTACGGTAGTAGACTACTATGCTATGGACTAC | 765 | 0.07% |  |
| Unique CDR3 nt_68 | GCAAGATCGGGACTGGGACTGTTTGACTAC | 762 | 0.07% |  |
| Unique CDR3 nt_69 | GCAAGAGGGGGGGATTACGACGTCCATTATGCTATGGACTAC | 722 | 0.07% |  |
| Unique CDR3 nt_70 | GCCAAAAATGATTACGACGTCGTTTACTATGCTATGGACTAC | 718 | 0.07% |  |
| Unique CDR3 nt_71 | GCCAGAATTACTACGGCTACTGCTTAC | 701 | 0.07% |  |
| Unique CDR3 nt_72 | ACGGGCGGTAACTACGAGTGGTTTGCTTAC | 696 | 0.07% |  |
| Unique CDR3 nt_73 | GCAAGTTACTACGGTAGTAGCTACTTTGACTAC | 694 | 0.07% |  |
| Unique CDR3 nt_74 | GCAAGAGAGGGGTTTGCTTAC | 691 | 0.06% |  |
| Unique CDR3 nt_75 | GCAAGAGATAACTGGGACTGGTACTTCGATGTC | 690 | 0.06% |  |
| Unique CDR3 nt_76 | ACAAGATATCGTTACTACGGTAGTAACTACGTCTGGTACTTCGATGTC | 672 | 0.06% |  |
| Unique CDR3 nt_77 | GCAAGCTACGGTAGTAGCTACTGGTACTTCGATGTC | 661 | 0.06% |  |
| Unique CDR3 nt_78 | GCAAGAGGGGATGGTTACTAC | 661 | 0.06% |  |
| Unique CDR3 nt_79 | GCAAGCTACTACGGTAGTAGCTACGCTATGGACTAC | 657 | 0.06% |  |
| Unique CDR3 nt_80 | GCAAGACTCTATGATGGTTACTACTGGTTTGCTTAC | 645 | 0.06% |  |
| Unique CDR3 nt_81 | GCAAGAGAGGGTGATGGTTACTACTGGTTTGCTTAC | 640 | 0.06% |  |
| Unique CDR3 nt_82 | GCCAGAGATTGGGCTTAC | 629 | 0.06% |  |
| Unique CDR3 nt_83 | GCAAGGGATTACTACGGTAGTAGCTACTGGTACTTCGATGTC | 623 | 0.06% |  |
| Unique CDR3 nt_84 | GCAAGTTACTACGGTAGTAGCTACGCTATGGACTAC | 590 | 0.06% |  |
| Unique CDR3 nt_85 | GCAAGTCCTTACTACGGTGACTAC | 586 | 0.06% |  |
| Unique CDR3 nt_86 | ACATACGGTAGTAGTTACTATGCTATGGACTAC | 584 | 0.05% |  |
| Unique CDR3 nt_87 | GCAAGACAGGGCTATGACTAC | 577 | 0.05% |  |
| Unique CDR3 nt_88 | GCAAGACAGAGGTACGACTATGCTATGGACTAC | 574 | 0.05% |  |
| Unique CDR3 nt_89 | GCCAGAGATGGTTACTATGCTATGGACTAC | 572 | 0.05% |  |
| Unique CDR3 nt_90 | GCTAGCTGGGACTTTGACTAC | 567 | 0.05% |  |
| Unique CDR3 nt_91 | GCAAGAGATGATTACGACTGGTACTTCGATGTC | 565 | 0.05% |  |
| Unique CDR3 nt_92 | GCAAGAAGGGATTACTATGCTATGGACTAC | 563 | 0.05% |  |
| Unique CDR3 nt_93 | GCAAGACAAGGGTACGACTAC | 557 | 0.05% |  |
| Unique CDR3 nt_94 | GGTAACTATGCTATGGACTAC | 550 | 0.05% |  |
| Unique CDR3 nt_95 | GCTAGAAGGCTCTACTATGGTAACCACGTCTTTGACTAC | 546 | 0.05% |  |
| Unique CDR3 nt_96 | GCAAGAGATTACTACGGTAGTAGCTACTTTGACTAC | 541 | 0.05% |  |
| Unique CDR3 nt_97 | GCAAGTAGTAGCTACGCTTAC | 812 | 0.06% |  |
| Unique CDR3 nt_98 | GCTTGGGATTATGCTATGGACTAC | 536 | 0.05% |  |
| Unique CDR3 nt_99 | GCAAGATCTGTCGGGAACTTTGACTAC | 533 | 0.05% |  |
| Unique CDR3 nt_100 | GCTAGGGGGTACTTTGACTAC | 531 | 0.05% |  |

**Supplementary Table 14** The top 100 CDR3 aa sequences in XJ strain infection group.

| CDR3 ID | CDR3 Sequence (aa) | Percent |
| --- | --- | --- |
| Unique CDR3 aa_1 | YQGGTFDY | 9.97116520317156e-06 |
| Unique CDR3 aa_2 | VTQTMINWYFDV | 9.97116520317156e-06 |
| Unique CDR3 aa_3 | VTLSYAMDY | 9.97116520317156e-06 |
| Unique CDR3 aa_4 | VTKTARATPLAY | 9.97116520317156e-06 |
| Unique CDR3 aa_5 | VSYRSYAMDY | 9.97116520317156e-06 |
| Unique CDR3 aa_6 | VSYGSSFAY | 9.97116520317156e-06 |
| Unique CDR3 aa_7 | VSLNWDEAY | 9.97116520317156e-06 |
| Unique CDR3 aa_8 | VRYGYDDAMDY | 9.97116520317156e-06 |
| Unique CDR3 aa_9 | VRWGGNSRGFDY | 9.97116520317156e-06 |
| Unique CDR3 aa_10 | VRTGYDPFAY | 9.97116520317156e-06 |
| Unique CDR3 aa_11 | VRTAY | 9.97116520317156e-06 |
| Unique CDR3 aa_12 | VRSLRN | 9.97116520317156e-06 |
| Unique CDR3 aa_13 | VRRRLDAMDY | 9.97116520317156e-06 |
| Unique CDR3 aa_14 | VRRGNYEAMDY | 9.97116520317156e-06 |
| Unique CDR3 aa_15 | VRRDYYGSSGAMDY | 9.97116520317156e-06 |
| Unique CDR3 aa_16 | VRRDY | 9.97116520317156e-06 |
| Unique CDR3 aa_17 | VRRAMDY | 9.97116520317156e-06 |
| Unique CDR3 aa_18 | VRQIATMDY | 9.97116520317156e-06 |
| Unique CDR3 aa_19 | VRQGDGYLFAY | 9.97116520317156e-06 |
| Unique CDR3 aa_20 | VRLAY | 9.97116520317156e-06 |
| Unique CDR3 aa_21 | VRHYYGSSYAMDY | 9.97116520317156e-06 |
| Unique CDR3 aa_22 | VRHYYGMDY | 9.97116520317156e-06 |
| Unique CDR3 aa_23 | VRHVDY | 9.97116520317156e-06 |
| Unique CDR3 aa_24 | VRHRYDAY | 9.97116520317156e-06 |
| Unique CDR3 aa_25 | VRHDGNYWFAY | 9.97116520317156e-06 |
| Unique CDR3 aa_26 | VRHDAYDGGPWFAY | 9.97116520317156e-06 |
| Unique CDR3 aa_27 | VRGTVSFDY | 9.97116520317156e-06 |
| Unique CDR3 aa_28 | VRGPYGSLAY | 9.97116520317156e-06 |
| Unique CDR3 aa_29 | VRGDYYYGSSYYAMDY | 9.97116520317156e-06 |
| Unique CDR3 aa_30 | VRERYRYDGKGAMDY | 9.97116520317156e-06 |
| Unique CDR3 aa_31 | VRERYRHAMDY | 9.97116520317156e-06 |
| Unique CDR3 aa_32 | VREDYGNSWYFDV | 9.97116520317156e-06 |
| Unique CDR3 aa_33 | VREDRYVYFDY | 9.97116520317156e-06 |
| Unique CDR3 aa_34 | VRDYYYGSSYYWYFDV | 9.97116520317156e-06 |
| Unique CDR3 aa_35 | VRDVRPYWYFDV | 9.97116520317156e-06 |
| Unique CDR3 aa_36 | VRDRYGAY | 9.97116520317156e-06 |
| Unique CDR3 aa_37 | VRDHPYYFDY | 9.97116520317156e-06 |
| Unique CDR3 aa_38 | VRDGNSSPY | 9.97116520317156e-06 |
| Unique CDR3 aa_39 | VRDGNFDY | 9.97116520317156e-06 |
| Unique CDR3 aa_40 | VRDGDYWFAY | 9.97116520317156e-06 |
| Unique CDR3 aa_41 | VRDDGNWFAY | 9.97116520317156e-06 |
| Unique CDR3 aa_42 | VPHYYGSERIDYYAMDY | 9.97116520317156e-06 |
| Unique CDR3 aa_43 | VHYYGYAMDY | 9.97116520317156e-06 |
| Unique CDR3 aa_44 | VGSFAY | 9.97116520317156e-06 |
| Unique CDR3 aa_45 | VGFAY | 9.97116520317156e-06 |
| Unique CDR3 aa_46 | VAMITTGFAY | 9.97116520317156e-06 |
| Unique CDR3 aa_47 | TYYNWYFDV | 9.97116520317156e-06 |
| Unique CDR3 aa_48 | TVSTMITTWGAMDY | 9.97116520317156e-06 |
| Unique CDR3 aa_49 | TVLYGIFAY | 9.97116520317156e-06 |
| Unique CDR3 aa_50 | TVLNAFGY | 9.97116520317156e-06 |
| Unique CDR3 aa_51 | TTEGY | 9.97116520317156e-06 |
| Unique CDR3 aa_52 | TTATY | 9.97116520317156e-06 |
| Unique CDR3 aa_53 | TSYYYGSSSLDY | 9.97116520317156e-06 |
| Unique CDR3 aa_54 | TSTGQGGFDY | 9.97116520317156e-06 |
| Unique CDR3 aa_55 | TSQYGIFAY | 9.97116520317156e-06 |
| Unique CDR3 aa_56 | TSGNYY | 9.97116520317156e-06 |
| Unique CDR3 aa_57 | TRYYGSNYYAMDY | 9.97116520317156e-06 |
| Unique CDR3 aa_58 | TRYGYGDWFAY | 9.97116520317156e-06 |
| Unique CDR3 aa_59 | TRWPIYDAYYVGYHYTMDY | 9.97116520317156e-06 |
| Unique CDR3 aa_60 | TRWGYYAYYAMDY | 9.97116520317156e-06 |
| Unique CDR3 aa_61 | TRWGNYPWFAY | 9.97116520317156e-06 |
| Unique CDR3 aa_62 | TRVLWLRAMDY | 9.97116520317156e-06 |
| Unique CDR3 aa_63 | TRTPVGDY | 9.97116520317156e-06 |
| Unique CDR3 aa_64 | TRTGTGYFDV | 9.97116520317156e-06 |
| Unique CDR3 aa_65 | TRSTTDWYFDV | 9.97116520317156e-06 |
| Unique CDR3 aa_66 | TRSSDP | 9.97116520317156e-06 |
| Unique CDR3 aa_67 | TRSRNDGYYWFAY | 9.97116520317156e-06 |
| Unique CDR3 aa_68 | TRSNWVLDY | 9.97116520317156e-06 |
| Unique CDR3 aa_69 | TRSHLYYGSSYYAMDY | 9.97116520317156e-06 |
| Unique CDR3 aa_70 | TRSGVYYYGSSYEAYAMDY | 9.97116520317156e-06 |
| Unique CDR3 aa_71 | TRSGVITVDY | 9.97116520317156e-06 |
| Unique CDR3 aa_72 | TRSGRYDEGGY | 9.97116520317156e-06 |
| Unique CDR3 aa_73 | TRSGNWDGAMDY | 9.97116520317156e-06 |
| Unique CDR3 aa_74 | TRSGHFYDSTRDFDV | 9.97116520317156e-06 |
| Unique CDR3 aa_75 | TRSERFAY | 9.97116520317156e-06 |
| Unique CDR3 aa_76 | TRRYYYGSSGYAMDY | 9.97116520317156e-06 |
| Unique CDR3 aa_77 | TRRYSHDGFAY | 9.97116520317156e-06 |
| Unique CDR3 aa_78 | TRRRTGTGFAY | 9.97116520317156e-06 |
| Unique CDR3 aa_79 | TRRRLRLPYAMDY | 9.97116520317156e-06 |
| Unique CDR3 aa_80 | TRRRLLHAMDY | 9.97116520317156e-06 |
| Unique CDR3 aa_81 | TRRMITTGAWFAY | 9.97116520317156e-06 |
| Unique CDR3 aa_82 | TRRLRYFDV | 9.97116520317156e-06 |
| Unique CDR3 aa_83 | TRRGYYAMDY | 9.97116520317156e-06 |
| Unique CDR3 aa_84 | TRRGFDY | 9.97116520317156e-06 |
| Unique CDR3 aa_85 | TRREVRRDNAMDY | 9.97116520317156e-06 |
| Unique CDR3 aa_86 | TRRDYYGSSSFAY | 9.97116520317156e-06 |
| Unique CDR3 aa_87 | TRRDGYYGYAMDY | 9.97116520317156e-06 |
| Unique CDR3 aa_88 | TRQRGFAY | 9.97116520317156e-06 |
| Unique CDR3 aa_89 | TRPPTGTWAY | 9.97116520317156e-06 |
| Unique CDR3 aa_90 | TRPPTGTGFAY | 9.97116520317156e-06 |
| Unique CDR3 aa_91 | TRPLPY | 9.97116520317156e-06 |
| Unique CDR3 aa_92 | TRPAGSPFAY | 9.97116520317156e-06 |
| Unique CDR3 aa_93 | TRNGYGYYFDY | 9.97116520317156e-06 |
| Unique CDR3 aa_94 | TRLWDGFAY | 9.97116520317156e-06 |
| Unique CDR3 aa_95 | TRLGQGY | 9.97116520317156e-06 |
| Unique CDR3 aa_96 | TRLGLDY | 9.97116520317156e-06 |
| Unique CDR3 aa_97 | TRLGDTSGFAY | 9.97116520317156e-06 |
| Unique CDR3 aa_98 | TRKGHMGYFDY | 9.97116520317156e-06 |
| Unique CDR3 aa_99 | TRHYGYSAWFAY | 9.97116520317156e-06 |
| Unique CDR3 aa_100 | TRHYGTRN | 9.97116520317156e-06 |

**Supplementary Table 15** The top 100 CDR3 aa sequences in Bartha-K61 strain infection group.

| CDR3 ID | CDR3 Sequence (aa) | Percent |
| --- | --- | --- |
| Unique CDR3 aa_1 | YYGSSFAY | 9.97884484892029e-06 |
| Unique CDR3 aa_2 | VTVDY | 9.97884484892029e-06 |
| Unique CDR3 aa_3 | VTTGMDY | 9.97884484892029e-06 |
| Unique CDR3 aa_4 | VSWDAY | 9.97884484892029e-06 |
| Unique CDR3 aa_5 | VSQLGLAY | 9.97884484892029e-06 |
| Unique CDR3 aa_6 | VRYYYAMDY | 9.97884484892029e-06 |
| Unique CDR3 aa_7 | VRRTTVVANLYFYTMDY | 9.97884484892029e-06 |
| Unique CDR3 aa_8 | VRRLGDY | 9.97884484892029e-06 |
| Unique CDR3 aa_9 | VRQEWTGTGFAY | 9.97884484892029e-06 |
| Unique CDR3 aa_10 | VRLPDGYAMDY | 9.97884484892029e-06 |
| Unique CDR3 aa_11 | VRLGYGNAYYFDY | 9.97884484892029e-06 |
| Unique CDR3 aa_12 | VRHRGAGYYAMDY | 9.97884484892029e-06 |
| Unique CDR3 aa_13 | VRHQVTTKNYAMDY | 9.97884484892029e-06 |
| Unique CDR3 aa_14 | VRHPHNYAMDY | 9.97884484892029e-06 |
| Unique CDR3 aa_15 | VRHLFAY | 9.97884484892029e-06 |
| Unique CDR3 aa_16 | VRHGNYEYFAY | 9.97884484892029e-06 |
| Unique CDR3 aa_17 | VRHGDYDHAY | 9.97884484892029e-06 |
| Unique CDR3 aa_18 | VRHEDRRAMDY | 9.97884484892029e-06 |
| Unique CDR3 aa_19 | VRHDYDAY | 9.97884484892029e-06 |
| Unique CDR3 aa_20 | VRGYFDV | 9.97884484892029e-06 |
| Unique CDR3 aa_21 | VRGYDYAMDY | 9.97884484892029e-06 |
| Unique CDR3 aa_22 | VRGWDGFAY | 9.97884484892029e-06 |
| Unique CDR3 aa_23 | VRERRIITTVVGAMDY | 9.97884484892029e-06 |
| Unique CDR3 aa_24 | VRERRDYDYDNYFDY | 9.97884484892029e-06 |
| Unique CDR3 aa_25 | VREGVPNAMDY | 9.97884484892029e-06 |
| Unique CDR3 aa_26 | VREAYYGNAMDY | 9.97884484892029e-06 |
| Unique CDR3 aa_27 | VRDGNYWYFDV | 9.97884484892029e-06 |
| Unique CDR3 aa_28 | VGGTFAWLAY | 9.97884484892029e-06 |
| Unique CDR3 aa_29 | VCYYGSSFWYFDV | 9.97884484892029e-06 |
| Unique CDR3 aa_30 | TTYGYDGAWFAY | 9.97884484892029e-06 |
| Unique CDR3 aa_31 | TTGSYFDY | 9.97884484892029e-06 |
| Unique CDR3 aa_32 | TSPYGNYDY | 9.97884484892029e-06 |
| Unique CDR3 aa_33 | TRYYRYATPSYAMDY | 9.97884484892029e-06 |
| Unique CDR3 aa_34 | TRYGYGFDY | 9.97884484892029e-06 |
| Unique CDR3 aa_35 | TRYGYDAMDY | 9.97884484892029e-06 |
| Unique CDR3 aa_36 | TRYFDV | 9.97884484892029e-06 |
| Unique CDR3 aa_37 | TRTPPGFAY | 9.97884484892029e-06 |
| Unique CDR3 aa_38 | TRTLYYAPMDY | 9.97884484892029e-06 |
| Unique CDR3 aa_39 | TRTGTGYFDY | 9.97884484892029e-06 |
| Unique CDR3 aa_40 | TRTFAY | 9.97884484892029e-06 |
| Unique CDR3 aa_41 | TRSYYGSSYAY | 9.97884484892029e-06 |
| Unique CDR3 aa_42 | TRSTMITTFAY | 9.97884484892029e-06 |
| Unique CDR3 aa_43 | TRSRNQAWFAY | 9.97884484892029e-06 |
| Unique CDR3 aa_44 | TRSPMDY | 9.97884484892029e-06 |
| Unique CDR3 aa_45 | TRSLYDGYCAY | 9.97884484892029e-06 |
| Unique CDR3 aa_46 | TRSGYGNHFAY | 9.97884484892029e-06 |
| Unique CDR3 aa_47 | TRSGAIYAMDY | 9.97884484892029e-06 |
| Unique CDR3 aa_48 | TRSALDY | 9.97884484892029e-06 |
| Unique CDR3 aa_49 | TRRVDY | 9.97884484892029e-06 |
| Unique CDR3 aa_50 | TRRGYYGAMDY | 9.97884484892029e-06 |
| Unique CDR3 aa_51 | TRRGWYPFAY | 9.97884484892029e-06 |
| Unique CDR3 aa_52 | TRRGDGPHFDY | 9.97884484892029e-06 |
| Unique CDR3 aa_53 | TRRDYGYDY | 9.97884484892029e-06 |
| Unique CDR3 aa_54 | TRRDYGSSYYFDY | 9.97884484892029e-06 |
| Unique CDR3 aa_55 | TRRDGSRYWYFDV | 9.97884484892029e-06 |
| Unique CDR3 aa_56 | TRRDAWFAY | 9.97884484892029e-06 |
| Unique CDR3 aa_57 | TRPMIGFAY | 9.97884484892029e-06 |
| Unique CDR3 aa_58 | TRPFFDY | 9.97884484892029e-06 |
| Unique CDR3 aa_59 | TRNDGYYYFDY | 9.97884484892029e-06 |
| Unique CDR3 aa_60 | TRLAY | 9.97884484892029e-06 |
| Unique CDR3 aa_61 | TRHWEAY | 9.97884484892029e-06 |
| Unique CDR3 aa_62 | TRHWDDY | 9.97884484892029e-06 |
| Unique CDR3 aa_63 | TRHPFLDY | 9.97884484892029e-06 |
| Unique CDR3 aa_64 | TRGYSFAY | 9.97884484892029e-06 |
| Unique CDR3 aa_65 | TRGLAY | 9.97884484892029e-06 |
| Unique CDR3 aa_66 | TRGGY | 9.97884484892029e-06 |
| Unique CDR3 aa_67 | TRGGE | 9.97884484892029e-06 |
| Unique CDR3 aa_68 | TRGDGYYVIAY | 9.97884484892029e-06 |
| Unique CDR3 aa_69 | TREYYGSSYLAWFAY | 9.97884484892029e-06 |
| Unique CDR3 aa_70 | TREDGYFFAY | 9.97884484892029e-05 |
| Unique CDR3 aa_71 | TRDYYGSRYFDV | 9.97884484892029e-05 |
| Unique CDR3 aa_72 | TRDSSGYAWFAY | 9.97884484892029e-06 |
| Unique CDR3 aa_73 | TRDSEARATWFAY | 9.97884484892029e-06 |
| Unique CDR3 aa_74 | TRDRDYGSSS | 9.97884484892029e-06 |
| Unique CDR3 aa_75 | TRDGNYVPFDY | 9.97884484892029e-06 |
| Unique CDR3 aa_76 | TRDGNYPYYFDY | 9.97884484892029e-06 |
| Unique CDR3 aa_77 | TRALLRLLYAMDY | 9.97884484892029e-06 |
| Unique CDR3 aa_78 | TPTLFTY | 9.97884484892029e-06 |
| Unique CDR3 aa_79 | TNYFDY | 9.97884484892029e-06 |
| Unique CDR3 aa_80 | TNGDRYDGVAY | 9.97884484892029e-06 |
| Unique CDR3 aa_81 | TMGFAY | 9.97884484892029e-06 |
| Unique CDR3 aa_82 | TKYGNYYAMDY | 9.97884484892029e-06 |
| Unique CDR3 aa_83 | TKITTAYFDV | 9.97884484892029e-06 |
| Unique CDR3 aa_84 | TISGAY | 9.97884484892029e-06 |
| Unique CDR3 aa_85 | THYYRPDY | 9.97884484892029e-06 |
| Unique CDR3 aa_86 | TGAMITKFAY | 9.97884484892029e-06 |
| Unique CDR3 aa_87 | TETGFAY | 9.97884484892029e-06 |
| Unique CDR3 aa_88 | SRSWDGDAY | 9.97884484892029e-06 |
| Unique CDR3 aa_89 | SRSELGRDYAMDY | 9.97884484892029e-06 |
| Unique CDR3 aa_90 | SRQDYYYDGTY | 9.97884484892029e-06 |
| Unique CDR3 aa_91 | SGAHDYGAWFAY | 9.97884484892029e-06 |
| Unique CDR3 aa_92 | NWEDY | 9.97884484892029e-06 |
| Unique CDR3 aa_93 | NTEEGTARAPFAY | 9.97884484892029e-06 |
| Unique CDR3 aa_94 | NSDPFAY | 9.97884484892029e-06 |
| Unique CDR3 aa_95 | NAYYGNLYYFDY | 9.97884484892029e-06 |
| Unique CDR3 aa_96 | NAWRPAWFAY | 9.97884484892029e-06 |
| Unique CDR3 aa_97 | NAWGGGDY | 9.97884484892029e-06 |
| Unique CDR3 aa_98 | NAVYYGSWDY | 9.97884484892029e-06 |
| Unique CDR3 aa_99 | NAPFYYGYGHYTMDY | 9.97884484892029e-06 |
| Unique CDR3 aa_100 | NAGP | 9.97884484892029e-06 |

**Supplementary Table 16** The top 100 CDR3 aa sequences in control group.

| CDR3 ID | CDR3 Sequence (aa) | Percent |
| --- | --- | --- |
| Unique CDR3 aa_1 | TTGAWFAY | 9.96661185030149e-05 |
| Unique CDR3 aa_2 | NADY | 9.96661185030149e-05 |
| Unique CDR3 aa_3 | NACTGTYFDY | 9.96661185030149e-05 |
| Unique CDR3 aa_4 | ATSYYYGSSYYAMDY | 9.96661185030149e-05 |
| Unique CDR3 aa_5 | ARWGYNSIYYAMDY | 9.96661185030149e-05 |
| Unique CDR3 aa_6 | ARSSGGGIHAMDY | 9.96661185030149e-05 |
| Unique CDR3 aa_7 | ARSAY | 9.96661185030149e-05 |
| Unique CDR3 aa_8 | ARRTGYDPFDY | 9.96661185030149e-05 |
| Unique CDR3 aa_9 | ARNRGYEAWFAY | 9.96661185030149e-05 |
| Unique CDR3 aa_10 | ARENPSLID | 9.96661185030149e-05 |
| Unique CDR3 aa_11 | ARDGYYFWYFDV | 9.96661185030149e-05 |
| Unique CDR3 aa_12 | TGRTFSMDY | 9.87258721020431e-05 |
| Unique CDR3 aa_13 | ATFDY | 9.87258721020431e-05 |
| Unique CDR3 aa_14 | ASKYGNHGMDY | 9.87258721020431e-05 |
| Unique CDR3 aa_15 | ASDY | 9.87258721020431e-05 |
| Unique CDR3 aa_16 | ARYPGRRGYAMDY | 9.87258721020431e-05 |
| Unique CDR3 aa_17 | ARTGYYFDY | 9.87258721020431e-05 |
| Unique CDR3 aa_18 | ARSTLTGKGRYFDY | 9.87258721020431e-05 |
| Unique CDR3 aa_19 | ARSDYDYPDY | 9.87258721020431e-05 |
| Unique CDR3 aa_20 | ARGNYSAWFAY | 9.87258721020431e-05 |
| Unique CDR3 aa_21 | ARDGIYLLYRYYAMDY | 9.87258721020431e-05 |
| Unique CDR3 aa_22 | ARAGKDYAMDY | 9.87258721020431e-05 |
| Unique CDR3 aa_23 | AGMITAWFAY | 9.87258721020431e-05 |
| Unique CDR3 aa_24 | TRSGDSYWYFDV | 9.77856257010712e-05 |
| Unique CDR3 aa_25 | TNWDFDY | 9.77856257010712e-05 |
| Unique CDR3 aa_26 | ARYYGSSYYAMDY | 9.77856257010712e-05 |
| Unique CDR3 aa_27 | ARGRYRYDY | 9.77856257010712e-05 |
| Unique CDR3 aa_28 | ARFITTVVADY | 9.77856257010712e-05 |
| Unique CDR3 aa_29 | ARDGEDY | 9.77856257010712e-05 |
| Unique CDR3 aa_30 | VRLLWSYYAMDY | 9.68453793000994e-05 |
| Unique CDR3 aa_31 | TTGTY | 9.68453793000994e-05 |
| Unique CDR3 aa_32 | TRTDDDYYAWFAY | 9.68453793000994e-05 |
| Unique CDR3 aa_33 | TANWDWYFDV | 9.68453793000994e-05 |
| Unique CDR3 aa_34 | KRRTGTWYFDV | 9.68453793000994e-05 |
| Unique CDR3 aa_35 | ARSYRYENYYAMDY | 9.68453793000994e-05 |
| Unique CDR3 aa_36 | ARSSNFFDY | 9.68453793000994e-05 |
| Unique CDR3 aa_37 | ARLGDY | 9.68453793000994e-05 |
| Unique CDR3 aa_38 | ARGAYFDY | 9.68453793000994e-05 |
| Unique CDR3 aa_39 | TRGGNYNFDY | 9.59051328991275e-05 |
| Unique CDR3 aa_40 | ARSTMITIAY | 9.59051328991275e-05 |
| Unique CDR3 aa_41 | ARGAWFAY | 9.59051328991275e-05 |
| Unique CDR3 aa_42 | ARFDYAERLDY | 9.59051328991275e-05 |
| Unique CDR3 aa_43 | ARELTGFAY | 9.59051328991275e-05 |
| Unique CDR3 aa_44 | ARDYYFDY | 9.59051328991275e-05 |
| Unique CDR3 aa_45 | ARATGRGYAMDY | 9.59051328991275e-05 |
| Unique CDR3 aa_46 | ARALTGTAWFAY | 9.59051328991275e-05 |
| Unique CDR3 aa_47 | AKHGSSYWYFDV | 9.59051328991275e-05 |
| Unique CDR3 aa_48 | AIYYGNAMDY | 9.59051328991275e-05 |
| Unique CDR3 aa_49 | ASYYGSSYYYAMDY | 9.49648864981557e-05 |
| Unique CDR3 aa_50 | ARWIYDGYYFDY | 9.49648864981557e-05 |
| Unique CDR3 aa_51 | ARSGNYYFDY | 9.49648864981557e-05 |
| Unique CDR3 aa_52 | ARHSITTDWFAY | 9.49648864981557e-05 |
| Unique CDR3 aa_53 | ARFHYDYAMDY | 9.49648864981557e-05 |
| Unique CDR3 aa_54 | ARELDY | 9.49648864981557e-05 |
| Unique CDR3 aa_55 | AREGWLTAMDY | 9.49648864981557e-05 |
| Unique CDR3 aa_56 | ARDDGYYAMDY | 9.49648864981557e-05 |
| Unique CDR3 aa_57 | ALTGTYFDV | 9.49648864981557e-05 |
| Unique CDR3 aa_58 | YYYGYWFAY | 9.40246400971839e-07 |
| Unique CDR3 aa_59 | YYYGTSYAY | 9.40246400971839e-07 |
| Unique CDR3 aa_60 | YYYGSSYWYFDV | 9.40246400971839e-07 |
| Unique CDR3 aa_61 | YYYGSSYGYWYFDV | 9.40246400971839e-07 |
| Unique CDR3 aa_62 | YYYGSSYERVWFAY | 9.40246400971839e-07 |
| Unique CDR3 aa_63 | YYYGSSSDY | 9.40246400971839e-07 |
| Unique CDR3 aa_64 | YYYGSIAY | 9.40246400971839e-07 |
| Unique CDR3 aa_65 | YYYGSGPAY | 9.40246400971839e-07 |
| Unique CDR3 aa_66 | YYYEAHWYFDV | 9.40246400971839e-07 |
| Unique CDR3 aa_67 | YYYEAHLYFDV | 9.40246400971839e-06 |
| Unique CDR3 aa_68 | YYYDGSYAMYY | 9.40246400971839e-07 |
| Unique CDR3 aa_69 | YYYDGSFYYAMDY | 9.40246400971839e-07 |
| Unique CDR3 aa_70 | YYVSSYWYFDV | 9.40246400971839e-07 |
| Unique CDR3 aa_71 | YYVSSWFAY | 9.40246400971839e-07 |
| Unique CDR3 aa_72 | YYVPYYAMDY | 9.40246400971839e-07 |
| Unique CDR3 aa_73 | YYSNYGYAMDY | 9.40246400971839e-07 |
| Unique CDR3 aa_74 | YYSFAY | 9.40246400971839e-07 |
| Unique CDR3 aa_75 | YYGYYAMDY | 9.40246400971839e-07 |
| Unique CDR3 aa_76 | YYGYGY | 9.40246400971839e-07 |
| Unique CDR3 aa_77 | YYGYAC | 9.40246400971839e-07 |
| Unique CDR3 aa_78 | YYGSTYWYFDV | 9.40246400971839e-07 |
| Unique CDR3 aa_79 | YYGSTWFAY | 9.40246400971839e-07 |
| Unique CDR3 aa_80 | YYGSSYYWYFDV | 9.40246400971839e-07 |
| Unique CDR3 aa_81 | YYGSSYWYFGV | 9.40246400971839e-07 |
| Unique CDR3 aa_82 | YYGSSYWYFDL | 9.40246400971839e-07 |
| Unique CDR3 aa_83 | YYGSSYWNFDV | 9.40246400971839e-07 |
| Unique CDR3 aa_84 | YYGSSYVDY | 9.40246400971839e-07 |
| Unique CDR3 aa_85 | YYGSSYRYFDV | 9.40246400971839e-07 |
| Unique CDR3 aa_86 | YYGSSYGYFDY | 9.40246400971839e-07 |
| Unique CDR3 aa_87 | YYGSSYGFAY | 9.40246400971839e-07 |
| Unique CDR3 aa_88 | YYGSSYDY | 9.40246400971839e-07 |
| Unique CDR3 aa_89 | YYGSSYAMDY | 9.40246400971839e-07 |
| Unique CDR3 aa_90 | YYGSSSWYFDV | 9.40246400971839e-07 |
| Unique CDR3 aa_91 | YYGSSSFAY | 9.40246400971839e-07 |
| Unique CDR3 aa_92 | YYGSSPWFAY | 9.40246400971839e-07 |
| Unique CDR3 aa_93 | YYGSSLFDV | 9.40246400971839e-07 |
| Unique CDR3 aa_94 | YYGSSHWYFDV | 9.40246400971839e-07 |
| Unique CDR3 aa_95 | YYGSSDWYFDV | 9.40246400971839e-07 |
| Unique CDR3 aa_96 | YYGSRYYFDY | 9.40246400971839e-07 |
| Unique CDR3 aa_97 | YYGSRYWYFDV | 9.40246400971839e-07 |
| Unique CDR3 aa_98 | YYGSIAY | 9.40246400971839e-07 |
| Unique CDR3 aa_99 | YYGSGYWYFDV | 9.40246400971839e-07 |
| Unique CDR3 aa_100 | YYGNYYAMDY | 9.40246400971839e-07 |
